# Association mapping of wheat distinctness, uniformity, and stability traits identifies evidence of *TaDof-B* copy number variation associated with stem pith thickness

**DOI:** 10.3389/fpls.2026.1739489

**Published:** 2026-03-31

**Authors:** Bethany Love, Pauline Bansept-Basler, Tobias Barber, James A. Bedford, Simon Berry, Nick Bird, Finn Borum, James Brown, Ruth Bryant, Tansy Chia, John Connell, Paul Fenwick, David Feuerhelm, Ed Flatman, Nick Gosman, Charlotte Hayes, Tina Henriksson, Peter Jack, Matt Kerton, Jacob Lage, Vanessa McMillan, Linda Kærgaard Nielsen, Lawrence Percival-Alwyn, Jörg Schondelmaier, Rajiv Sharma, Stephen Smith, Phillip Tailby, Pernilla Vallenback, Margaret Wallace, Duncan Warner, Tally I.C. Wright, Chin Jian Yang, Camila M. Zanella, Keith A. Gardner, Ian J. Mackay, Donal M. O’Sullivan, James Cockram

**Affiliations:** 1 NIAB, Cambridge, United Kingdom; 2 Limagrain UK, Market Rasen, United Kingdom; 3 KWS UK Ltd, Thriplow, United Kingdom; 4 Sejet Planteforædling, Horsens, Denmark; 5 John Innes Centre, Norwich, United Kingdom; 6 RAGT Seeds Ltd, Saffron Walden, United Kingdom; 7 Syngenta UK Ltd, Cambridge, United Kingdom; 8 Elsoms Wheat Ltd, Spalding, United Kingdom; 9 Lantmännen Lantbruk, Svalöv, Sweden; 10 DSV UK Ltd, Banbury, United Kingdom; 11 Saaten-Union Biotec GmbH, Nordrhein-Westfalen, Leopoldshoehe, Germany; 12 Scotland’s Rural College (SRUC), Edinburgh, United Kingdom

**Keywords:** Community Plant Variety Office (CPVO), genome-wide association study (GWAS), International Union for the Protection of New Varieties of Plants (UPOV), single nucleotide polymorphism (SNP) array, variety protection

## Abstract

For new varieties to be sold, they must first pass assessment using a set of phenotypic criteria that test for distinctness, uniformity, and stability (DUS) and which serve as the basis for the awarding of plant breeders’ rights. The objective of this study was to use historical DUS phenotypic data to investigate the genetic architecture of DUS characteristics in wheat (*Triticum aestivum* L.). Using a panel of 334 varieties, genome-wide association studies (GWAS) identified significant marker–trait associations for 18 of the 33 wheat DUS characteristics investigated. The most significant was the genetic locus *P22_3B_821* for stem pith thickness, located between 818 and 830 Mb on chromosome 3B. Haplotype analysis informed conversion of three genetic markers selected from the genotyping array to the KASP genotyping platform, allowing alleles associated with low–medium versus thick pith thickness to be easily tracked. Subsequent genomic and molecular analysis found evidence that *TaDof-B* copy number variation (CNV) may underlie control of pith thickness in wheat, whereby CNV ≥3 was associated with the solid stem phenotype, analogous to that previously observed for *TdDof-B* in durum wheat (*T. turgidum* subsp. *durum*) and lower CNV with hollow stems. Finally, correlations between DUS characteristics with yield, grain quality traits, and year of release indicated that reduced ear density is under breeder selection and that this has a beneficial effect on the quality traits ‘grain test weight’ and ‘Hagberg falling number’. Collectively, these findings and molecular tools will help inform commercial, DUS regulatory and scientific advances in future wheat research and development programmes.

## Introduction

Wheat (*Triticum aestivum* L.) is the world’s third most important crop based on global yields (FAO, 2022). Accordingly, its efficient cultivation and harvest across the wheat-growing regions of the world plays a key role in ensuring global food security. The development of a new wheat variety represents a considerable investment by breeders, typically taking approximately 10 years. Such investment is sustained by commercial returns on the sale or use of new varieties, underpinned by protection of plant breeders’ intellectual property rights awarded via an international framework coordinated by the International Union for the Protection of New Varieties of Plants (UPOV) (https://www.upov.int/). Protection within the UPOV system, and variety registration, requires DUS phenotypic testing of new ‘candidate’ varieties, carried out in each UPOV member country via national reporting authorities and test facilities. The overarching aim of the DUS process is to demonstrate that new candidate varieties are distinct (D) from all other existing varieties of common knowledge (the variety collection) based on a set of DUS phenotypic ‘characteristics’ and to demonstrate they are uniform (U) and stable (S) in their expression of these DUS characteristics (Jones et al., 2013). For wheat, there are currently 27 UPOV DUS characteristics to verify D, as well as three protein electrophoresis characteristics, which can be used as a complement to the morphological characteristics (UPOV, 2017) (**Table 1**, **Supplementary Table 1**). These DUS characteristics are assessed over a minimum of two growing seasons, with a third year of test sometimes required to unambiguously establish distinctness from the varieties within the variety collection. In practice, testing is undertaken against the wheat ‘Technical Variety Collection’ representing ~2,000 varieties from the wider worldwide Theoretical Variety Collection of ~11,000 varieties of common knowledge. Attaining DUS accreditation enables the awarding of plant breeders’ rights (PBR), a stand-alone system for intellectual property protection applicable to newly developed crop and horticultural varieties (Jones et al., 2013). For farmers and consumers to benefit from the genetic gains made by plant breeders, all new candidate varieties released in the 78 signatory countries and organisations of the UPOV convention must first pass DUS accreditation. In many countries, the marketing of new wheat varieties is reliant on variety listing or registration, which also requires similar DUS testing (often alongside value for cultivation and use testing). This puts the DUS accreditation system at the heart of the processes that collectively deliver food security in countries across the world.

**Table 1:** Wheat DUS characteristics included in this study and their heritabilities (*h^2^*).

Trait code	DUS characteristic name	Entries	*h*^2^ (s.e.)	No. GWAS hits^†^
P1	Coleoptile – anthocyanin colouration	304	0.25 (0.07)	1 (1)
P2	Plant – growth habit	312	0.21 (0.07)	
P6	Plant – frequency of plants with recurved flags	293	0.25 (0.07)	1
P8	Time of ear emergence (1st spikelet visible on 50% ears	306	0.22 (0.07)	
P9	Flag leaf – anthocyanin colouration of auricles	256	0.26 (0.08)	
P10	Flag leaf – attitude	104	0.41 (0.16)	
P14	Flag leaf – glaucosity of sheath	316	0.23 (0.07)	4 (3)
P15	Flag leaf – glaucosity of blade (lower side)	191	0.19 (0.09)	3 (1)
P16	Culm – glaucosity of neck	323	0.20 (0.06)	5 (4)
P17	Ear – glaucosity	323	0.25 (0.07)	3 (1)
P18	Supernumerary spikelet – frequency	230	0.17 (0.07)	
P21	Plant – length (stems, ears, awns, and scurs)	305	0.19 (0.06)	1
P22	Straw – pith in cross section^*^	332	0.58 (0.08)	3 (2)
P26	Awns or scurs – presence	332	0.34 (0.07)	2 (1)
P27	Awns or scurs – distribution	299	0.05 (0.05)	
P28	Awns or scurs at the tip of ear – length	314	0.33 (0.07)	1
P30	Ear – shape in profile	304	0.12 (0.06)	
P31	Ear – length (excluding awns or scurs)	298	0.24 (0.07)	1
P33	Ear density	323	0.11 (0.05)	
P41	Lower glume – beak length	319	0.11 (0.05)	
P42	Lower glume – beak shape	317	0.16 (0.06)	
P43	Lower glume – beak tip	214	0.07 (0.06)	
P45	Lower glume – shoulder width	321	0.27 (0.07)	1
P46	Lower glume – shoulder shape	302	0.18 (0.06)	1
P51	Lower glume – external surface roughness	326	0.21 (0.06)	2 (1)
P52	Lower glume – extent of internal hairs	328	0.52 (0.08)	
P55	Lowest lemma – beak shape	313	0.26 (0.07)	
P56	Lowest lemma – beak length	247	0.14 (0.06)	
P58	Lowest lemma – beak swelling	226	0.18 (0.08)	3 (1)
P68	Apical rachis segment – hairiness of the convex surface	297	0.25 (0.07)	
P79	Grain – colouration with phenol	322	0.49 (0.08)	2 (2)
P80	Seasonal type	317	0.21 (0.07)	12 (9)
P100	Consensus seasonal type	333	0.17 (0.06)	12 (9)

DUS characteristic codes used in the current UPOV system are in bold; those used in the current CPVO system (according to guidelines CPVO-TP/003/4) are underlined. The number of genome-wide association study (GWAS) hits for each characteristic is also shown. s.e., standard error. ^*^Between the ear and the upper node. ^†^The number of GWAS hits above the Bonferroni-corrected *q* = 0.05 significance threshold is indicated in parentheses.

For inbred crop species such as wheat, the use of molecular markers within DUS processes can help breeding companies ensure new variety candidates do not inadvertently segregate for DUS traits, and potentially help future processes such as assessment of DUS characters, seed stocks, and management of variety collections. Indeed, UPOV recognised the potential of the application of molecular markers to DUS when it established the Biochemical and Molecular Techniques (BMT) working group [and continues through the Technical Working Party on Testing Methods and Techniques (TWM)], which suggests several models for their implementation within variety registration (UPOV document INF/18/1/2011). Accordingly, there has been interest within the crop research community in the development of resources, knowledge, and analytical approaches for the use of molecular markers for DUS (reviewed by Jamali et al., 2019). In the related cereal crop barley (*Hordeum vulgare* L.), this includes the identification of molecular markers via genome-wide association study (GWAS) (Cockram et al., 2010; Ramsay et al., 2011; Wang et al., 2012; Yang et al., 2021), development of genetic markers diagnostic for DUS traits (Cockram et al., 2009, 2012, 2015), suitability of molecular markers for the detection of DUS off-types (Saccomanno et al., 2020), and the exploration of the use of genome-wide genetic markers to establish thresholds to distinguish varieties previously established as distinct via DUS (Jones et al., 2013). Notably, there is evidence that the DUS combinatorial space is becoming limited within the spring barley genepool (Yang et al., 2021). Whilst the genetic determinants and alleles controlling ‘spring’ or ‘winter’ seasonal type (UPOV character 26) in wheat and barley are well known (e.g., Yan et al., 2004, 2006; Cockram et al., 2007), to date, we are not aware of any published studies that have specifically aimed to systematically identify genetic markers tagging the suite of DUS traits currently used for wheat. Such markers could help streamline DUS-related processes, for example, by addressing phenotyping subjectivity and environmental effects, as well as reducing phenotyping costs. GWAS is a potentially well-suited methodology to identify genetic loci controlling DUS traits, due to the availability of historic DUS phenotypic data generated during the varietal registration process. Here, we use a panel of 334 wheat varieties genotyped with a high-density genotyping array to undertake GWAS of DUS characteristics.

The principal objectives of this study were to 1) identify linked molecular markers for potential downstream applications; 2) use one of the identified genetic loci as an exemplar for further molecular investigation; 3) explore the suitability of DUS characteristics for their intended trait, via analysis of heritability and correlations with key agronomically important traits; and 4) discuss the implications of the findings for breeders and variety testing offices and wheat research and development.

## Materials and methods

### Germplasm and genotyping

The wheat association mapping panel and genotypic data used in this study were previously reported—including all information on data quality control and imputation of missing data (Gardner et al., 2025). Briefly, the source data-matrix consisted of 480 North-west European wheat varieties genotyped with an Illumina 90,000 feature SNP array (Wang et al., 2014a), resulting in 26,015 polymorphic SNPs. Of these, 18,749 have been mapped to the wheat genetic (Gardner et al., 2016) and/or physical (IWGSC et al., 2018) maps, as described by Gardner et al. (2025). All genotypic data are available online at www.niab.com/resources. Wherever possible, the application for protection (AFP) number (a unique numerical identifier allocated to all new candidate varieties when they are submitted to the UK Plant Variety and Seeds Office for DUS accreditation) is recorded for each accession.

### DUS characteristics and heritability

Of the 480 varieties previously genotyped in our association mapping panel (Gardner et al., 2025), permissions were obtained from breeding companies for inclusion of DUS data for 402 varieties, of which DUS data were available to this study for 334 varieties. For each of these, the consensus phenotypic data for DUS characteristics recorded over at least 2 years of assessment at the time of candidate variety submission were sourced from historical electronic records held at Niab, UK (**Supplementary Table 2**), with permissions from the relevant breeding companies. Varieties with permissions but with no electronic DUS records were cross-referenced against historical paper records held at Niab and updated as necessary in **Supplementary Table 2**. DUS characteristics were scored according to national and international guidelines established by UPOV (UPOV Test Guideline TG/3/12, available online at https://www.upov.int/test_guidelines/en/list.jsp), the Community Plant Variety Office (CPVO) (CPVO-TP/003/4, available at https://cpvo.europa.eu/en), and the UK national authorities (the current UK wheat protocol, dated April 2025, available online at https://www.gov.uk/guidance/dus-protocols-for-testing-plant-varieties). The scoring system for each characteristic is listed in **Supplementary Table 1**. DUS data for additional wheat cultivars, which possess genome assemblies, were sourced from public records (**Supplementary Table 2**). In total, there were 90 DUS characteristics in the original dataset (**Supplementary Table 1**). Four national DUS characteristics had no data for any of our 334 varieties, and two were monomorphic and so were removed from forward analyses. Additionally, 47 characteristics were removed due to having data for less than 100 varieties. Finally, three characteristics were removed as they showed almost no variation in character value—including the UPOV/CPVO characteristics ‘ear – colour’ (for which all but two varieties were scored as ‘white’) and ‘grain – colour’ (for which all but three varieties were scored as ‘red’). Accordingly, the final phenotypic data-matrix consisted of 334 wheat varieties and 33 DUS characteristics (**Table 1**), of which 24 are currently available for the awarding of plant breeders’ rights via UPOV guidelines, 26 used in the UK, and 22 via CPVO. Histograms of DUS characteristic phenotypic states were made using the ‘ggplot2’ (Wickham, 2016) and ‘tidyr’ (Wickham et al., 2024) packages in RStudio v4.4.1 (RStudio Team, 2020). Narrow-sense heritability (*h*^2^) was determined using the standard univariate mixed linear model to partition the phenotypic variance for each DUS trait into additive genetic and residual variances. The model details are similar to a previously used model for estimating genomic heritabilities in barley DUS traits (Yang et al., 2021). Further details are available in the **Supplementary Text**.

### Phenotypic correlations

Pearson’s correlation coefficients (*r*) between DUS characteristics were calculated using the *rcorr* function in ‘Hmisc’ (Harrell, 2024) and plotted using ‘ggplot2’. For the estimation of genetic correlations between DUS and non-DUS traits, we used the standard bivariate mixed linear model. The non-DUS traits include grain yield, three measures of grain quality [Hagberg falling number (HFN), grain protein content, and test weight], and year of variety release. These data were sourced as best linear unbiased estimates (BLUEs) from the 150 wheat varieties investigated by White et al. (2022). We merged the DUS and non-DUS datasets based on their AFP numbers to obtain a combined dataset with 146 varieties. The model details were similar to a previously used model for estimating genetic correlations between barley DUS traits and grain yield (Yang et al., 2021). Briefly, we fitted two models for a pair of traits, each with a fixed intercept and a random additive genetic term. The random terms from the pair of models were assumed to share a bivariate normal distribution with mean 0 and variance–covariance structure proportional to the additive genetic relationship matrix derived from 146 wheat lines. Model fitting used the *mmer* function in the ‘sommer’ package. We used the *vpredict* function in the same package to obtain the estimates and standard errors for additive genetic correlation, which was calculated as genetic covariance divided by the square root of the product of genetic variances from both traits.

### GWAS and power analyses

The genotypic and phenotypic datasets were first parsed so that only those subsets of genotyped varieties that possessed DUS phenotypic data were retained. As the amount of missing phenotypic data varied for each DUS characteristic, for downstream analyses, individual genotypic subsets were created such that each trait was represented by a genotypic dataset only for those varieties for which phenotypic data were available. These genotypic datasets, unique to each trait, were then each skimmed to remove markers with a minor allele count of less than 12 individuals (judged as representing the lower boundary for reliably associating an effect with a small sample size), before the removal of one marker from each pair of markers with 100% correlation. This process was done separately for physically mapped and unmapped markers. DUS characteristics with a data fill of less than 100 varieties or which showed very little meaningful variation in character states were removed from downstream analyses. GWAS was performed in RStudio using the package GAPIT v3.5 (Lipka et al., 2012), implementing a mixed linear model (MLM), and was performed separately for physically mapped and unmapped markers. The confounding effects of population genetic stratification was accounted for using methods implemented in GAPIT: i) population structure as a fixed effect, via the use of five principal components (PCs), and ii) kinship as a random effect, determined using a subset of SNPs skimmed from the genotypic dataset unique to each trait, described above, using a correlation threshold of 0.75. Two significance thresholds for marker–trait associations were used: i) an exploratory threshold of −log_10_P = 4 and ii) the Bonferroni threshold, calculated as −log_10_(*α*/number of markers) where *α* = 0.05. Genetic marker order in Manhattan plots of marker–trait associations across the genome was ordered according to physical map position in the wheat reference genome (RefSeq v1.0; IWGSC et al., 2018). Genomic inflation factors (*λ*) were calculated (Devlin and Roeder, 1999) and added to the qq-plots to assess inflation of *p*-values. Marker–trait associations were consolidated into discrete quantitative trait loci as described by Gardner et al. (2025) and summarised visually using a chromosomal ideogram constructed using the R package ‘LinkageMapView’ (Ouellette et al., 2018).

To investigate the detection power of the MLM on the association mapping panel, 10 traits with 100 replicates were produced from the skimmed genotype data using the function *create_phenotypes* from the R package ‘simplePHENOTYPES’ (Fernandes and Lipka, 2020). Each trait was simulated to be controlled by 10 additive quantitative trait nucleotides (QTNs), with the parameters: *add_effect = 0.5*, *big_add_QTN_effect = 0.9*, *rep = 100, MAF > 0.05*, and *vary_QTN = TRUE*. Ten levels of trait heritability were simulated, with values ranging from 0.05 to 0.9. With these parameters, the proportion of genetic variance explained (PGVE) of the top 3 QTNs corresponded on average to approximately 71%, 23%, and 6% of the overall genetic variance. This allowed the panel to be evaluated for its ability to detect large, medium, and small effect QTNs. For each heritability level and replicate, the phenotype data were randomly thinned to 50, 100, 150, 200, 250, 300, and 334 individuals. The genotype data were subset, reskimmed, and filtered at a minor allele count of 12. GWAS was performed using an MLM with GAPIT, with relatedness between individuals accounted for by incorporating a kinship matrix generated from genotype data skimmed at a correlation threshold of 0.75. Each target QTN was compared against SNPs passing the false discovery rate (FDR) threshold (*α* = 0.05) and scored as detected if an FDR-significant SNP was present within ±20 Mb of the target QTN position. The average detection rate was then calculated across 100 replicates.

### Bioinformatics

*TdDof* orthologues and paralogues were identified in wheat and other Poaceae species using *TdDof* CDS as a query for BLASTn searches of relevant genome assemblies curated at Ensembl Plants (Harrison et al., 2024). Phylograms were generated using Clustal Omega (Sievers et al., 2011). For estimation of copy number variation (CNV) via Illumina sequence read depth analysis, paired-end reads from wheat cvs. ‘Cadenza’, ‘Chinese Spring’, ‘Claire’, ‘Robigus’, ‘Paragon’, and ‘Weebill1’ (Clavijo et al., 2017; Walkowiak et al., 2020) were first trimmed using trim-galore (Krueger, 2019) to remove adapter sequences and quality filtered (-q 25) to retain only reads with a length ≥100 bp before aligning with BWA mem (Li, 2013) against the wheat reference genome assembly of cv. ‘Chinese Spring’ (CS) (RefSeq v1.0, IWGSC et al., 2018). Following BWA alignment, SAMtools fixmate was first used to fix and fill in mate information before filtering to retain proper read pairs and remove non-primary alignments using SAMtools view (Li et al., 2009). The alignments were then sorted with SAMtools before removing sequencing duplicates using Picard MarkDuplicates (http://broadinstitute.github.io/picard). BAMs for each cultivar were then merged and indexed (-c) using SAMtools. For subsequent mapped sequence depth analysis, sample alignment depths for chromosome (chr) 3B regions 828000748–828207481 and 828110723–828112506 (RefSeq v1.0) were extracted for all positions using SAMtools depth (-a -Q 20). Sample depths were then normalised using a custom Python script by median depth, calculated from depth values within the range of 10–90× for each sample from the chr3B 828000748–828207481 region. Finally, read depths were smoothed by averaging a sliding (every 100 bp, 1 bp) window (1,000 bp, 50 bp in size) for the larger and smaller regions, respectively, before being plotted in R. Further annotation of the 828000748–828207481 region of chromosome 3B immediately surrounding *TaDof-B* was undertaken as described in the **Supplementary Text**.

### Molecular marker development

Kompetitive allele-specific PCR (KASP) molecular marker development was undertaken as described in the **Supplementary Text**, using the primers listed in **Supplementary Table 3**. Real-time PCR TaqMan™ assays for assessment of *TaDof-B* copy number variation were undertaken using amplification primers designed to detect *TraesCS3B02G608800* as the gene of interest and *GAMYB* transcription factor (*TraesCS3B02G367500*) as the normaliser gene (primer details are listed in the **Supplementary Text**). Both genes were assayed in multiplex using a QuantStudio 7 Real-Time PCR system (Applied Biosystems, Warrington, UK) using Thermo ABGene ABsolute qPCR Rox Mix (Fisher Scientific, UK) using the reaction volumes and thermal cycling conditions detailed in the **Supplementary Text**. A minimum of three technical replicates was undertaken per genotype. Gene copy number was calculated using the delta C_t_ values of *TraesCS3B02G608800* normalised against the normaliser, *GAMYB*, during the exponential phase of amplification.

## Results

### DUS characteristic phenotypic analysis

The data-matrix analysed consisted of 334 varieties and 33 DUS characteristics (**Table 1**), which included 24 of the 30 DUS morphological and protein electrophoresis characteristics assessed in the current wheat UPOV test guidelines (**Supplementary Table 2**). Histograms illustrating DUS characteristic score distributions are illustrated in **Supplementary Figure 1**. Whilst the mean number of varieties with DUS data varied slightly from trait to trait (**Table 1**), the fill was generally high (mean = 292, median = 312), with the highest fill observed for P22 (‘Straw – pith in cross section’, 332 varieties), P26 (‘Awns or scurs – presence’, 332 varieties), and P100 (‘Consensus seasonal type’, 333 varieties). Over two-thirds of the DUS characteristics were scored on a 1–9 scale, with the remaining 12 characteristics scored using scales of either 7 (P52, P58), 5 (P27, P30, P43, P56), 4 (P51), or 3 (P22, P26, P33, P80, P100) notes (**Supplementary Table 1**).

**Figure 1:**
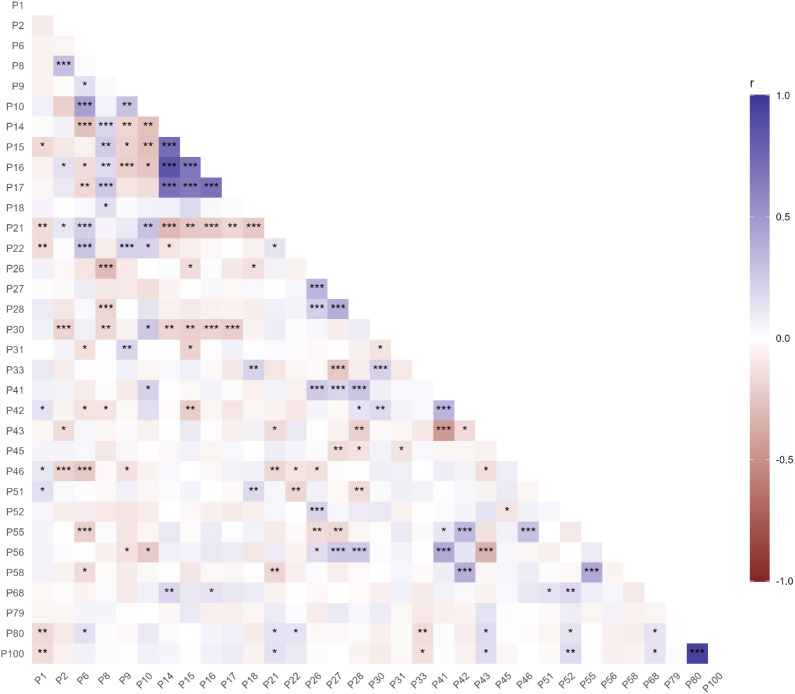
Correlations (Pearson’s correlation coefficient, *r*) between DUS characteristics. Significance: *p* = 0.05^*^, *p* = 0.01^**^, *p* = 0.001^***^.

Heritabilities of the 33 DUS characteristics investigated varied considerably, with values for current UPOV DUS characteristics ranging from 0.58 (P22 ‘Straw – pith in cross section’) down to 0.11 (P41 ‘Lower glume – beak length’) (**Table 1**). In addition to P22, DUS characteristics notable for their relatively high heritability (*h^2^* > 0.45) included ‘lower glume – extent of internal hairs’ (P52) and ‘grain – colouration with phenol’. Correlations (Pearson’s correlation coefficient, *r*) were found to be present between some DUS characteristics (**Figure 1**), with many of these likely due to being obviously related in their phenotypic nature. For example, high positive correlations were observed between glaucosity measurements on the leaf sheath, blade, culm, and ear (P14, P15, P16, P17; *r* > 0.65, *p* > 0.001). Similarly, highly significant positive correlations were observed for ‘Lower glume – beak length’ (P41) and ‘Lowest lemma – beak length’ (P56) with the three awn/scur phenotypes (P26, P27, P28) (*r* > 0.20, *p* > 0.001). Other trait correlations were not so obviously related in nature, including i) significant positive correlation between ear emergence (P8) and some of the glaucosity traits (P14, P17) and ‘Plant – growth habit’ (P2) (*r* > 0.19, *p* > 0.01), and ii) significant negative correlation between glaucosity traits and ‘Plant length’ (P21) (*r* < −0.17, *p* > 0.01).

**Figure 2:**
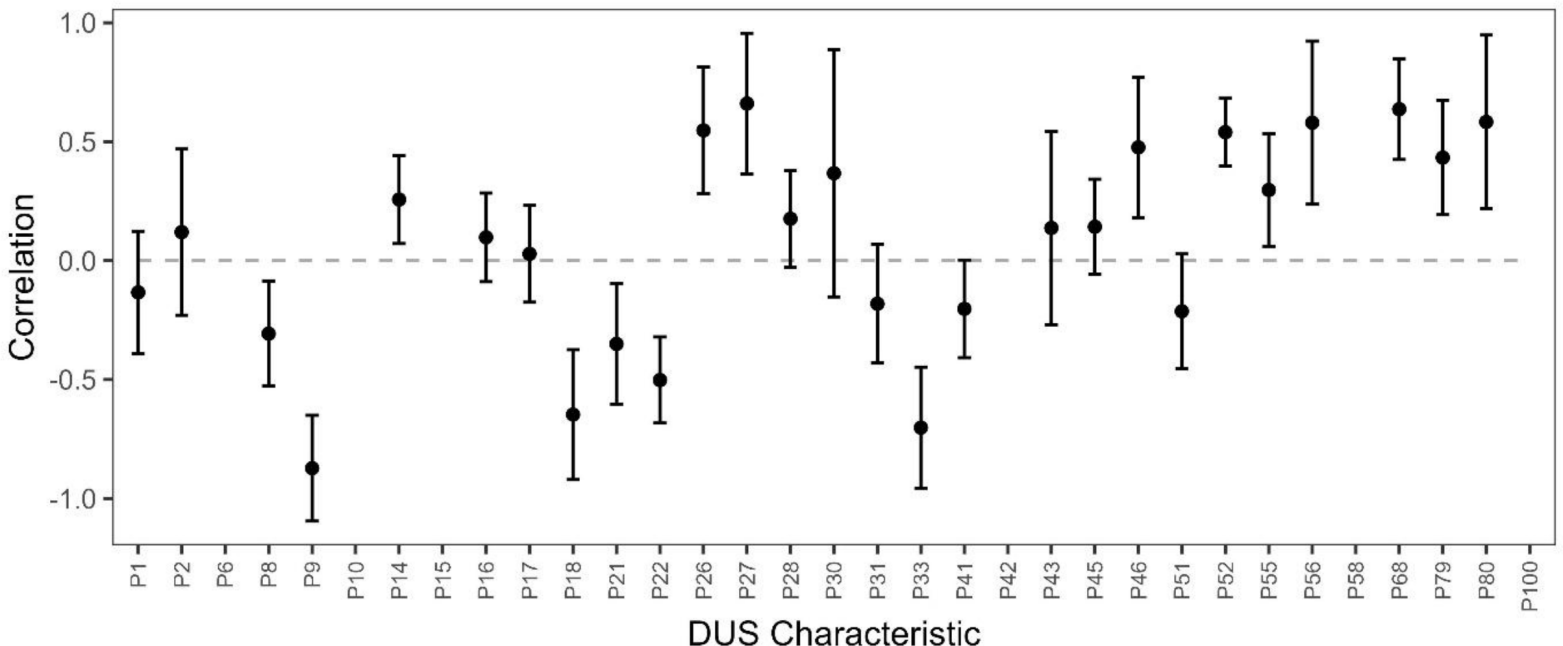
Genetic correlation between DUS characteristics and year of variety release. Bars indicate standard errors. DUS characteristic codes are as described in **Supplementary Table 1**, including the following characters notably correlated with year of release: P9 (‘Flag leaf – anthocyanin colouration of auricles’), P26 (‘Awns or scurs – presence’), P27 (‘Awns or scurs – distribution), P33 (‘Ear density’), P46 (‘Lower glume – shoulder shape’), P52 (‘Lower glume – extent of internal hairs’), P56 (‘Lowest lemma – beak length’), P68 (‘Apical rachis segment – hairiness of convex surface’), P79 (‘Grain – colouration with phenol’), and P80 (‘Seasonal type’). Traits that failed to converge in the bivariate model do not have estimates of genetic correlations but are included on the *x*-axis.

Analysis of genetic correlations between the 33 DUS characteristics and year of cultivar release showed that 52% of the traits analysed showed non-zero correlations, highlighting shifts in the character states of DUS characteristics over the 40-year period analysed (**Figure 2**). Most notable amongst these were strong positive correlations for ‘Awns or scurs – presence’ (P26), ‘Awns or scurs – distribution’ (P27), ‘Lower glume – shoulder shape’ (P46), ‘Lower glume – extent of internal hairs’ (P52), ‘Lowest lemma – beak length’ (P56), ‘Apical rachis segment – hairiness of convex surface’ (P68), ‘Grain – colouration with phenol’ (P79), and ‘Seasonal type’ (P80) and strong negative correlations between year of release and ‘Flag leaf – anthocyanin colouration of auricles’ (P9), ‘Supernumerary spikelet- frequency (P18), and ‘Ear density’ (P33).

Over time, cereal crop breeding has resulted in genetic gains in grain yield, including for European wheat (e.g., Mackay et al., 2011). To further explore whether the correlations between DUS characteristics and year of variety release may also be related to genetic gains in wheat grain yield and grain quality traits, we explored genetic correlation between the 33 DUS characteristics investigated and previously published data for grain yield and three grain quality traits (**Supplementary Table 4**): grain protein content (GPC), Hagberg falling number (HFN, a measure of alpha-amylase enzymatic activity on starch properties of wheat flour, with higher HFN numbers preferred for breadmaking as an indicator of reduced starch breakdown), and test weight (TW, also known as specific weight) (White et al., 2022). For those comparisons based on data for 100 or more varieties, and using the subset of DUS characteristics in our dataset currently in use by UPOV and CPVO, notable positive correlations were observed between grain protein content and ‘Plant – frequency of plants with recurved flags’ (P6, *r =* 0.71) and between yield versus ‘Lower glume – shoulder shape’ (P46, *r =* 0.72). Notable negative correlations were identified between yield versus ‘Flag leaf – anthocyanin colouration of auricles’ (P9, *r =* −0.82), yield versus ‘Straw – pith in cross section’ (P22, *r =* −0.80), and grain test weight versus ‘Ear density’ (P33, *r =* −0.81).

### GWAS of wheat DUS traits

To investigate the statistical power of the association mapping panel for the detection of SNP–phenotype associations, traits were simulated to be controlled by 10 QTNs, at 10 levels of trait heritability. Each heritability level was replicated a hundred times and tested across seven panel sizes, resulting in 7,000 GWAS runs. As expected, QTN detection rate increased with the panel size and trait heritability. At low values of trait heritability (0.05 and 0.1), QTN detection is low across all panel sizes (**Supplementary Figure 2**). For major effect QTNs (represented in **Supplementary Figure 2** as QTN1), QTN detection is at least 50% for panel sizes above 200 and a heritability of 0.2. This increases to approximately 100% detection at heritabilities ≥0.3. The detection of minor-effect QTNs (represented in **Supplementary Figure 2** as QTN2 and QTN3) is reduced; however, it remains possible when analysing highly heritable traits across large association mapping panels.

**Figure 3:**
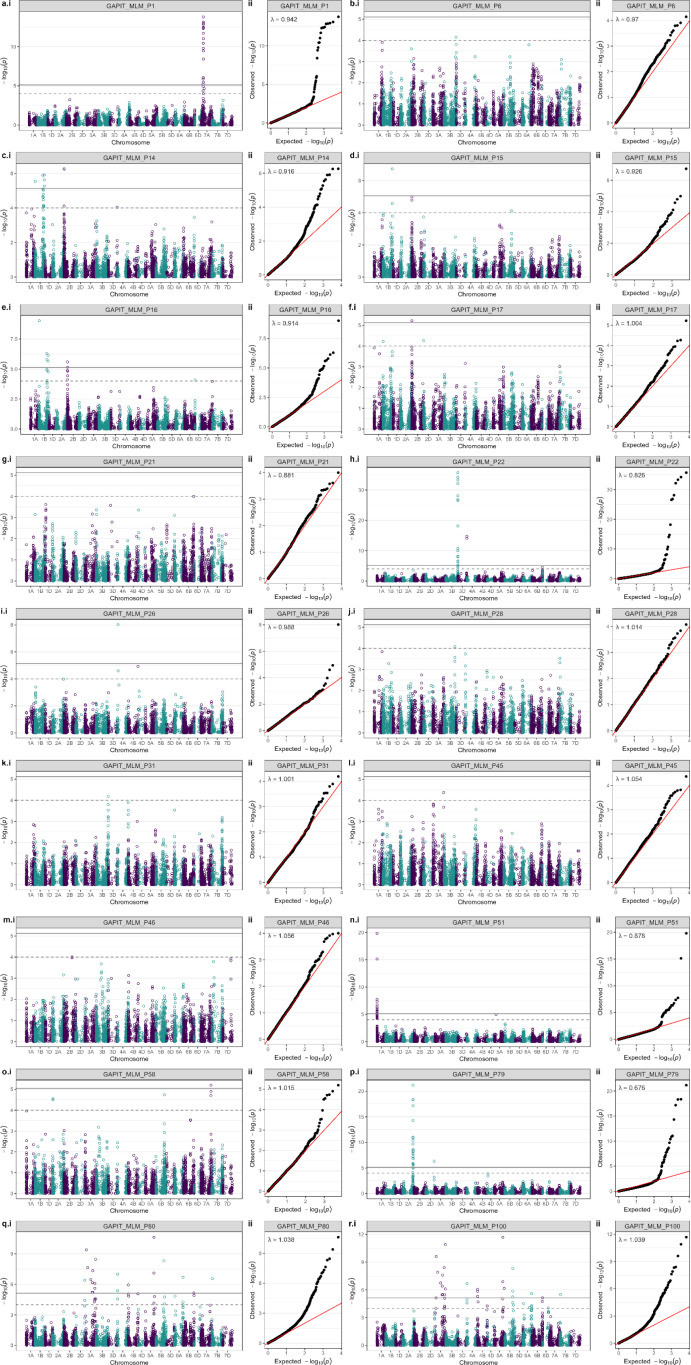
Genome-wide association study (GWAS) results of the 18 Distinctness, Uniformity and Stability (DUS) characteristics for which significant genome-wide association study (GWAS) hits were identified using a Bonferroni-corrected *α* = 0.05 significance threshold (solid line) and/or an exploratory significance threshold (−log_10_P = 4, dashed line). Manhattan (i) and quantile–quantile (ii) plots are shown. DUS traits: **(A)** P1, **(B)** P6, **(C)** P14, **(D)** P15, **(E)** P16, **(F)** P17, **(G)** P21, **(H)** P22, **(I)** P26, **(J)** P28, **(K)** P31, **(L)** P45, **(M)** P46, **(N)** P51, **(O)** P58, **(P)** P79, **(Q)** P80, and **(R)** P100.

**Figure 4:**
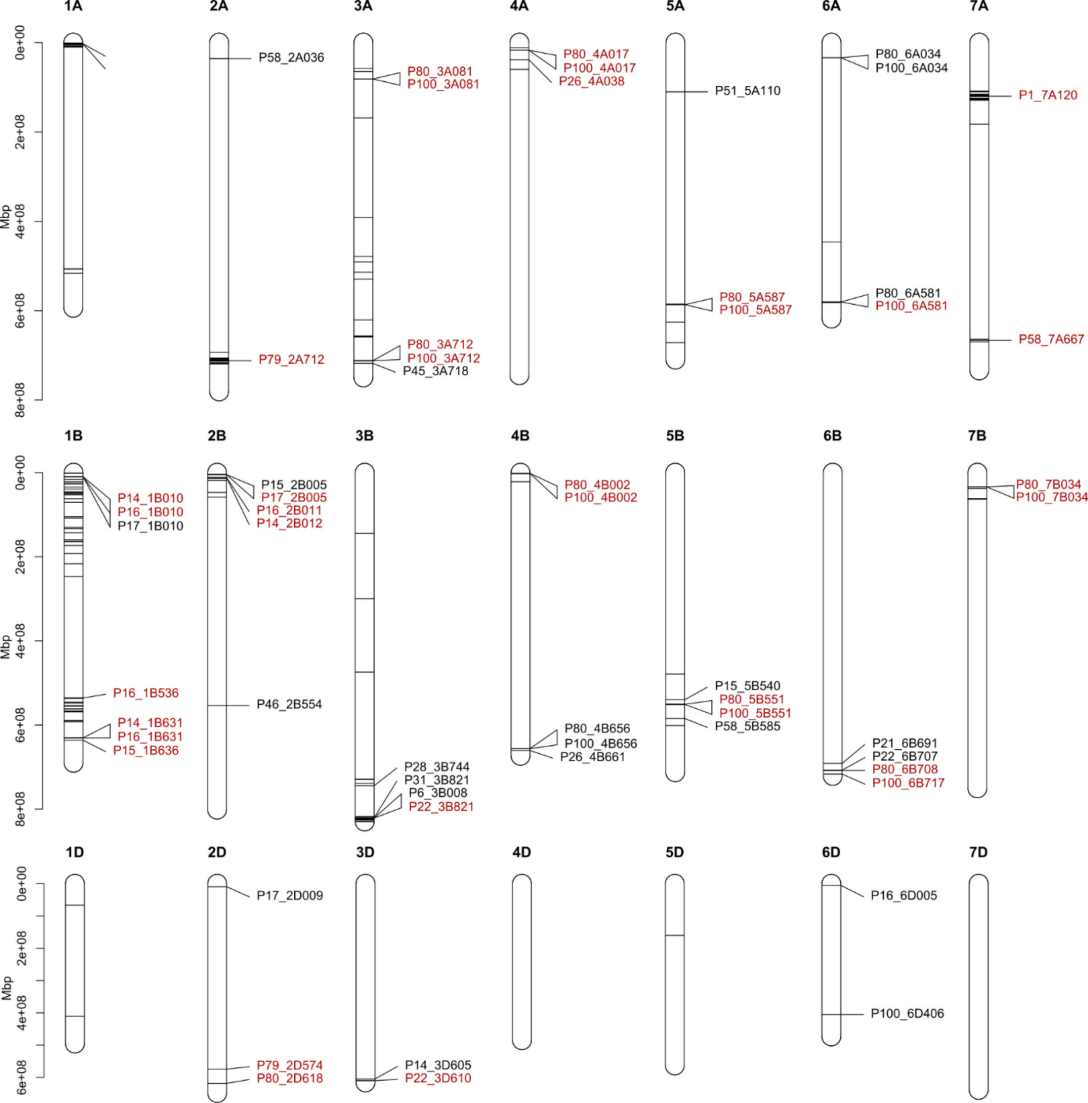
Location on the physical map of the wheat reference genome (cv. Chinese Spring, assembly RefSeq v1.0; IWGSC et al., 2018) of the most significant genetic marker for each genetic locus identified by genome-wide association study (GWAS) analysis of DUS characteristics. Genomic locations shown are in the format PX_Y_Z, where PX = DUS trait characteristic number, Y = chromosome, and Z = Mb position on the chromosome. Associations significant based on the exploratory threshold of −log_10_P = 4 are shown in black, whilst those significant at the Bonferroni threshold, calculated as −log_10_(*α*/number of markers) where *α* = 0.05, are shown in red.

Next, genetic analysis of the 33 DUS characteristics investigated via GWAS identified 58 genetic loci across 18 DUS traits using an exploratory significance threshold of −log_10_P = 4. Of these, 35 were significant using the Bonferroni-corrected threshold. Genetic loci were identified across all but 4 of the 21 wheat chromosomes (1D, 4D, 5D, and 7D), with the majority located on the B (31 hits) sub-genome, followed by the A (20 hits) and the D (7 hits) sub-genomes. Manhattan plots illustrating GWAS results for each DUS characteristic returning significant hits are shown in **Figure 3**, their distribution across the wheat genome is shown in **Figure 4**, and further details are provided in **Supplementary Table 5**. The 18 DUS characteristics returning significant GWAS hits had a mean heritability of *h*^2^ = 0.26 and included 15 of the 30 characteristics currently assessed under UPOV guidelines, as well as two former UPOV DUS characteristics that are no longer in active use. Of the 18 DUS characteristics with significant GWAS hits, the number of genetic loci identified per trait ranged from 1 (P1, P6, P21, P28, P31, P45, P46) to 12 (P80 ‘Seasonal type’ and P100 ‘Consensus seasonal type’, which have almost identical hits) (mean = 3.2, median = 2.0). Of the 15 DUS characteristics for which no significant GWAS hits were identified (**Supplementary Figure 3**), one had data for 104 varieties (P10 ‘Flag leaf – attitude’), suggesting low power to detect genetic loci, whilst the remaining 14 characteristics (P2, P8, P9, P18, P27, P30, P33, P41, P42, P43, P52, P55, P56, P68) showed lower mean heritability (*h*^2^ = 0.19) than those for which GWAS hits were identified (*h*^2^ = 0.26).

Twenty-five of the 58 GWAS hits identified at the exploratory significance threshold were consistent with the chromosomal locations of previously identified genetic loci, candidate genes, or map-based cloned genes (further details listed in **Supplementary Table 5**). The highest number of known genetic loci was associated with DUS characteristic P80 (Seasonal type), for which GWAS hits were identified at genomic loci containing the major vernalisation response genes *VRN-A1* (Fu et al., 2005), *VRN-B1* (Díaz et al., 2012), and *VRN-B3* (Yan et al., 2006), as well as four flowering time quantitative trait loci (QTL) identified in UK wheat germplasm (*QFt.niab.2D.02*, *QFt.niab.3A.01*, *QFt.niab.4B.01*, and *QFt.niab.6D.01*; Fourquet et al., 2024). DUS characteristics related to the levels of glaucosity observed on different plant tissues identified GWAS hits consistent with several previously described genetic loci: the *W1* locus (Hen-Avivi et al., 2016) identified for P14 (Flag leaf – glaucosity of sheath), P15 (Flag leaf – glaucosity of leaf blade), P16 (Culm – glaucosity of neck), and P17 (Ear – glaucosity); the *W2* locus (Tsunewaki and Ebana, 1999) identified for P17; the *W4* locus (Nishijima et al., 2018) identified for P14; and a bread wheat locus in an orthologous position to the wild emmer wheat *Iw3* glaucosity locus (Wang et al., 2014b), identified for P14 and P16. Finally, the positions of previously reported genetic loci were consistent with our GWAS analysis of DUS characteristics P1 (Coleoptile – anthocyanin colouration) (*Rc*; Ahmed et al., 2006), P22 (‘Straw – pith in cross section between ear and upper node’) (*Qss.msub-3BL*; Cook et al., 2004), P51 (Lower glume – external surface roughness) (*Hg1*; Hu and Zuo, 2022), and P79 (Grain – colouration with phenol) (*Ppo1* and *Ppo2*; He et al., 2007).

### Further analysis of straw pith thickness

**Figure 5:**
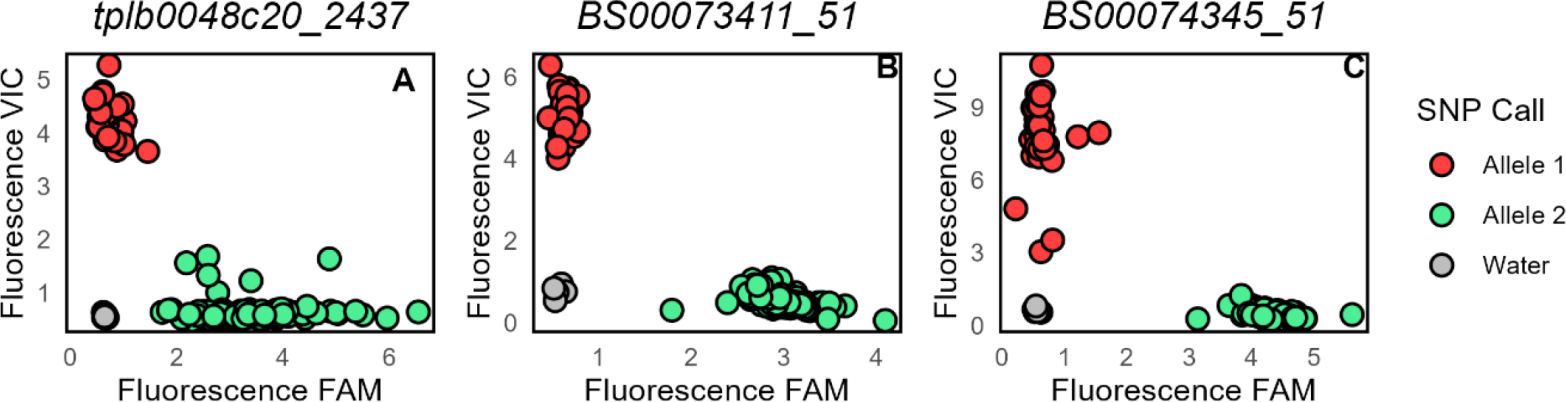
Validation of kompetitive allele-specific PCR (KASP) molecular markers at the *P22_3B821* locus for DUS characteristic ‘Straw – pith in cross section between ear and upper node’. Three selected single-nucleotide polymorphisms (SNPs) were converted from the 90k SNP array and genotyped across a panel of 82 varieties from the ‘WAGTAIL’ wheat association mapping panel: **(A)** *tplb0048c20_2437*, **(B)** *BS00073411_51*, and **(C)** *BS00074345_51*. Fluorescence intensity for VIC (blue) and FAM (red) fluorophores used in the assay to tag the reference versus alternative allele is indicated. For all three markers, allele 1 is associated with ‘Straw – pith in cross section’ score of 3, whilst allele 2 is associated with a pith score of 1 or 2. Data for negative water controls are plotted in white.

For 11 DUS characteristics, one or more GWAS hits were detected above the more stringent Bonferroni-corrected *p* = 0.05 significance threshold (**Supplementary Table 5**), of which eight GWAS hits were significant at −log_10_P > 10. The most significant of these was for ‘Straw – pith in cross section’ (P22) (−log_10_P = 35.72), for which genetic locus *P22_3B_821* was located on the long arm of chromosome 3B between 818 and 830 Mb in the reference wheat genome assembly (RefSeq v1.0; IWGSC et al., 2018) and in agreement with the location of the previously identified straw pith QTL *Qss.msub-3BL* (based on genetic markers *gwm247*, *gwm340*, *gwm547*, and *barc77*; Cook et al., 2004). Using the 90k array genotypic data, we determined haploblocks across chromosome 3B (termed haploblock-1 to haploblock-126). The two most significantly associated SNPs identified via GWAS were located within haploblock-122 and haploblock-126 (**Supplementary Figure 4A**). Haploblock-122 contained seven main haplotypes, of which three were present at frequencies above 5% (hap-122-a, hap-122-c, hap-122-d, and hap-122-g), whilst haploblock-126 contained four main haplotypes, all of which had frequencies of >5% (hap-126.a to hap-126.d) (**Supplementary Figure 4B**). All 28 varieties in the GWAS panel recorded as possessing the thick pith phenotype belonged to haplotypes hap-122.g and hap-126.d, whilst all but two varieties recorded as possessing a thin or intermediate phenotype belonged to the remaining haplotypes at these two haploblocks. To develop genetic markers diagnostic of hap122.g and hap-126.d, three SNPs on the 90k genotyping array that span the region between haploblocks 122 and 126 were selected for conversion to the KASP genotyping platform: *tplb0048c20_2437*, *BS00073411_51*, and *BS00074345_51* (primers detailed in **Supplementary Table 3**). Genotyping a subset of >80 cultivars from the ‘WAGTAIL’ association mapping population found them to replicate the allele calls derived from the same markers on the 90k SNP array (**Figure 5**, **Supplementary Table 6**), thus confirming successful conversion to the KASP platform. Notably, the most significant SNP in haploblock-126 (SNP *BS00073411_51*), located at 821 Mb within gene model *TraesCS3B02G594500*, lies just 7 Mb away from the wheat orthologue (*TraesCS3B02G608800*, *e*-value = 0, percent identity >98%) of *T. turgidum* subsp. *durum* gene *TdDof*, for which increased CNV has recently been shown to underlie increased stem pith thickness in durum wheat (Nilsen et al., 2022).

**Figure 6:**
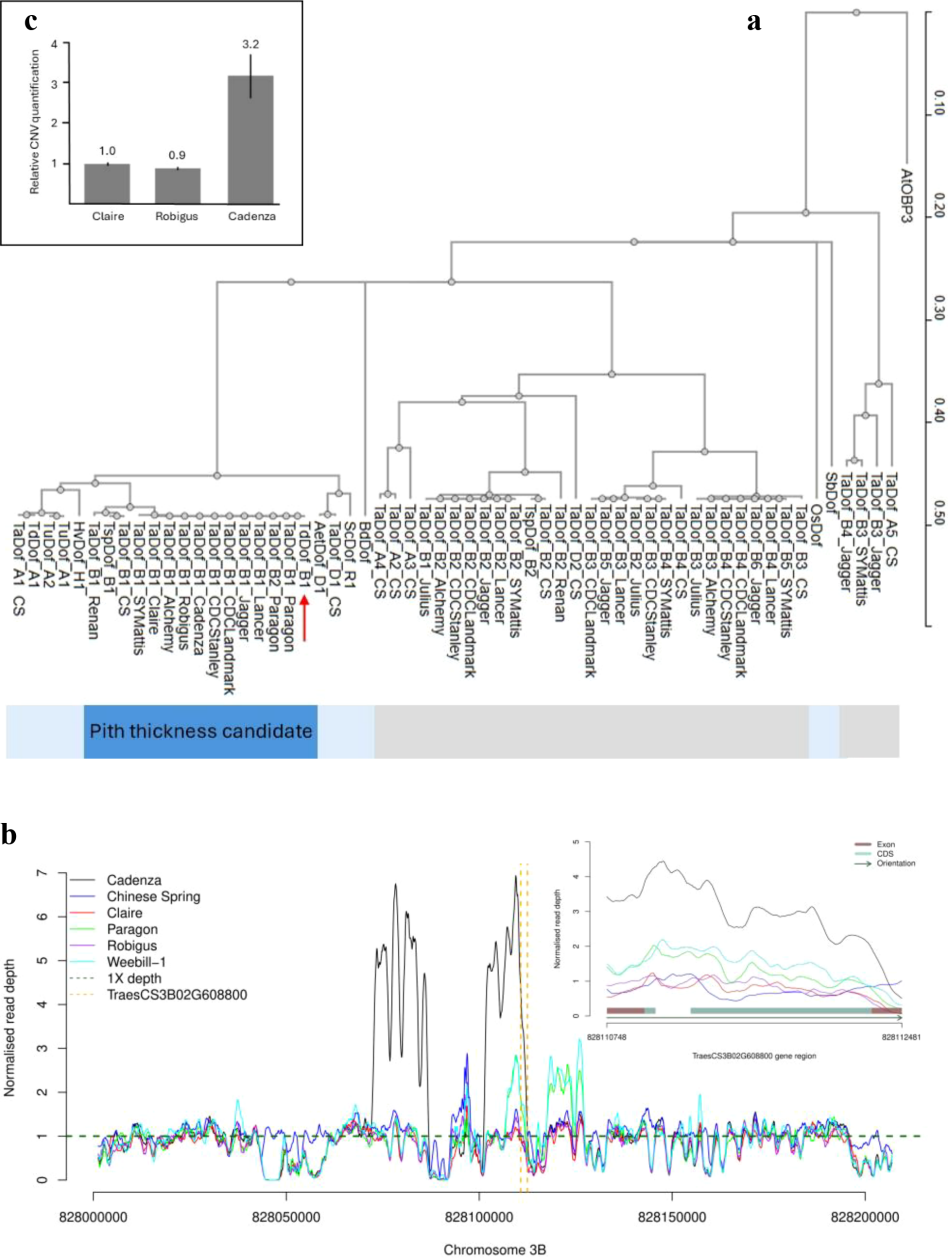
Evidence for copy number variation (CNV) for the bread wheat orthologue (*TaDof-B*, cv. ‘Chinese Spring’ gene model *TraesCS3B02G608800*) of the durum wheat gene *TdDof*. **(A)** Phylogram of Dof predicted proteins in bread wheat (*Triticum aestivum*) and other Poaceae species, rooted using the closest *Arabidopsis* homologue (OBP3). The Poaceae predicted proteins most similar to the product of *TdDof*, for which copy number variation (CNV 1 versus CNV 3) has previously been shown to underlie variation in stem pith thickness in durum wheat, are indicated by the dark blue bar. The light blue bar indicates the predicted proteins encoded by orthologous genes from diploid Poaceae species or from the homoeologous chromosomes on the A, B, or D sub-genomes in related diploid or polyploid wheat species. The predicted proteins encoded by paralogous wheat genes duplicated nearby on chromosome 3B are indicated by the grey bar. Bread wheat varieties are indicated (CS = ‘Chinese Spring’); included are those with available genome assemblies and for which the stem pith phenotype was sourced. TdDof is indicated with the red arrow. Aet (*Aegilops tauschii*), At (*Arabidopsis thaliana*), Bd (*Brachypodium distachyon*), Hv (*Hordeum vulgare*), Os (*Oryza sativa*), Sc (*Secale cereale*), Sb (*Sorghum bicolor*), Ta (*Triticum aestivum*), Td (*Triticum turgidum* subsp. *durum*), Tsp (*Triticum* sp*elta*), Tu (*Triticum uratu*). Note: Wheat B sub-genome paralogues closely linked to *TaDof-B1* were only included in the analysis for those cultivars with chromosome-level genome assemblies. Similarly, closely linked wheat A and D sub-genome paralogues were only included for cv. ‘Chinese Spring’ (the wheat reference genome). **(B)** Evidence of *TaDof-B* CNV via analysis of Illumina paired-end sequence depth in six cultivars relative to the wheat reference genome of cv. ‘Chinese Spring’ (RefSeq v1.0, IWGSC et al., 2018), indicating CNV = 1 in ‘Chinese Spring’, ‘Claire’, and ‘Robigus’; CNV = 2 in ‘Paragon’ and ‘Weebill1’; and CNV = 3 in ‘Cadenza’’. **(C)** TaqMan™ real-time CNV molecular assay, confirming CNV = 1 (‘Claire’ and ‘Robigus’) and CNV = 3 (‘Cadenza’). Error bars show +/− one standard deviation.

To investigate whether the CNV in the bread wheat *TaDof-B* gene might play an analogous role in controlling pith thickness, we first identified orthologous genes in related species (**Supplementary Table 7**), as well as closely linked paralogous genes in the genome assemblies of 13 wheat varieties for which stem pith phenotype was available or sourced (**Supplementary Table 8**; **Figure 6A**). The wheat reference genome assembly of cultivar ‘Chinese Spring’ contained one copy of *TaDof-B*, as did all the other cultivars with the exception of ‘Paragon’, which contained two. As some of the genome assemblies analysed were based predominantly on short-read data, we next analysed the Illumina 150-bp paired-end sequence read depth datasets available for five varieties with scaffold-level genome assemblies (‘Cadenza’, ‘Claire’, ‘Paragon’, ‘Robigus’, and ‘Weebill1’) as well as from the reference genome of ‘Chinese Spring’ as a control. Read depth analysis predicted *TaDof-B* copy number to be one in ‘Chinese Spring’, consistent with the one *TaDof-B* gene model annotated in the corresponding reference genome assembly (RefSeq v1.0, **Figure 6B**). However, equivalent analysis in the five additional varieties indicated whilst only one *TaDof-B* copy was annotated in their assemblies, read-depth analysis found CNV to be present at and around *TraesCS3B02G608800*, with estimates of CNV = 1 in ‘Claire’ and ‘Robigus’ (which have pith DUS scores of 1 = absent/very thin), CNV = 2 in ‘Paragon’ and ‘Weebill1’ (score 1 = thin pith), and CNV = 3 in ‘Cadenza’ (score 3 = very thick/filled pith) (**Figure 6B**; **Supplementary Table 8**). To further validate the putative CNV, we then analysed the recently released long-read genome assemblies for both ‘Paragon’ (Ensembl assembly GCA949126075v1, pith thickness = 1) and ‘Chinese Spring’ (RefSeq v2.1, Zhu et al., 2021, pith thickness = 1), as these were more likely to accurately assemble any CNV that had been collapsed in the scaffold-level genome assemblies. As predicted from our Illumina read-depth analysis, the long-read ‘Paragon’ genome assembly had two annotated *TaDof-B* genes sharing 100% sequence identity across their genomic sequences (*TraesPARA_EIv1.0_0898590* and *TraesPARA_EIv1.0_0898610*, termed here *TaDof-B1* and *TaDof-B2*, respectively). Extremely high DNA sequence conservation extended to 4.1 and 17.9 kb upstream and downstream of each gene (see **Supplementary Figure 5**), whilst in the long-read assembly of ‘Chinese Spring’, a single gene was annotated (*TraesCS3B03G1505000*, *e-*value = 0, percent identity >98%). Whilst not present in our GWAS panel, analysis of the additional eight wheat varieties which possessed chromosome-level reference genomes and for which we were able to obtain phenotypic data found all to carry a single *TaDof-B* orthologue (*e-*value = 0, percent identity >98%) and to possess thin (‘Alchemy’, ‘CDC Stanley’, ‘Jagger’, ‘Julius’, ‘LongReach Lancer’, ‘Renan’, ‘SY Mattis’) or medium (‘CDC Landmark’) stem pith thickness (**Supplementary Table 8**). Development of a quantitative PCR (qPCR) TaqMan™ assay confirmed Cadenza possessed three *TaDof-B* copies, whilst ‘Claire’ and ‘Robigus’ contained one copy (**Figure 6C**). Collectively, our analyses find the GWAS locus *P22_3B_821* to span a genomic interval containing an orthologue of the durum wheat gene *TdDof-B*, with evidence that as in durum wheat, a CNV of 3 is associated in hexaploid bread wheat with the DUS state referred to as ‘very thick/filled pith’, whilst CNV of 1–2 is associated with ‘thin/very thin pith’.

## Discussion

### DUS phenotypes

Current breeding efforts focus to a large extent on grain yield. For any DUS traits correlated with yield or other agronomically important traits (for example, those assessed to determine the UK ‘Recommended List’ such as resistance to yellow rust disease), genetic improvement in agronomic trait performance will lead to a reduction of DUS characteristic combinatorial space. The observation that more than half of the 33 wheat DUS characteristics analysed had a genetic correlation with yield < |0.2| (**Supplementary Table 4**) mirrors similar findings reported by Yang et al. (2021) in spring barley—where the most notable was the correlation between yield and barley ‘ear: shape’ and ‘ear: length’. Indeed, ear length has previously been found to show a positive correlation with the variety year of release in wheat (Spanic et al., 2024). Our finding that wheat ‘ear: shape’ was positively correlated with yield supports the assumption that ear shape traits directly affect yield in cereal crops. The negative association between ‘Ear density’ and both yield and cultivar year of release (i.e., a laxer ear density is associated with higher yield and with more recent varieties) (**Figure 2**) further supports the association between ear characters and yield—with release date previously having been shown to be a good proxy for yield in both wheat and barley (Sharma et al., 2022). The notably strong negative correlations observed between wheat ‘ear: density’ versus grain test weight and Hagberg falling number found here support the hypothesis that reduced ear density may be under breeder selection, and that this has a beneficial effect on grain quality. The finding that straw pith thickness had a negative correlation with grain yield could potentially be due to resource allocation trade-offs between source and sink tissues. It should be noted that the analyses interpreted here provide only a partial picture of the many traits and trade-offs between them that culminate to determine final grain yield at the end of the crop life cycle. For instance, just because a wheat ear is lax does not mean that it contains less grain compared to a denser ear in which spikelets are arranged closer to each other on the rachis—a lax ear could contain more spikelets, more fertile florets, or a combination of both.

For characteristics to be of use for their intended purpose in DUS examination, variation must be present in the breeding pool. The traits P25 ‘ear – colour’ and P69 ‘grain – colour’ were the only currently used DUS characteristics for which lack of phenotypic variation prevented GWAS analysis. For P25 ‘ear – colour’, the two varieties scored as ‘coloured’ for this trait (‘Maris Halberd’ and ‘Highbury’) were both spring type and were amongst the earliest varieties released in the UK under the UPOV system. Inspection of DUS descriptors for over 400 more recent UK winter and spring varieties found only three additional instances of the ‘coloured’ character trait (winter cvs. ‘Belenus’, ‘Gandalf’, and ‘RGT Anelle’). Assuming that ear colour does not have a negative pleiotropic impact on yield or other important agronomic traits, this indicates that i) ‘ear – colour’ could be specifically targeted as a trait if DUS-based differentiation between new variety submissions ever becomes limited in the diversity space, and ii) given that ‘ear – colour’ is a two-state characteristic and that colour traits in other DUS characteristics analysed here and elsewhere is genetically tractable (e.g., Cockram et al., 2010), genetic investigation via for example bi-parental populations may allow diagnostic genetic markers to be rapidly developed.

### Genetic architecture of wheat DUS traits

Power analyses using simulated traits indicated that the association mapping panel was suitable for the identification of genetic loci controlling traits of varying heritability (**Supplementary Figure 2**). Subsequently, genetic analysis of the 33 targeted DUS traits identified marker–trait associations for 18 DUS characteristics (**Table 1**; **Figure 3**), indicating that the majority are genetically tractable and included all but two of the currently used morphological DUS characteristics (‘ear – colour’ and ‘grain – colour’) and the three protein electrophoresis characteristics that assay for alleles at *Glu-A1* (P821), *Glu-B1* (P82), and *Glu-D1* (P83). GWAS hits were not evenly spread across the wheat genome. The higher number of GWAS hits on the A and B wheat sub-genomes is as expected based on the lower genetic diversity present on the D sub-genome due to the recent speciation of wheat via spontaneous hybridisation between progenitor AB and D genome wheat species approximately 10,000 years ago in the region surrounding the Capsian Sea (Gaurav et al., 2022; Wang et al., 2013). Genetic analysis identified several QTL clusters (**Figure 4**). Some of these were due to the presence of one or more major-effect genetic loci controlling related DUS characteristics. For example, the *W1* locus on chromosome 2B that controls epidermal glaucosity and which contains a β-diketone biosynthesis gene cluster orthologous to the *cer*-*cqu* gene cluster in barley (Hen-Avivi et al., 2016) is located within ~50 Mb of the major flowering time locus *PPD-B1* (Díaz et al., 2012), likely explaining the clustering of GWAS hits for DUS characteristics related to glaucosity (*P14_2B_047*, *P15_2B_005*, *P16_2B_047*, *P17_2B_005*) and flowering time (*P8_2B_020*), respectively. Whilst the DUS characteristics associated with protein electrophoresis of gliadin grain storage proteins did not have sufficient numbers of varieties with historic phenotypic data to conduct GWAS here (each, *n* = 83) and are not included in current DUS testing protocols, due to importance of gliadin composition for end use quality, it is of interest to note that we found GWAS hits for other DUS characteristics relatively close to the homoeologous *Gliadin* (*Gli*) loci located on the group 1 chromosomes. On chromosome 1A, we found the genes underlying the *Gli-A1* locus (e.g., *ll908* located at 5.0 Mb in RefSeq v2.1; Hu and Zuo, 2022) to be closely linked to the GWAS hit for ‘Lower glume – external surface roughness’ (*P51_1A_002*, −log_10_P = 19.82), which is controlled by the *Hg1* locus. The candidate gene for *Hg1*, termed *TaELD1-1A* (Hu et al., 2024), is located on chromosome 1A at 1.8 Mb in RefSeq v2.1, within ~ 3 Mb of the *Gli-A1* genes. On chromosome 1B, the *Gli-B1* locus encoded by the tandemly duplicated *Gli-1–201* genes (*TraesCS1B02G010400*, *TraesCS1B02G010500*, *TraesCS1B02G010600*, *TraesCS1B02G011000*) is ~ 6 Mb from our GWAS hits for three glaucosity-related traits on chromosome 1B at ~10 Mb (P14 ‘Fag leaf – glaucosity of sheath’, *P14_1B010*; P16 ‘Culm – glaucosity of neck’, *P16_1B010*; P17 ‘Ear – glaucosity’, *P17_1B010*). Identification of this locus controlling glaucosity was likely due to allelic variation at the *lw3* glaucosity repressor that originated from a wild tetraploid wheat species (Dubcovsky et al., 1997).

For those characteristics analysed for which no significant GWAS hits were identified (**Table 1**), including 10 characteristics in current use, this was likely due to low heritability, lack of power due to lower phenotypic data fill, increased genetic complexity, or a combination of these factors. Three characteristics returned marker–trait associations marginally below the exploratory significance threshold applied (−log_10_P = 4), indicating that the inclusion of more complete phenotypic data may result in significant GWAS hits (e.g., P8 ‘Time of ear emergence’, P9 ‘Flag leaf – anthocyanin colouration of auricles’, P55 ‘Lowest lemma – beak shape’).

### Wheat straw pith thickness may be associated with *TaDof-B* copy number variation

The highly significant GWAS hit for straw pith thickness on chromosome 3B (*P22_3B821*, −log_10_P = 35.72; **Figure 3H**) co-located with a previously identified straw pith QTL in this region (Cook et al., 2004). Notably, straw pith thickness in tetraploid durum wheat is controlled by copy number variation at *TdDof-B*, whereby increased copies of the gene are associated with the thick pith phenotype (Nilsen et al., 2022). Our finding that the straw pith thickness GWAS hit *P22_3B821* was located at a colinear chromosomal position in bread wheat indicates that variation at orthologous genes may underpin straw pith thickness in tetraploid and hexaploid wheat. Indeed, our analysis of *TaDof-*B identified in publicly available wheat genome assemblies for which associated stem pith thickness data were available found increased copies of the gene to be associated with thicker pith, indicating CNV may underlie the control of this trait in both wheat species. The 100% nucleotide identity observed for duplicated copies of *TaDof-B* in the more recent wheat genome assemblies which incorporate higher levels of long-read sequence data (cv. ‘Paragon’) explains why *TaDof-B* CNV was evident only via analysis of Illumina sequence read depth in cultivars with older assemblies that lack such levels of long-read data (including cv. ‘Cadenza’, CNV = 3, thick pith phenotype) (**Figure 6**). The recent findings that CNV represents a rich source of phenotypic variation in both animal (Celus et al., 2025) and plant species (Silaiyiman et al., 2025) highlights the utility of the generation of high-quality genome assemblies that can accurately resolve CNV to help advance crop breeding. Interestingly, analysis of a bi-parental population generated between cultivars ‘Cadenza’ and ‘Avalon’ (which our DUS data show has thin pith phenotype) previously identified QTLs for a suite of increased stem strength traits (stem strength, internode diameter, internode wall width, internode material strength) originating from ‘Cadenza’ (Piñera-Chavez et al., 2020) at the chromosomal regions spanning our GWAS hit. Our finding that ‘Avalon’ possesses a haplotype profile across the *P22_3B821* locus associated with thin pith and *TaDof-B* CNV = 1 indicates that the stem strength traits observed in the ‘Avalon’ × ‘Cadenza’ population by Piñera-Chavez et al. (2020) may be due to CNV at *TaDof-B*. Notably, a recent investigation of the genetic control of the chromosome 3B pith thickness identified the vacuolar processing enzyme encoded by *TaVPE3cB* as the most likely candidate gene (Liu et al., 2023). Liu et al. (2023) ruled out *TaDof-B* due to a lack of sequence variants in the RNA-seq data they used, and did not consider whether CNV might highlight additional candidate gene(s) within their target interval. Our GWAS analysis included an SNP within *TaVPE3cB* (*BobWhite_c11000_1049a*), which we found not to be significantly associated with the trait and to be located just outside of the interval that contained all of the significant GWAS hits for locus *P22_3B821*. Whilst this in itself does not conclusively rule out *TaVPE4cB*, the results we report here certainly indicate that *TaDof-B* is a strong candidate gene—especially given the analogous role of *TdDof-B* in the related crop, durum wheat. Our finding that *TaDof-B* CNV may underlie functional variation at the bread wheat chromosome 3B pith thickness locus provides that basis from which further analysis can be undertaken to clarify this hypothesis. Indeed, the TaqMan™ assay for *TaDof-B* CNV, along with the KASP markers developed here, will allow contrasting haplotypes/alleles at the chromosome 3B pith thickness locus to be tracked in future research and development activities.

### Limitations and caveats

Here, we summarise some of the caveats relevant to this study. First, we note potential limitations of the phenotypes in the DUS system itself—the robustness of which could be affected by environmental influence on phenotype (as indicated by low heritabilities in some instances), restriction in DUS characteristic combinatorial space (for example the very low phenotypic diversity observed here for ‘ear – colour’ and ‘grain – colour’), and the potential for subjectivity to affect characteristic scoring, with similar findings having been reported by others (e.g., Cockram et al., 2010; Yang et al., 2021). Secondly, due to the use of a historical phenotypic dataset and the large number of DUS characteristics available, the proportion of varieties with phenotypic and genotype data for a given DUS characteristic varied (mean = 292, median = 312) (**Table 1**). Whilst we thinned and quality-controlled the markers independently for each trait to help account for such differences, this data structure might still result in a minor effect on the interpretation of some of the analyses undertaken—such as the cross-comparison of results between the numerous DUS phenotypes. However, we believe that this would not impact in a meaningful way the overall findings and conclusions and is within the norms of practice and experimental error of such studies. Thirdly, whilst we used a mixed linear model as implemented in GAPIT, other methods are available for GWAS, such as BLINK (Huang et al., 2019) and FarmCPU (Liu et al., 2016). Some studies indicate that these outperform other methods (e.g., Cebeci et al., 2023; Sandhu et al., 2024), and here we provide all underlying datasets for others to explore such methodologies. Finally, insufficient phenotypic data were available to undertake GWAS for the three protein electrophoresis DUS characteristics currently used in UPOV protocols. Whilst variation at these loci is controlled by well-described allelic variation at well-studied major-effect genetic loci (*Glu-A1*, *Glu-B1*, *Glu-D1*), ideally, further work is required to formalise the link between the traits as measured within the DUS protocol with the germplasm studied here.

### Implications for breeders and variety testing offices

Overall, our results indicate that for the application of molecular approaches for wheat DUS traits, characteristics with relatively high heritability and controlled by simple genetics would be best suited for marker-assisted approaches, whilst those with more complex genetic architectures may be best suited to genomic prediction approaches. The relatively low heritabilities observed for some of the DUS characteristics analysed could be viewed as problematic for their intended use in DUS accreditation, i.e., the ability to uniquely describe a given variety based on its DUS description. Low heritability of DUS characteristics has also been reported in the related cereal crop, barley (Yang et al., 2021). However, DUS processes currently include protocols that mitigate against such variation, such as the use of ‘minimum distances’ in trait variation space required in order to call distinctness. Current UPOV guidelines provide two accepted application models for the use of molecular markers in DUS [UPOV document TGP/15/3 Guidance on the Use of Biochemical and Molecular Markers in the Examination of Distinctness, Uniformity and Stability (DUS), available online at https://www.upov.int/tgp/en). The first is the use of characteristic-specific molecular markers where the molecular markers provide a like-for-like replacement for the phenotypic characteristic. The second model is the use of molecular distances in combination with phenotypic distances to manage variety collections. In the strictest sense, for characteristic-specific molecular markers, this is only possible where diagnostic molecular markers have been identified that directly assay for the DNA change(s) that result in a state change in a DUS character. Whilst this should be possible for those characteristics with relatively high heritability and controlled by a small number of genetic loci (for example P1 ‘Coleoptile – anthocyanin colouration’ and P22 ‘Straw – pith in cross section’), for DUS characteristics with low heritability or more complex underlying genetics (such as P41 ‘Lower glume – beak length’ and P42 ‘Lower glume – beak tip’), like-for-like marker replacement is not currently a feasible goal. Rather, exploration of alternative methods for such DUS characteristics would be necessary—for example, the use of genome-wide genetic marker sets to predict phenotype following genomic prediction methodologies. As distinctness criteria in current DUS processes relies on exceeding a threshold of notes on the phenotypic scale for a given characteristic, this should be factored into the thresholding applied during genomic selection approaches—as recently explored in the related cereal crop barley (Wright et al., pers. comm.). Overall, our findings inform commercial, DUS regulatory and scientific advances in future crop research and development programmes.

## Data Availability

The phenotype data presented in this study is available via the **Supplementary Tables**. The genotypic data presented in this study is available via www.niab.com/resources under the dataset titled 'Wheat 90k SNP dataset'.
