# Supplementary material for: Association mapping of wheat distinctness, uniformity, and stability traits identifies evidence of TaDof-B copy number variation associated with stem pith thickness
Source: Front Plant Sci. 2026 Mar 31;17:1739489. doi: 10.3389/fpls.2026.1739489 (PMC13077854; doi:10.3389/fpls.2026.1739489)
Supplement: Supplementary file 1 [file DataSheet1.docx]

**
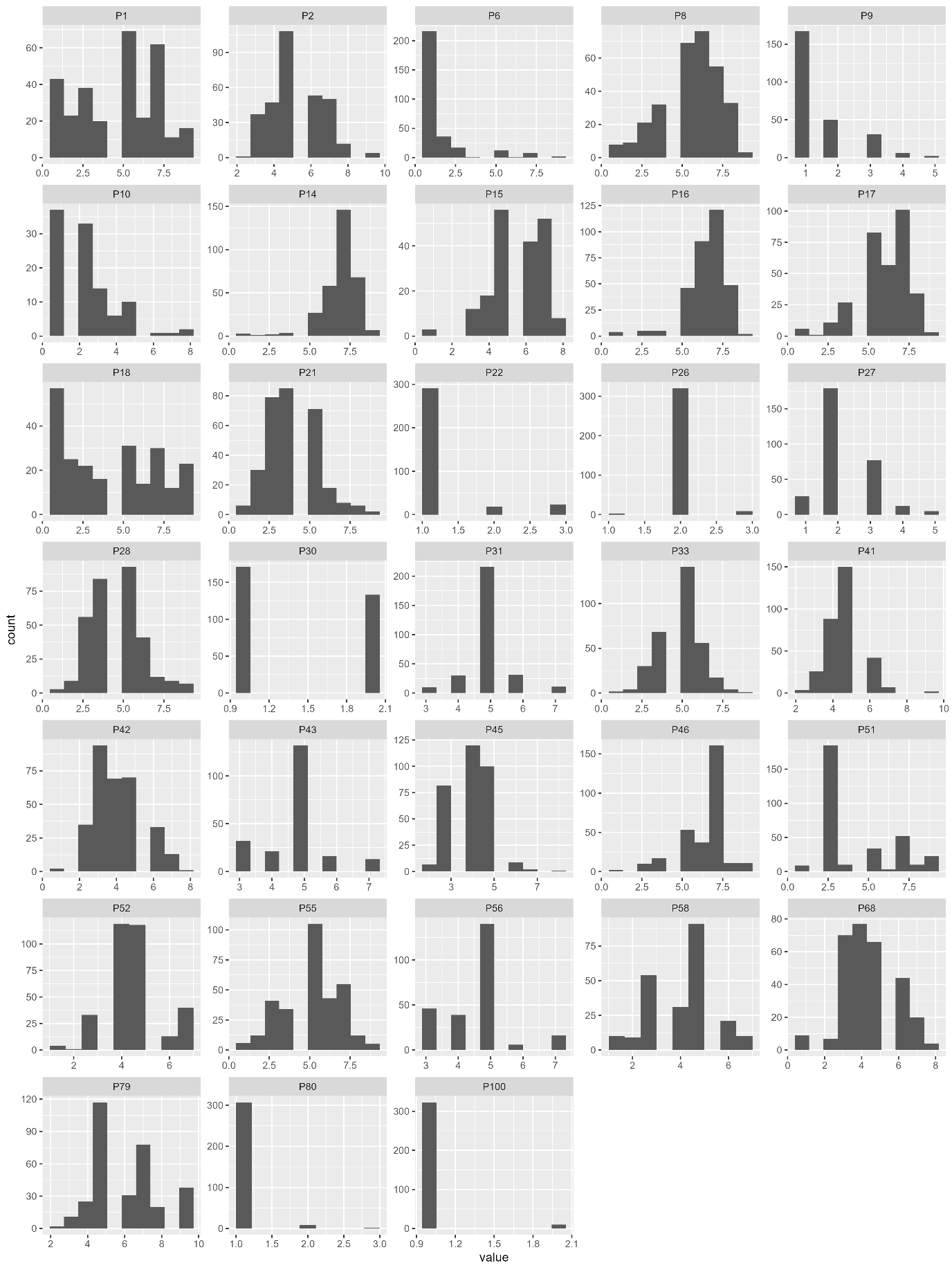
**

**Supplementary Figure 1.** Histograms of trait distribution scores for each of the 33 wheat Distinctness Uniformity and Stability (DUS) characteristics included in this study.


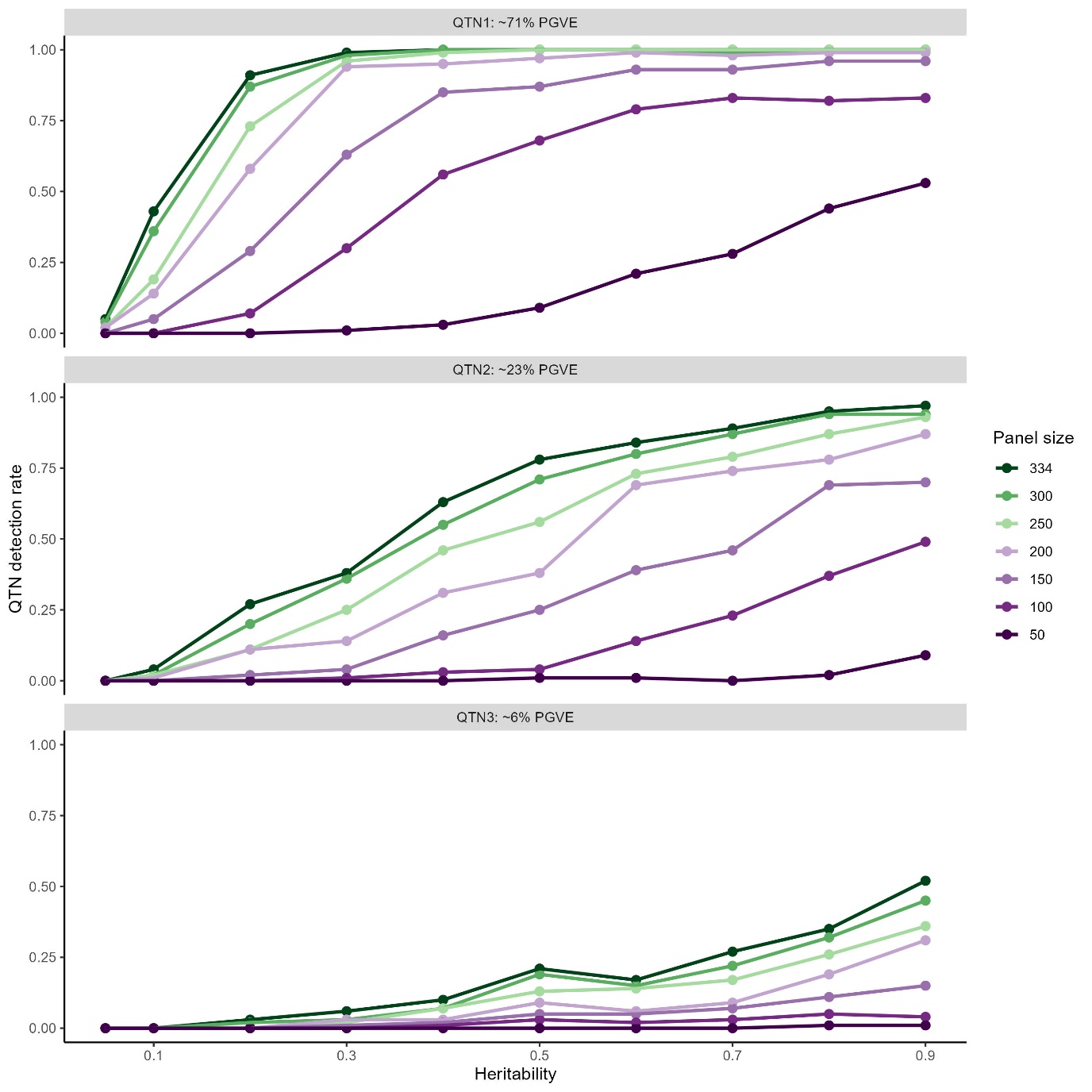


**Supplementary Figure 2.** Power analysis. Quantitative trait nucleotide (QTN) detection rate, the proportion of genome-wide association study (GWAS) runs where a significant single nucleotide polymorphism (SNP) was located within 20 Mb of the target QTN, from traits simulated at various heritability levels, where each datapoint is an average of 100 GWAS runs that were scored as successful or failed detections. Traits were simulated to be controlled by ten QTNs, where the top three QTNs with the greatest contribution to the genetic variance were analysed for their detection, explaining approximately 71%, 23% and 6% of the genetic variance (PGVE). Seven association mapping panel sizes were analysed by generating random subsets of the full panel.

**
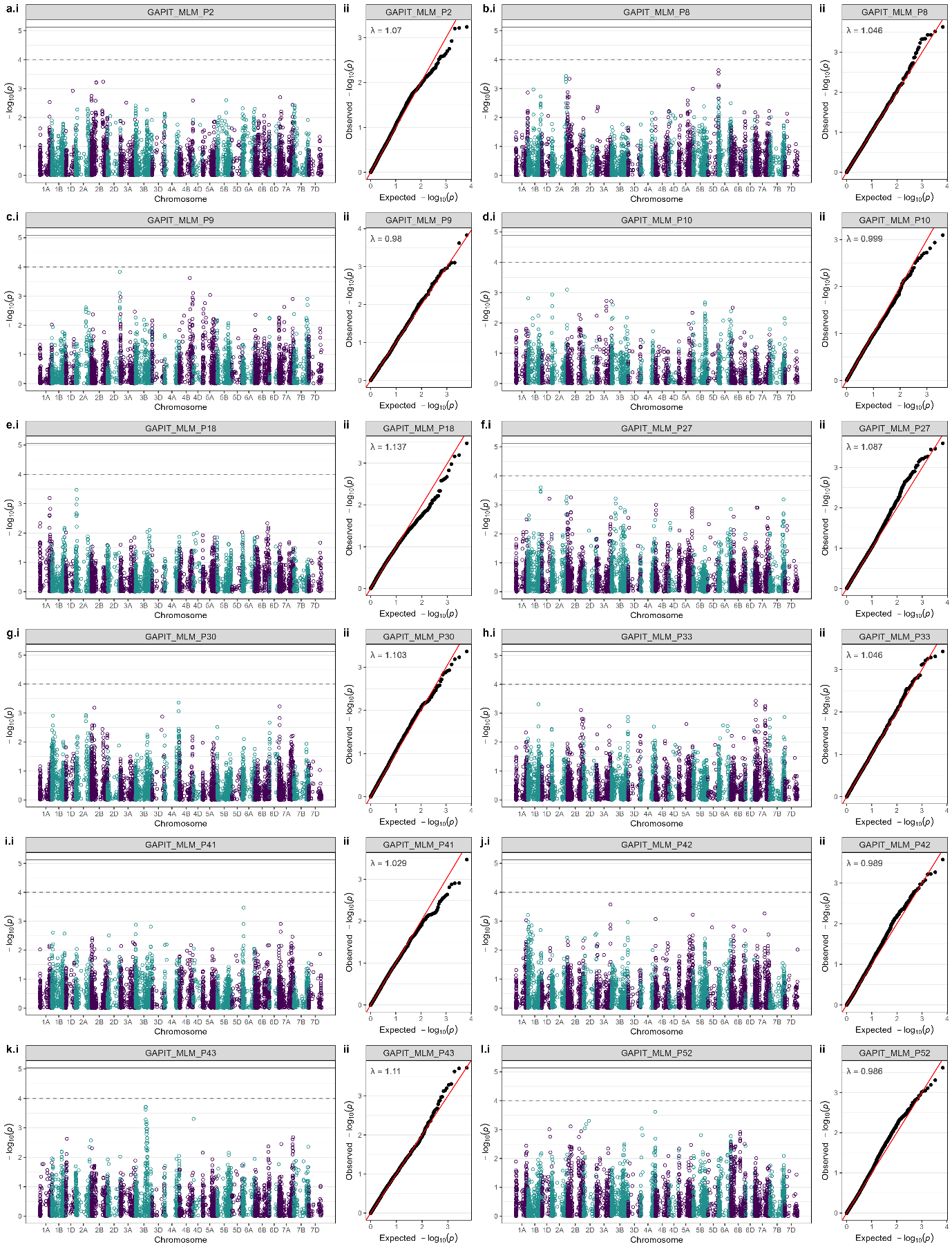
**

**
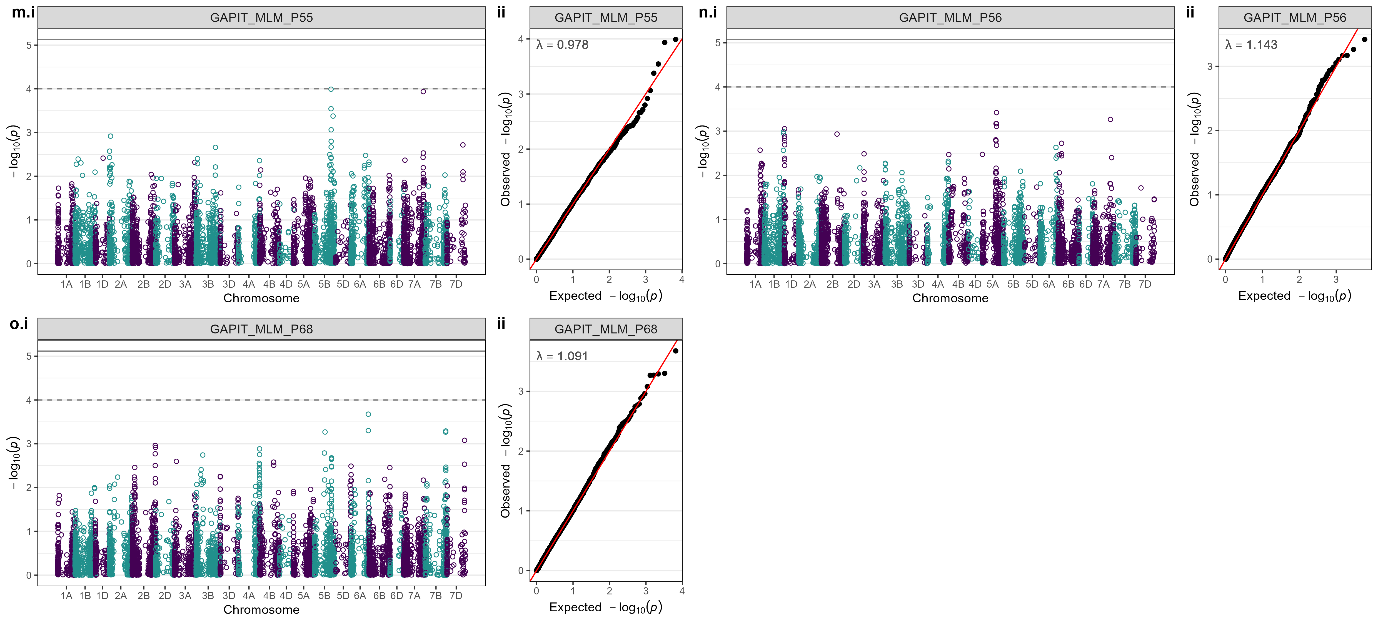
**

**Supplementary Figure 3.** Manhattan and quantile-quantile plots of the 15 Distinctness, Uniformity and Stability (DUS) characteristics for which no significant (-log_10_P < 4) genome wide association study (GWAS) hits were identified.

**a**
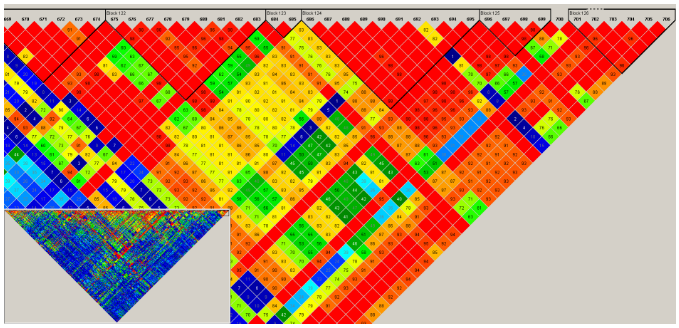


**b**

**Supplementary Figure 4.** Haploblocks on wheat chromosome 3B spanning the genetic locus *P22_3B821* identified via genome-wide association study (GWAS) of DUS characteristic P22 (‘Straw – pith in cross section between ear and upper node’). The two most significant single nucleotide polymorphisms (SNPs) were located in haploblock-122 (*tplb0048c20_2437*, SNP 683; -log_10_P = 35.7) and haploblock-126 (*BS00074345_51*, SNP 704; -log_10_P = 34.2), with the difference in significance due to SNP call at one variety (W1088_ROSETTE). (a) Haploblocks across the whole chromosome are shown inset, bottom left corner; superimposed over the zoomed detail of the haploblocks at the end of chromosome 3B. (b) Haplotypes, numbered .a to .g, present in chromosome 3B haploblocks 121 to 126. The proportional occurrence of each haplotype within its haploblock is indicated in grey. Haplotypes identified as adjacent are linked by lines, with the thickness of the connecting lines indicative of frequency. The haplotypes identified as most common in thick pithed wheat cultivars are highlighted in bold. Chromosome 3B SNPs belonging to each haploblock are indicated at the start and end of each haploblock, and elsewhere as necessary. SNPs *tplb0048c20_2437* and *BS00074345_51* are represented by SNP numbers 683 and 704, respectively, and are highlighted in blue.

**a**


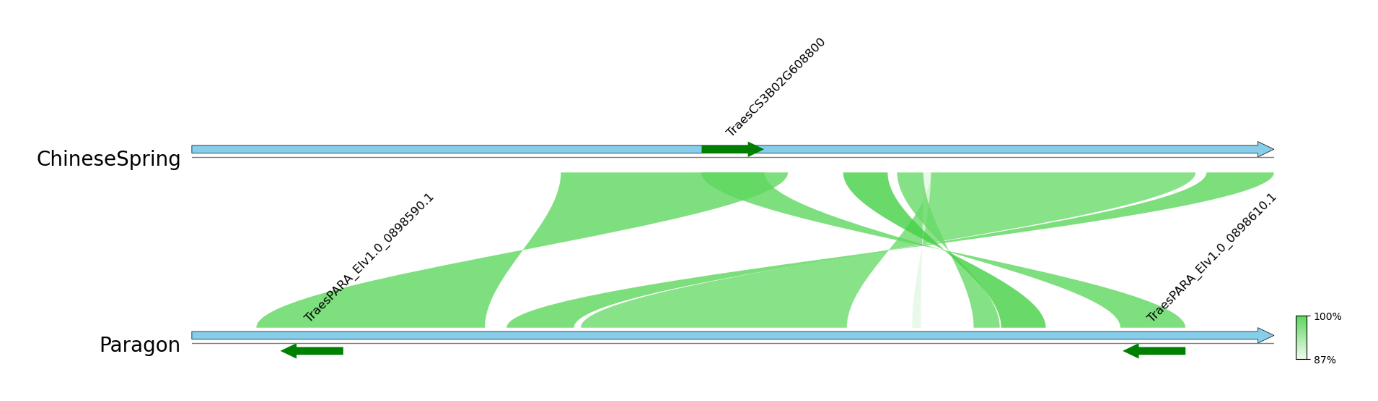


**b**

>gParagaon region 3B dna: primary_assembly:GCA949126075v1:3B:9079519:9152689**:**1, reverse compliment. First gene model: TaDof-B2 TraesPARA_EIv1.0_0898610. Second gene model: TaDof-B1 TraesPARA_EIv1.0_0898590.

GATTTCCTTCGGGACCAAATGTACCAAATCCTGCTTTGAGTGGAGATCTGATCATTCCTATATTGTTAGATTTGACTTTCATCTATGTGTTGCTCCGCGTGTATCTCGGCATAAATGGTTCATCATACTATGATCTAATGTAGTGATTTTAATTATGTTACTTCTTGGTGAATATGCATTACATTGTATTTTCATGGAAGCCTTTTCTTTCCTCTCTAATCATACTACGGCTCTTATCCTTGCCTCTATATGAACCTAGAAATATTATGGCTGACATAGTGATACTCTCCCTCAGGGACATCCCGACACATGATGCTCCATCAACAACTTCAGAATATGATGCTGATCCTCAACCTGCAACTTTGTAGCCACACTGCCTTTTGTTTTATGTAGAAAATTAACAGATTTTGTTATGTGTTTCAGCATGAGCTAATAATTAGAAATAACAGATTTTGTTAGTTATGAGATTGGTCCTTCAATATGGCATAGAAAACATATTTTTGAGTACTCCTGCTGTTTTGCTGCTTGCAAAGTAGTTTGATGGTTTTTTAAACAATTCTAAGCACGTATTTATTGATTTTGTATCCGTTGGGATGCGTACGTATTTATTGATTTTGTATCCGTTGGGATGCACGGACCGTTTTGCTATACCTGCTGATAATGGGAGGTGATATTCTTCTCTTCCGTCATGTGCATAAGACGGATTTACCCTTCCACTAAATTACATAATTACATTCGTCGCCCGACATTCATATCTATGTCCAAGTCGTATTCTAGGAAACCAATCCTTCCATACATTCTCTCTCACAATTAGGACTCTTGATGGGTGTCGAATCTTCCCTAATCCCTCCCATAAATGCACCACCCCGGAGGAGGCGAAGAACAGCGGATGAGCCACTAGATGAAGCGACATAGGGGAGGGTCATAACAGCAAAAGGGAATTGGCATGCGTGGTTATCACGGAGGGTGAAGGCAACGGTGGATGCTCGTCAATCGAGTGAGTGGAGGGCATAATCAAGGTCTCTGTCCATGGCATCTACTACTTCATGCTGGCCAATCACCATGTGAGATCTCTTCTTACTATTCTTTGTTTGTTCCTTTTGTATATAAAATAAAAAATTGTTTCTTGGGTTTATAAAAACATTAAAATGAGTAAATGTTTGCTTCATTCTCGTCATGTTGCTTGATCAGTTGGGCAGCCGCTCTGGCATGAAAAACATTTGGTGTTATGTAGCAATGACCAACAGAGGTGAGACGCAATGTTGAAACGAAGGGCGTGCACTTACATGGGTTATACTAGGTGTGTGTGATATTTGTTTCTCCCATTGCAACATATAGTCATGTTTGTTTCTCCCGCACGCACTTACAACACATAGGCGTGCACTTACAGAAATTGTCTATTTAAACAAATGTCCGAAATATTGAAAACAAATGTTCGGGAAATCAAAAGTTCATGATTTTTTAATAAGTTCGTGTATTAAAAAATGTTTATGAAATTGAACAAAATTTTGCGAATTCAAAAAAAGTTCATGAATGGTAAAATATCCTAAAAATTCAAAAACTGATCGCGACTTACAAAAAAGTTTATTCATTCACAATATGTTTGCTGTTTCAAAAAATGTTCGTCCAATTAAAAAAGTTCATTAAATAAATAAAATTGTGAATATAAAAAATTATCCCACCAATTTTCAATAAAATGTCCATCAATTCTAGAAATGTTCCTCAAAAAATTGTTTAAAAAATGCTCACAATTTGAAAAAATGATTGCAAATATATAAAATGTTCATAAATTCAGAAAATGCTATTGATTTCAGAAAATATTTGCAAATTTGTATAAATGTTCATAAATTTGAAAAATGTTTATGATTTGAAATATGTGCATGAGTTTAAAAAAATATTCATGATTTCAAAAATTGTTAATTTTTTTTACTAAGTTGAGAGTGATAGTGTGTCTCGATGATGCAGCAAAATATGGTCATGCCTGTTGAAGATTAAGATTTATTTTTTCTCCCGTTGCAACGCACGGGCACTTTTGCTAGTAATAATAAAGCAAATTGGGTTTCTTTCGTCCGTCATGACATTTTTTTCAGAAAAGTCCCTCTATTTCAGATAATTCAACCCGCAGTCCCGTTTTAAGTTAAAACGAAGCGTTATTTTCGTATTTTACACAAAAGTCCCTGTCTTTTCTTGAAATCAACCCGCCATCCGGATTTAAGTCACACCCGAACCGTTATTTTACATTTTTCGAAACCCCCCCTGATGTTTTAGGTAATTCATCCGCGGATCATATTTAAGTCAAACAAATGCTTTTTAAAATCATCCATATCTTTTATACCGTAACTCCGATTTGAACATGTTATATATGAAATTTGATTAGAAAAATATGTAGAATATGAATATGAGATTATTTTTACCTGTTAAGTATTTTTAAATATTATTTCGGAATATATTTGAGTCAAACCAAATAATTTTCTAAATTATTTGTTTCTTTTAAACCGTAACTTCGATTTTAACATATTATATATGAAATTTTATTAGAAAAATGTGTGGAATCTAAATATGATGTTAATTTTACCTGTTAAATATTTTTAAAGTATTGTTATGGAAGCAAACTTATAATTTATAGCGCAAGATCCTTTTTTCATACCGGCGGCGATCCGGATTGCAAATAAATACCCATAATTTTCTAAATTATCTGTATCTTTTAAACCGTAACTTTGATTTTAACATATTATATATGAAATTTTATTAGAAAATTGTGTGGAATCTAAATATGATGTTAATTTTACTTGTTAAATATTTTTAAAATATTGTTATTGAAGCAAACTTATAATTTATAGCGCAAGATCCGTTTTTTTCATACCGGCGGCGATCCGGATTGCAAATAAATACCCCACTATAACCATATACGGAAAAGAAAACATCAATAACTACACGTGCATACCTCTGAAAATTGTTGCATGGGAAAACAACAGATTTCTCATCGCGAGAGTGAGAGAAAGCGAGCGAGAGAGAGAGGGGGGAGAGAGAGAGGGAGAGGGAGGATTGAGGGAGAGAGAGACGCCTAAGGATTAACCATACTCAACACACATTTTTTGTTGTTTTGTGTAAACACCGAGGCCATCGCCGACGAGGATGAGAAGGAGGATAAGCGGCAACACAAATATGATGCCTCGCAAAAATAAAGGAGATGGACATATTATGGTCTTGAGTGATAGTTGTTAGGTTAAGAGTGTGTGTTATGTTTTCTCTTCCATTGCAACGCACGGGCTCTTTTGCTAGTAATAATAAAGCAAATTGGATTTCGGCCGTCCGTCATGCACTTTTGCGATGCGAGAGCGATGTATAAATAGAGATGGAGGGAGTGGTTTATTATGATAACAGGAATCGACGCACGTAAATATGCTATGCACGACGTGATACCAATCAATCATCCTCCCCGCATATATGACCAGTATATCATCTCTTCTCTCGCAAACTGACAAATAAACCACAAGATATAAAAACAGTTATTGCTGCGAGGAAGAAAGGAGCGTGATGGTTGTTTTCTATGACTCCTGCAGGGTATACGATCGATGCAGCTAGGCACCGGTATTGGGATCTCATGATGTTGGATCCGGCATGGGTTGTACCGATACTTAAGGCCTGTTCGTCAATCCACCGCTCCCAGAAATACTAGAATCTAGGGAGCATCTCTTTCTCCACTCCGTATTTTCAACGGGCCAATAGCTCCTGTGCGACATGTTTGATGTGAATTTGCTTGAGTGCGACGTTGTTCGAGCGAATGGCTGTGGTGCGACACATTTAAGCAGGTAAATAGCCCAGGGCCACATGGGGTGTTAAATGTGGTTAAATTGGTCGTCAACCAAGCCCGTTTATGTTAGGACATGTGGGTCCAACTTAATTGCTCCTCAAAACAGCTGACCGTGCCCTGCCGCCCGACCGTGCCGGACCCGCCACTCTGTGCCCGCCCCCTCCCCCCTTCTTCTCCGGTGGCGGCGGATCTTGCGTTCTCAACGGCGGCGGCGGAGCCTTCGTCCTCAAAAAATGGTGAGTGAAAACCCTAGTCCCCCTCCCGTTCCTCTCTCGGCCCCGTAGATCTCAGCTCGTTTCCTCTGTTTTCGCTTGTTGAATCGATAGGTTGTGAGGGGAGCCCGTGGGCATACTGAGATGGAGCCCCCAGATTCAACTTCGAATAGGTAATGCTCCTCTCTCTGATCCAATTTTGCATGCAATTAGGGCCTAGTCTGCTCTTTCTCACGGGCTCACACTGTCCGCCACTGACAGAGGGGATGCTGCCGCTCGGACCTTCGAATTGACGGTCAATTTCTTTCCATCAAAGGCAAAATTAGTTGATGGTAGCATTCAGAACATTGAGAGAGACACAATTGTTAAATGGGAGGTTGAGTTTACAGAGATTGATCCACAAGTTCTACAGAACATGGGAGAGAAGGCAGTGAAGAAATGGGTGGAAAAAATTGGGGAGAATGTTGTTTGGGGTCCAGAGCAAGAAGTATCACTGTTGCGGTTTGATGATTGGAAAGGAGAGTATGTGAGAATGGAAGATGGTGAACAGATTGTTGATGAAATCGATCGGCAAAACGGCTGGACAAGCAAACGAGCTAATTTTTTTGCTGAGCTGGTTGATCTGAATATTTTTTCGAAGGTTGGATATGTGCCATCACAATTGGCTGCGCAGATGGTAGATGATGATTGGGCTACACAGAGGCCGTTGATTCCCATGTGTACTGAACTTACAGTAATAGCCGAAGAGGGACAGGTGACTGGAATTGAAACTGAAACTGCAGCAACTGTTGATTGGAATGTAGTTGAATTAGATGAGCCTACTGATTTGGTCATTGCACCAATGCCTGACATTGAGATGGCCAAACTTTTTGGCATTCCAGTCGATGACAGAGATAAGCAGGAGAGGGGAGAATCTAGTTTGCCTGCTAATGTTGATGAAGATGTAGATGGACAATTGATGGAACAAGCTGCAGATGAAGTAGATGATGCACATGATGATGAGCTGGTGCATGTGTATGACAAAGAAAACCCTGTCATTGAAGTAGGCAAGTTCTTCCCAAGCATGAAGGAGTTTAGGATGTGTTTCAAGACTTATGCAGTGAAACATGAGTTTGATGCCAAGACTGTTTGGACTGATAGAAAGAAGTTTTATGCGAGGTGCAGAGGATTTGATGGTAGTGTCAAGCCTTGCAAGTGGTACATATCTGCTAGACTGCAACCTGATGGAAGTACTGTCAGGGTTAACCAAATCCTCAATCAACATACTTGTATTACAAGTTCACAGAGAGTATCAACCATGACATCACAACTTTGGGTTGCAGAAAAGATCACCCCAATTTTAGCCAAAACACCAAACACTACTGCCAAGAAACTCAAAGTAGACTTGGAAAAGATGTACCCCATTAAACTGAAATATACCACAGTGTGGAAGGAAAAACAAAGGACAATGAAAAACTTATATGGTGATTGGGCAAATACATTTAGGATGCTTTACAACTTCAAAGCAGAGGTGGAAAAGAGGTCACCTGGTAGTGTTGTGGAGATAGATACAGAGGTATCAGCCAAAGGTGAAGTCAAGTTCTCCAAGTTTTTTATGGCTTTGAAGCCTTGCATAGATGGCTTCAAAGCAGGGTGCCGTCCATATTTGAGCATAGACTCATCATTTTTGACAGGCAAGTGGAATGGTCAGTTGGCAGCATGCAATGCTCTAGATGGACACAACTGGATGTTTCCTATTGCTGTTGGCTTGTTTCAGTCAGAAACAGAGGCTTCATGGACATGGTTCATGATCCAGTTGAAAAGATGCCTAGGGCCAGTGTCACCTTTGGCTATACACACAGATGCATGTAAGGGGCTTGAAAATTCAGTGAAAAGTGTTTTCCCACATGCTGAGCAGAGGGAGTGCTTCGGTCATTTGTGGATGAATTTGATCAAAAAATTTAGAGGAGAAGAATTTGGGCGCATGTGGCCAGCAGCAAGATCTTACACTAGACAGACACACAAATATCATCTTGATAAGATAATGGCAGCATGTGATGAGTTTGGTCCATGGCTGAACACCTACCATTCTTTGTTATGGTACAGGTCAGCATTCAACACTGCCATCAAGTGTGACCACATCAACAACAATTTGGCAGAGAGTTTCAACAATAAGGTGAAGGAGTTAAAAGATTTGCCTGTGCATGACATGGTTGACCAAATTAGGATCATGCTCATGCGGTTGTGGGAATTGAGAAGAAGGATAGGTGATTGTCTGCAAGGTGATAAGCTTCCAGCAGTGGTACAACAGGTGGTCAATAGGAGCAGAAGTCTTTCACATTTGTTTGTTGAAAAATCTTCACCTTGGGGTGCTGAAGTTAGAGATAACAAAACTGGAAGGAGACATGTTGTTAACACTGAATTGCATGATTGCACTTGCCTCGAGTGGCAACACACTGGTAAACAATGTGAGCATGCCATTCTTTTCTTAGCATCCCAACCGAAGATAAACATGCACCCATATCTGCATGAATATTATTCGGTAGCAAGATTCAAAGCTGCATATGCTACTCCAATTCCAGCACTTACAGATCAGTCTCAGTGGCTTGAAGTGGACATTGAATTTTCCATGTGTCCTCCCTTGATGAAAAGAAAGGCTGGCAGGCCTAAACAGAGTAGATTCAAGGCATGGTTTGAGAAAGGTGGGAGTAGTAAGAAGGGAAAGAAAGATGAAAAGCCAAAAAGGGCCCAAAAAGGTAACAAAAATAGATGCAAGTTGTGCCAGGAACTTGGGCACAGAGTGGGATCTATCAAATGCCGTTACACTCCTGATAAGCCAAAGTATGTTCTTGTTTATTTGTCTGTGTTTTGATTTGGTGCTTTTTCCCAATTAGTATATCTATAACATTTTCTGCACAGGAGGAAGCGAGCAAGTCAGCCCCTTGTTGTTGAACAGTGTTGGCCAACCAAAAAAGCAAGAGTCAATGGCGGTAGAAAGAAGAGAAGTGTGCCTGAGCCTGAGCAGACTGAAGAAAATCCTGCTGCAGTCAACATTCAGACTGAAGAAACTGATGTGGAGGTGCACACCGAAGAAACTGAATTTGAGCGTGTCGAGGTTCAGACTGAAGAAACTGAACCTGAGCGTGTCGAGGTTCAGACTGAAGAAACCCATGTTGAGGTACACACCAAAGAAACTGAAACTGTTGTCAACATTGAGACTGAAGACACTGATCACGAGGGTATTGGCGAGGTGTTGAAAAGACCAGTGAAGAAAACCAAGATGATCAGTGAACTTGTGTGTGTAGTAGAACCAAAAATAAGAAGGGCTAAGGCGAAGAAGGGCACACAACGTGGTAGGAAGAAGTAGAACAGAGAATAGTTTGAAAATTTGGGACATGTAATAATATTTGTAACTTGGTGCGTACTGGTAGTCACTATGTATCCCCATATTTCTATTCGAACTTGTTTGTCTAAACCGCCCGTCTAAACCAGCCAAATAAAATTCAAATTTAAATTTAAATTTGGGCTATTGATGTTTTAGTGCTATCTAAAGTACCTCAACTGAAATATGTTTGCTTGCTGACCATTCCGAGCCCTTTTGGTAAGTTGAACTCATAGTCATGAAAAGTGTGTTTAAAATGACCTCCAAAGGTAGGGTAAACGGCCTCATATTTAAGCAAGTTTTTTTGGCACCTTGTCTAAACCAGCCAAATAATTTTTCTACACATCTATTCTACCTATATAGTGTAAATCTAAAGTCTCACTATTTATTGAATTAATTTTCTATTTTTTCTTTTCTTTTTGAAAAAACAAGGTTTAATAGAAAATTATATATAAAACAAGTTTAAAACATGAAAATGAGAAAAGTAAGTTCAGATCTTTCTTAGTCAATCCAAAATGAAGTTTTGGTGAGGTTTTCACACTTTTCATTTTCAAAACTCCACCTACTCTCGGGTGCCCACTACTCTCTCCCTTCAACTCCATACTACATTGTTTGAGGAATAACAATTTTGTGAAGGATTTTTCGTAAAAATGTATGTAAACCATATTTATATTTTTTTCTCGTTACTATACGACATAATAAGACTATGTGCGCAAGTTTTATATTTTTTTTATTTTTTTTGAATTAGTTATGCTCAACCCTAATCAGTCAAAACCTCTCAAAACCCCTCAAAACCCCTCTCAAAACCCCTCAAAACCCCGCACACTACCTGGTCATCGATCGTTGTGCGTGGACGGTTGGGATCAGGCGCTAGGGTTAGCTACAGTGTGCAGCGATCCGCATGAGACGCGCTGATTGGTTGACGCGGTGGGGTTTGGATCAGCCTCTCTCGTTGGAGCATCAGGACCGTTCATTTGAATCCAACGGCAAGAGTTGACGCGGTGCATGGACCAAGCCTACGGAGTGCCCTTTCCATCGTTGCATGCGTGCCCGTGCCTACCCGCAGCATGCATGCCCACCCGCAGGACTGCGCCCGTGCGTTGCATGCATGCCTCGACGTAGCCCTGCTCCGCCACCCCTATCAACGCAGTAACCTGTCGCATGCCTACCATTAGAACCGATGCAGCGTCGCATCAAAGCATGCACCCGGCCACAATGCAGCAGCCCGATGAAGACAACCAAAAAGCCAACGCTGCATGGCATGGTGTGCACGCTGTGCATGGCGTGCTCCTCTTTGACGACGCAATACTGGCATGGTATCGATGGGGCCCGTATCGATGTGGGTATTGATGCAGTTCAAATGGCGTGTTGCCCGTTACAAGATGGGCAAAAGAAGGCGTACATTATTGTCGGTCACGTCTCCCATCGACCGAACCTTGCCCTCTAGCCAGGCCCTAGCAAGATGAGCAAATTAATGGGCAACGGCATGCATGCACGGGCGGCACATGCAGAGAACCCGGGGAAGGCGTGTCCCCTTGACCCACGACTGCCCGTCCCCCTTGACCCACGACCGGTGGCACAGAAGCGTAGCAGTCCGTTTCCCACGGCCACGCTCGTGTACATGCTTTCATGGGGCGCCACCAAACGCCGCCCGCCCATTAATTCTAGGGTTTCGACGGGGTGAAACGACGTCGCACTTGGGCTATTTAAGCCGGGCACTATCGTCCTCTCCCTCCTCATCCATCATACCCCATCCTCTGCCTCCTCTGCCCTTCTTCTTCATTCTTCTCCATCAAACCCGACCGAGCACTCGAGCCACCCCGCCACCATGCAGTACACCGCCCCCACCTACCGCTTCCCCCCAACCGTGCCGGAAAGGCTCTACCCGGCCGGAGTGTATGTAGAGAGAACCCTTAGGGTTTGGGCGATCTCGAGATGGAGGGGCGCAAGGGGGTTAACGGAGTTCTTCCTTGCGTCCGGCTTCCGCCACCTCCCCCGCGGCTCTCCTCGGATGTACCATGTTGAGGAGGTCAACCACAACGGCGTTGTGGTTGGGCTCCTCGCCACGTTCACTAACCCTTTCGATGCTTTCCATCTCCTCGGGCGAGCGTATTGGGTTGGTTGTGAGTTCATAGCTTTCACCACCTACAATATCTTCACCGACTTCCAGAGCATCTTCCCCAACAACGCGTTATGCACACGCTCCCGTACCCCATCAACAACGGGGAGGAGTGAAGGACGGCGTCCGGAGGAGCGAGGTGGCGTTGTTCCCCGGGAGAAGGAGAAGAAGAGCCCGAAGAAGAACCCGCGTTTGGTGCCCCCCGATCTATCTAGCTTATCGTATTAGTTTTTTTAAGTTGTATTAGTATTTTAAGTTGTAAGGCTATCATGGAGTTGTAAGGTTATCGTGGAACTATGGGTTGCAATAGTTGTGCTACTATATATATTGTGTGTTTCGTGCTATTATTTGAAGATTGCTATGGGTTGTTCTTGCTTATTATTTGTGTATTGCTATGGGTTGCTACGAGCCCGGGTTGCTACACCTCAATCTCACCACACACACACCATCTTCAAAATATTGACCAAACCATGGACATGCAACTAGGAGCCCCATGCAAACCCACTATGGACATGCCACTAGGAAATGCAAACTAATTTACTTCATGCAACTCATTTAGTTCATGGAACCATAGACATGCATACAAACAATTAACATTTGGTTCTATTGCATCAAAAAATGATCCATCACAAAATTTAGTGCAAGAAAAAGGCCAAAGTAAAAATAAGTAACATTTTATTTGCCGAAGTTAGAGGTGGGCGGGTGCCTCTACGCGGGTGGGCTGGTGCCCCTACGCGGGCGGGCTGGTCTCTATTTTGGGCATGGTTATTTTCTCAGGGTCCATTTTCTCACAGCCCAACATCTATTCGGCAGCCCAGTTTTGTTTTTTTACTAGAAACTGAATTTGGGTGTGTACTCTGTTAACTGAAGAACAAAAACAGAGGAAACAGAGCATCAACACTGAATATGGCCCCTGAGAATATCATTACAATAATTCAAGCAAGTTTTGCTTCACACGAATTCAAGTTCATCTAACAAATGATCCCTGGCTAACTCAAACTACTGGGCACCCCTGAAACTAGCTGACTAACTAACTGAAACTATAACAAATTCTAGCGATTGATGAACATCAAGTAAAATAAACCCATAGCAATGACACAACCATAAAATGCTCCCGCCATCATATTTGCTTGTTGCTTCAAATCGATCAGATGCTTTAGTTGCTTGCCGATTTTCTTCAATTCACACTTCAGTTCTGCACTATGCATCATCGGATCGGCTCTGTCCGCCAAATTGGGGGCTTGTTCCACGGCAAGCCGCCCCCCAAAATTGAGCTCTTGGGTTGGGGTTGATCCCTCCAATTTCAACCTTTCAACATATTCATCGAGCCACTCAAAATGCCCACATTTCTTCAGAACCTGAAAACAGGGGGCCGGCGGCGCATGAATCCTAGATCCACCACCCAGATCCACCGCCCTAATTCGAGGGAAAAAGAAAGAAATCGGGAGAAATTAGACCATAGAATTGGTCAAGAACACAGATCTAACCTGCCCCGGCTGTGGCTTGCTCAAACATTTCACAAACTCGCGCCCACGGTTACCATTTTCTTCCCTCACATAAGTCAAACGCTTGAGAGGCTCCATGCGTGGGCAATCGGGGCATCTTGACAACGGCAGTGGACCATACTGCGTCCATGGTTGGCAGGTGGCTGAAGTCGAGCTAGACATCACTCGCCGGCGGCGCGCTTCGTTGCCGGAGTAGAGGAAGAAGAAGGTGGGAAAGGAAAGGGGCAGGGTAAGCAATGGAGGGGGGCGGGCTGGCTCGGGTCGGGCCACGCTGATGAGCACGGGCATGCTTGTGTGGATAAGTTGTGGGTCCCACCCGCATGCGAGTAACTGGTGGGTCCCGCGTGTCATAACTCAAATCGCTAACCCTCGTGTTTTTCATTTCACGGTTAAACAGAGCCATGTCGCATTGAAGCTATTTACACGCTCAAGTGTGTCGCACCACAGCCATTCGCTCGAACAACGTCGCACTCAAGCAAATTCACATCAAACATGTCGCACAGGAGCTATTGGCCCATTTTCAACTGAAGCTCAGGCCGCTCCGGGAGCGGAGTCGTGCGGAGCGGCGAGACTCCGAACAGACTCCGAACAGGCCCTTACTCATTGTCCTGGTCTTTACGGTGCGCACATATTGTGATGTTTAGGTTTGGCTAGTTCATTCTCCACTCCTAGTCCCCACCCATCAAGTTATCTGTCAGAGGGGCAAAGCATGCAACATGTATAAAACTGGCACATTCGTGCGGCTCTGAGCTCCTGGCCTGATTCACGTAAACTTTAGCTAGGGATGACGACAACCTCCACTTTCTGTTGCATCAAGTTCTGCATCCGTTGTCACCGAGTCGAGAAGACACCACGGGCACTGGCAATGACCACAGTGAAGCTTGAGCCAGCCTACACCTCGATGAATATCACCACACATCAGCTCGCCCACCTCCGCTTTGTCGTTGCTCGCCAGTGTTGTTCCTGCTGCCTTCATGCTCCTAGTCACTACAATCGAGATAGACACACATGAGAGACTTGAGAGAGGGTGGTACGTGGAAATGAGCGAGGGTTGATTCATGCGCCTCTTTGGTAGATGTTGGCTGGATTTGTTAGCACTAAAGTGCTCTGTCCCAAAAATCTGCAGCGCCATGGGCCAATTTTTTTTAACTAGATGATGCCCCGCACGTTGCTGCAGAATTGAATATATAGTTTCTAAATATTGCATAGAATACTAATTGAGTTCTTATGATATAAACTATGTGAGAAATTTCTGCACAAGCAAAACAATCTTAAAATGAAAAGGTATGCACGTCACAAATAACTGCAATGTGATTTCAAACTCACATGGAATGGAGTAATACATGTTTTTTTTGTCCCTTTTGCCTTTTTGCTTGCCTTCCTGTACATATTAATTTACGAAGAAGTTTTTTTTACCGTCCGTTTCACTACTAAGAGAGGAGGGCGGTAGTAGAATGAAACCCAACCATAAATCTTGACGTGCAAGGCAGGTGCACTGCCGATTTTCCTTAAGAGAGAAAAAAATAGAAATACAGTGGCCATGTTTATAAGGGAAACTAGGCCGAAAAACCTTACAACACACGATAGAACATAGGACAATTGTGCAACAACCCAACCCTTGCCGATGTCAGCAATTCAAACTCCTCCACCAACATGCCTACAACCTGAAGAAGTGTGGGGACTCTTCGGAGATGCCTTCAAGAAGCATCGTCGCGGGTGTCCTTCGACCCCCATCGAACCAAAGCTTTTTCCTAGAGTCGTCCACAACAACCCCACCGGACCTTGGGAAAGTCTAGCTGGGGCATTTTTGTCTATGATGATTCTCTAGCGTAGGTCTGACTAAAATAAAGTTGTATGAGATATGACAACCTATTGGAATACCACCTGATGATGATGAATGATGTTACTCCACTTTAAAGGTGTGTTTAGTGTCTCACCTAAAACACCATGTAGAAGTACTACGACCAAACCTTGTTGGTGTACGAGCTATACTCTGAGAGGTATTTTCTTGTGCATTAGCAATTGAGATGTCTCTGCCTTCATATAGTTTGCGAGGAGAAGAAGCTACCAAGGACAGTTGTGAAGCCATCAAGACAGTAATTATGTTGAGGAATAAAAACAGTTTTATTTATTGCTCTCCATGATTCTGCATCATTTCAAGGAAGTTACTGAATTTGGTGGCTTGGCAACTGTAGGCTGCAGGTGATATTTCAAAATACAAGCAAGAACGAGGACCAGATAGTTCATTAGCTTATTTGCTAAAGCACTGAGCACGTACAGCTTCAAGACAGAGCAGCCAATAACAAAGGAGTACTGTCGGTTTCAAGTTCAGTTCAGCTAAAGGCAATGGCAAGGTAATTCCAACACAGGGATGGCTAGGGCAAGGTCAAGTGATGCCTGCCTACCAAGACGAAATAGATCACACCGTGTCTTCCTACAAAAACTTCTGTTGCATCTGTCATTCTATGATTCATATTACTCCCTCCATTCAAGTAGATAGGGCCTAATATGTTTTTGAAATAGATAATTGATAAGAGTAATAATATATGAGATGTATGATATAAAAATTATATCATTAGAACGCCTTTCACAAGCGAATTTGACGGTAAACTTTGTGTAACATGCATGTCGTATATTATTAATCTTATCAATGGTCAAAGCCAACCTAAAAGAATGTATTAAGCTTCTGTTGAATGGAGGGGAGTAGCAGCTTTTCAATTTTTTTTTTTTGCAATGACGCCTTTTTAGAAAACAGAAACTTTATTACCCTGGAAATCTGCATCAAGATGCATACAACCACAATTTATTACATTGTCTATTGGCAAATATAGCGGCATAGACTGAAATACACAAGTTTGAGTGGCAGGATACGAATACGAGACTTTGGCAGTCGCATGAACCGACCATAGTGTTTTGATGATTCTCTAGTGTAGGTCTGACTAAAATAAAGTTCTATGAGGTATGCCAACCAATTAGAATAGCACCTCATGATGACGAATGATATTACTCCACTTTAAAGGTTTGTTTAGTGTCTCACCTAAAACCCAATGTACAAGTACTACGAACCAAACCTTGTTGGTGTACGAGCTGTACTCCAAGAGGTTTTTTTGTTGTGCATTAGCAATTGATATGTCCCTGCCTTCATATAGTTTGTGAGGAGAAGAAGCTACCATGTACAGAAATACCAAGGACAGTTGCCAAGCCATCAAGATCTTAATTATGTTCAGGAACAAAAACACTTTTATTTATTACTCTCCATGACTCTGCATTGTTTCAAGGAAGTTATTGATCTTGGTGGGTTGGCAACTGTAGGCTGCGGGTGCTACATGTATCCAAATACAAGCAAGCATGATGACCAGTTAGTTCATTAGCTAATTTGCCAAAGTACTGAGCTCGTACAGCTTCAAGACAGAGCAGCTGATAGCAAAGAAGTAATGTCAGTTTCATGTTCAGTTCAGCAGTTGAGCTAAAGGCTCTCTGGTCGCTTGGTTTAATTAATTAAAGCAGCGATGGCTGGTAAGGTCTGGTGATGCCTGCCTACCAAGACTAAATCCATCACACCGTGCCTTCCTACAAAAGCTTCCTACAAAATCAGTTCAGCACGTGCCTGATTCATACTATCAACTTTTTTATTTCGGAAACGACTTTTTTTTTCTAGAAGACGGAAACTTTATTACCCCGGAACTCTGCATCAAGATGCATATATAGAAACCACAATCTATTACATTATCTGTTGGAAATTGAGAAATAAGTATAGACAAATCAATATGTGTGTTAGGCCACATTAAAAAGCCTATTACACGCACATAGTCCAGAATATGATATATATACACTCTCCGGTCAGCCATTGCAGCCTCTTGGCTGCCAAGCTCTCTATCGGCTTCCTGTGGTGCCACTTCTGTAGCAAAGCCCATAATCGCTGCTAGCGGGCAGGTACACACGAGTAACACCTGCATACAAGATTGAACTTTGTTATCAAACATGATTTCATTTCAATATATAATAAAATTGAAAAAGAACTGTGCAGCAAAACCCAGCCAAAATTAGCGATTCGGTATTTTCTCCTAGAGAAAGGTGGTAAAAAAAAACCACCTAGCAAAGCAGTATTTTTCTCTTATGCTTCTCTGGTTTCAGAAAGAAATGATGGACCAATAAAAGTCAAGTCTCTTTGGATCGTTTTCAGGATTTTCAAATAGGACATCATGAAGATATCAACATCTAATTAAGCACTCACTTTTCTACAAGACAACGCTATCTCAAACCTAATGTATGTGCAATGAGTATATAGCTCAAATAATTTAATATTCTGTAATAATATACAAAGCTACAAATTTAACATTTTACTTTTATTAAGCTCCAATCATATCATTAACGTAGTTGCTTTACAATGCACTTTGGTGTTTTTTCCACTACGAATATGCTCTATGCACATAATTAATTAAGTAACTTTAGTAGGCAAGATGTCTCTATCATAGCATCTCTAGTCCCAGGGATATATTATAGTCTAACTAGAATTAACATGAAGCATGTGCTCATTTTTTAGGAGTCGATGGATATGTCCTTTCCAGTGTTTCACTACCCTCCGGGAGTGCAATGTCGTACTAAATGATAAATGTGCTCAAAGTTAAAGGATGGATAAATCTGGCTGATCGATACGTCGGGCCGAGCAATTTCTTGGTGAGATGTTGTGATTATCAATTAATGATCTGGAGTGTTGAATGAGCACGGTAGAGTACCAGGCGCCAAACAGTGACCGCTACTGGCATGGACAGTGACTCAACCTACATTTTTCCTTTTGGAAGTTTTGGCAAAATTCGTTTGGCAATGCAAGTCTACAAATGGACATGATATGTCGTCCGTGCATACTGATGCCAAAAGGTAATAACCTCCCTTTATGAAAGAAAAAATCTAATAGGCAGAAGCCAAAAAAACCTATCGTATTCGACTGTTTTTATGTATATTCTGGGACTTCCATATGCCTTCACTCTTATTCCTTTTTCCATATGATCTCCTGATGACTCTTCAGCTGGAGTTTGGGTGCAATGACGGCACACAATAATTTATATGTTTGACAATTGTAGTTCAGTTTATTTTTTCGATTAAGTGTTACTACTAGAAGCTGTCAAGCGGGAACGGCCCCAGCGTCGTCGTGCGAGAGACTCGGGCGGTAGAATCTCCTCCGCCGCTTTATACATCATCTGCATCCTCCCCCCCCCCCCCCCCCCCCCCGCCCGCCGTCGTGCAGGGCTGCAGGGCAAAGCCCCTCGCGGCGGTGGGCGGCAGCGGGAGCTCTCCCTCACGGTGGTCTGCCTCGGCTCAATCTCGCTGCCGATGCGTGGCTCGCGCCGCCATCGGCCTGGCTGGGCCTCCCCACAGCTCTTCACTTAGCTTGGTGCAGTCCGTCCTCGACATCCTGCCACGTGCAAGTGATCCCTACTGTCGGCTTGGTTGGGTGCCGATTCACCAGATCTGGACAACCCAGGACCAAGGCGGCACTTGTGGCTAGGGCTTCGGTCACCCCCGGTGGTACAACACTGTGGTGGTGCTAAGAAGCTCCATGGTTGTGCTGCGGTGGCCCTGTAGACGGGGATGGTGGCCGGGATTGATTGATTTGCCGGATCCGGCTTCTCCATCTTTGTCGGTCGTGGGCCTATGGCGCAACGTGTGACCACTCTGGACTGCAGCATCGACCTTACTGTAGAATCCGGTGGCCATCGAAAGATCTTCATGGGGGTTCCTCTCTCTAGATCGGCGAAAGATCTTCATAGGGGTTCCTCTCTCTAGATCGGCGGTGAGATGCCATCGAAATATCTTCATTTATCGTTGCCTTCGGTGGTGAGATGCAATCTACAAACGACGACTGGTGCAAAGGTATGGTTGTAGAGCAGCGGTGGTCGGTTCTCTAGCCCAGCGCATGTAGCTGTCGGGATCGTTGAGAAGTGGCAGCGGCAACACATCATTGACTTCGACGAGCTCTTGGTTGAAAATCCGTGCCTGACCCAAGTTGATTATACCTGGCAATGTTGATGTTTTTACATCGTTACCTTGTTGAAGGCGTTCCTCAGATATGCTCGGATGGTTCTTTAGGGTGAAACCTATAATCTGACCTTTGGTGGTTGGATCCGAGGACAACAGTGCTGGAACGTTGCTCTCTTTCTGAAGATATTGATATTTGATAACTTCGTTATCCTTGTGGTGTCATGAGATGGCTGGTGTGGATATGATGATAGTATTGGCATAGTTTTGGTCTTATATGACTTTGCTATGTGTCAGCGTGTTTTGTGTGTGTGTGAGAGAGAGAGAGCTCGTTGTTGGCTGTGTGCATCCAAACTATGCAAGGGCCGGGAGTATGCTCATTACGTTTGTATCCACTTGATGCTTCATTTTGAGTCAATAAATCCATCGTTCTTCCATAAAAAAAACTAGGAGCTGTCAAGCAGTACGTGCACATGGATGGCGATGGATGTTGAATCTACAAGCACATTAAATAGTTTATTACAACTATTAACTGGACAGGGAAACAACAGAAATTTGCATTTGGTTGCCGTCTCGTATTCGTACGTGTGCATGGTGACTGCTCATATCATGGATGGATATGCAGAAGGCAAGAAGCCACTCCCGTACGTACAGTGCCCTTGACAATCATAGCACGGAAGAACAGTGGTACCTGCAAACCACCTGGCTTGGTGAACACGTATTTGCTCATGCTGACGGCCCTGGAGAAATGGAAGAAGGGCCCTTCGTTTACTGCATACGAACATGCACGTAGCCAGGACCGGCCCTAAGCTAGCCCTACATTCATACATTCATAAATGTCTCGCGCAAATATGCATCATTTGATATTAGTTAGTGAATACACAAATTTTATCGCATTGTGTTTGTTTTAGGAGGATTTGTCACAAGTGCGTGAAGGTGAGGCCAAGTTGTATGTCTGGAATATGTACACAACAAGGTTTTGGATTCCGAGCATGCACGCAAGCAGGACCAGCTCTAAGAGAGGAGAAGATCAACAAAAATGAATCATTTCTTTTCTTTTGTGCAGTAGCCATTGAGATGTCCCTGCCTTCATATAGTTTGTCAGGACAAGAAGCTACCATGGGCAGAAATACACCAAGGACAGTTGCCAAGCCATCAAGATCTTAATTATGTTCAGGAATAAAAACACTTTTATTTATTACTCTCCATGATTCTGCATCGTTTCAATGAAGTTGTTCATCTTGGTGGCTTGGAAACCGTAGGCTCCCTTGCACGAGTCATTCTATGGTTCATATTAGCAGCTTTTCTATTTTTGGCAGTGACGCCTTTTTAGAACACCGAAGCTTCATTACCCTGGAACTCTGCATCAAGATGCATACAACCATATTTTATTACATTGTCTATTGGCAAATCTACTGGCACAGACCGAAATACACATGTTTGATTGGCAGGATATGAATATGAGACTTCCGCAATGGCATGAACCCATTTTCCCGGAGTACAGCTTGTACAAGAAGGTTTCATCGTAGTACTTGTACATTGGGTTTTAGGTGAGACACTAGACAGACCTTTAAAGTGTACTAACATCATTCACCAGCATGAGGTGCTATTCTAATCGGTTTACATACATCATACAAATTTATTTTAGTCAGACTTACGCTAGAGAATCATCATAAAAAAGGGGAAAATTGGTTCATGCCACTGCCAAAATCTCATATTTGTATCCTGCCAATCAAACTTGTGTATTTCGATCTGTGCCAGTAGATTTGCCAATAGACGATGTAATAAAATATGGTTGTATGCATCTTGATGCAGCGGTTTAGGGTAAGAAAGTTTCCGTGTTCTAGAAAGGCGTCACTGCCAAAAAATAGAAAAGTTACTAATATGAATCATAGAATGACACGTGCAATGGAGCCTATGGTTTCCAAGCCACAAAGATCAACAACTTCCTTGAAATGATGCAGAATCATGGTGAGTAATAAATAAAAGTGTTTTTATTCCTGAACATAATTAAGATCTTGATGGCTTGGCAACTGTCCTTGGTATATTTCTGCCCATGGTAGCTTCTTGTCCTCACAAACTATATGAAGACAGGGAAATCTCAATTGCTACTGCCCATGGTAGCTTCTTGTCCTCACAAACAAATAATATCTTTTCTTTTCTTTTCTGTTCTTTTTTGGTACATAAAAAAAGTCCACACACTCAATTTAAGGGATTAAAGTACTAATTTGACATAATAATGCATTAAAAGTTGTTACATTGGGGAAGAAAAGTCTATTAGTGACATTTTCCTGCTAATAAACTGGCATCTTGGGAAACACTAGTTACTTACCACCACACGACAATGAATACAGTGCACAAGCACATGCATATGTTATGAGTCCACTCATCGTCTACGCCCGGCGGTGTGACAGTCTGCCGACCTCATCTTCGTCTCATTCTTGCCGATGGACGTTGAGGTTGGGAAGGCCTAATATGGCGTGATTCGACCTATTGAATAACACCACGCGCAGGTCGATTGTGCGCCATCTGATGTGCTCCCATCAAATCCACATATTTCTCCTCGTAATGGAGCCACTTGGTCCGGTCCCTCTTTGCTTTTAACACTTGCCCGACGGTTGTCTCGTTGAAGATTTCACCGGGGAAGACGGTCGAGGCGTAGACGGTATAGCTCCCATCCCATGCTTCCTTAACAGTGAGGCGAGGGAGAGCACGTTGTGGCCGATATGACCTGTGCGAGCGAGTTCTCTTTTGCCCATACAGGATCTGCATGTACCCGTGCTCGACATCCAAAAGAAAACACTATGATTTCAAATCCAAATCCGGGAACTCGTTGAAGAGGGTGGGATCCTCCTATGTGGCCTCCAGCACGTCACAACGATCCCACTCCTCCTCCTCGGTGATGAGGGATGACTCCTCCTCTGCCTGCTTCTCGTCCAAGGATCCGTTCCTCCTCGAAGAGCGACACAAAGTCCGATCATAGGAGTCAGTGCTAAATCCGGCATATCTCGAGATTTGGGTCCTAAGCTAGGGGTCTTAGAGCGATGCTAACATGGACACAAGATTTTACCCGGGTTCAAGCTCTCTCGAAGAGATAATACCCTATGCCATGTTTTTGATTGTATTGATATGGGGTATAGTACAAAGTAGATGTATCTACCACGAGGTTGTTGTATATGATTCTAATGAATATGAGATCATCTATTGACTAGCCTAGCCTCAGTTTATATAATGCACTAGAGGCCTAGGATTTAGAAGAGTCATAGTTTGTGGGCCAAATCTTTTGGAATCTTTCTTGTATATGCCATGGGCTGTCCAAAGTGGCCCATTAGTGAACCGCCATAGGGGTCCTCAGCCCGACCCACCTGGCCAGGAGACGATGTGTTGAGTACCCCCTAGTCCAGGACACCAATAGTAGCCCCCTTGGGCTGATCTTCAAGTTGGGGACGCTCATTGGTTCTTCTAAAATGTTCTTCGTCTTCTGTCGTCAGTCTTGAAAACTGGTTCAACAAATCTTCCCATCTCCGATCTTGAGGATCGCCGAGGTGTATCCGAAGAGCTCACATGTCGGGCATCCTAGGACCCCTTTAAGTTCTCGGCTCTTAACAATGCCTTGCTATTTTTACGCCACACCCGGGTTCAAAGTTTTTCCCATTCGGTGGTGTCCTCTTGCAGCCGAGCTCCAACGCCGGACTGTAACCAAGGTGTCTTTTTGTAGCCGAGCTCCAATGCCAGATTTTATCTGAGGTGTCATAGATTACCTCGGTCTTGAAAAAATTGAAGGAGTTCAGCCGAGCTTAATGCCGGGAACGTCCTCTGGGGAGCCAGCCAGTTGCACCTGAGCTTTATGCTGGACTACTTTCGAGGTGGTGCACCACCTCGGCCTTGGGCTGATTTTTTGTGGATTTTTTTGATTGGCACACTTTATTCTCTACCGAGATGTATAGCTAGTAGCCCTCAAGGTATGTGTTGGCCTAAAATCCGAAATGCACTTGAAGGATAACAAAAAACCATTGATCCCAGTAGACCCTAAGAATCAGACCGATTTGCGAAATCGGCCTAAGGATCAAGTCCTTGCTCGGCAACATACATAGGAATGGAAATGCGGTGCATAATAGATCCCGAGACTCAGGCTGGGTGCGGCCGACCAACCTAAGGATTGTAATCTCCTCGGTAACTGTTTTGCACTTTGATTTTTTTGCATTGGATCATCAAGGCAGTAGCCCTCAAGACTTGGGTTGGGCACATGGCCAACCCTAGTATCGTATCTTCTCGGGAACTTGATCAGTCTATGTTTGAGTGTCGCCATCACCGAGGTGCAGCTTAGTAGGAAACATGGCGACCTGCGGGTTGGAAAAGTTTTCCTGAACATATCCATCACGCAGGAGACCTCGATCAAGAGGATGTCCTGCCTCCTAAAAAAACATAAAAAGGTTAAAGATGTCATAAGTTCGAAACCAAAAACTTATGTAAAAACTTTATGTCTGAATTAAATCAAGGTTTTTTGTTCCAATCCAAAATTGATCTTAAAAATTAATTTGTGCTTTCGTTGTTCTTTAACATGATACCTCCGATCTCCAAACTTCAAGCGGGTTAGCCTTCGGCTTCAACCTGCCTTTCCGAAGGCCGAGGGCTTCTTAGGGTAGTTTAGCGAACTAAACTTGAGCGGCTCTAGAGAGAAGGCTACTGACCACACTGTCGCATTGGGAAAAAGGAGTAGTAGAAAAAGACTTTGCAATGACTTAGAAGAAAACATTTATTATAAAATGACACATATGAATTTGAGAGCCCCATTTAACTTATGGAAAAGATGTTTTAACAATCAATGTTTTTAACAAACAAACTTTTTGTTTAAGACAAATATAACTTTTCATTGAACTGAACAAAAATTGAATTTTTTTTTAAAAAAATTGAGCATCAAAGTGACTTAGCTAGTTAATGTGCTCAGTGTTGATGACATGGTACGGGTTTGTGGCGGGACGAAGACGTCCATCAAACTTTTCCAGATCCATCGCGGTGTGTATTCCTCGGCCTAGCCGAGGTCCCAGCCCCCAAGACTGTCCCGAGGTGACCGAGCAAGGAGCTCGGCTTGGTGGTGGCGATGCAGCATGGTCGATGAGGCGAACCCCTGGTCCAGCTGCCGTAACGAAGTTGCTCAGTGGGGTGATGATGAAGCCCCTGGGGTATGTTGATGAAGAGATGGTGAAGCCCTCGGTCTAGCCGAGGTGACAGAGCAGGTTGGCGGCGTGACGCCGGAACCCCCACATCAGTCTATGTGTCGAAGCAGGTCAGCGTGGTGGTCGTCGCTTAAAGAAGTGACAGTGTAGGTCAGAATTTGTGATGCGGTCAATGTCGGCCTGATGAAGTGACTGCACGGCGACGCCAGCGCGCCCGACCCAACAATAGAGGAAGAAACATGGAGGGGAGTGGCGGGTGTGGGTGGCGGTTATGTGAACTTTTTGGACAGGGAAACAATCATTTCATAGCCATAGGCAGTGATAAAATGTGGCATGTGGTAGGGATGGTCGCGGGCAGTTGGCACACAGCAACCACCATTAATAATTGGCACACTTCCTGATGCCATGAGTGTCTGTCGTCTACACCATTCGTAGTGCAAGGAGATGAGCCACGTGGGATGCTGAGCAGGGAGGTGAAACTTCTCTCCCACACGAGTGGGTGGCAGATGGAATGCCCACAAAACCAATGCCCTACAACCTCCAAATATGGAGACTCATAGGCTTGAGATGGAGTGCCGTCAATAAATATGGCAATCCAGCACCATATGACAACTCTGCAAGAGGGGTCTTTAGGGCCAAAATCTCTATATAACCGGCTATTATGGAGACTCCACAAAAGTTGCTCTAAGCTAGCGCAACAAAGGAGCCCGCCCAGGGCTCTTTGTCTAAAGGGCCTAGGCGGGTCCTATATGTATGCTAATGGGTGTTACCATAGTGGGAGGCTTAATCAATGTAGTAGAGTCATAGATTCCTAAGTGAATACACAAAGATTACCAGATTGGGTTTGTTTCGGGAGGGTTGGCAATGAGTAGGGGGACGAGAGGGAAAGCCGCTAGTATCTCTGGAATAGGTTCACAATTTAGACATCCCGCATTTTTTGAGATAATTAAAGCAAAAAATCTAGTGAACAACACTATACTTACAGAATTTTAGATTCTAAAAAATTATGCATACTATGCATGGGAGGAATCCATCATTTTTTCCTCCCATGTCTCTCTCATGTTCCCACCTCCCCCCATTGATAGTCTCTAATTCTACTCAGGCTACTCTACATTTTCCCTCCAACAATATAGCTAATCGGTTTAGGGATGGCAGGGCGGAATGGGGTGACGCTATAATGGGGTGACGCTATACATAGTAGTGCGAGGTTAGGGTTTGGACAGAAGGCATTTGGCTTAATGACAATTCGGAATTATGTTCTCGCGCTAGTGGGCGGCATGTGTCAGGTTGCTGCCGGCTCCCTCACACAACTTCTCCCCATGGTGGATGCCGATTGTGGGAGTTGTATTGGGTAGAAGCTTCATCTCAATAATCCTGGTGGATAGCCATATCTTCCCAAAGCTGGTCAAATCTAACACTCTTATTAGGGCCAGCGTGGTTATAGTTTAGGAGAGGAGGATGGATTCATTTGTCGGTTGTCTCCAATCTAGGGTTCCATGGTAAATCTAAGTTGGTTTTGAAGCATCATCTCCTATCTTATCCATCTACTGTGTTCTCAGATGCTTGAGCTACAAATCAAACAACAACAAGTCTCCAACGAGCTTCATGAGTGGCTCATGTGGAGTGGTGGCCACTGGTGGATAGGTTGGCAAATATCAACACCCCTTCTTCAACCTCCACATGGGAGGCCCTCTTCAGCTTGCACTATGATGGCAACAACATGACCAAAGATTGTGTCCAAGGAATCTCCGACAATATTGCCGCTCGTCGGAGAACTCAATTGCTTTCAATTGCGTCCTTTGGGGTCCTGTTTGCAAATGACAAGGATTTGCTTAAAATTTCAATTTACTTTAGAGTCCTGATGGCTCTATTTTGTATGCATTTTTACAGCTCATTTGTTCCATATTATCTCCATATATTCCTACTAAACCAAGTACATGTCCATTATGCATGGTTTTCGGTTTTCACTCTTTCCAGTATCACTATTGCATAGTTTGTGTTTTATTTACTTTTATTAGTTTCCTTAGTTTCGTTATGTCTATAGGTGTTTTATATCAACCATGGACCAAGCATGATGAAACAGCAAAGGTTGGGTTCAGTACAGAGGACTTCCAGGCACCAGACTTAGGTACTTCAGAGTGTAAAAATGACATTTTTTCAAAGTGCTCAAAATCAAGATGTAATACCACAGGTGCATCGACTTCGTTCATACTCCCTCCGTCCGAAAAAGATTGTCCCTCAAATGGATGTATCTAGAACTAACTTGGTGCTAGATACATCCATTTGATGGACAAGCTTGGGACAAGCTTTTTTGGATGGAGGAAGTACGATTCTAATGAGCCCAAGAACATCAAAATCCGAGTTCGGAATGACAATCATTCTCCGAAGAAGACCAGAACGTTCGATGAGGCACGGTACAACTATTCCATGCCCAGAAAAGCCCAAAATGGTTCAGACCAGATGGGATTCTTGGTGGATTTCATTCCTATTTTTTTCCTTCGACATCTTTGGCCATCAAAGGAAGTTTTGGCAGAGGATTTGGCTCATTTAGAGCCCTTGAACCAAAGGAGAAAAGATACATCTACGGATGAGGCTTTCATAGAGCCCTTATATACACATCTTCAAGCCTAGTCGCCTCCATCATCCATCATACATCCAAGATCAACCTCCATCACGTCTATTGCTACTCCAACACCACCTCGATAGAAGAAGGAACGAGATCAAGAGAATGAAAAGAAGGCTCTAGCACACACCGCATCGGCCTTCGGGGCGGTGCTTTCTCCACCATGCCACCGCTGACATCACTATCTCTACCACGCCATCACCATTGGTGCCGCCTCCACCAACGCCACCATGTGTTCTCCATTCCCCTACTCGATGCCGACTTGTAACTTGACTCTCTCCTCTATGTTACCCGACTTTATGTTTGAGCAGTTGTACTTGTTCTTGGGTATATGGATGAACTCTTTATGATTAGAATTAAATTCAATTAGGCGAGCCAATATCTTTTGTTTATGTTGGAGTAGCTTTCATGTGTGTTGTGAGGAGTACGCACACAACATTGTCGCCTTGATTGGCTGTCAACCATATTACTGCTGTTGTTCTGGGTTAGGATGTTGAAGTAATTTGAGGTGCCGGTAAAAAGCCTCTACCTTCACCTTGTCCAATCCGTAGGGGAATAATAGAGAACCCTTGGTGAGGCCGCGTCTGTATGCGAAACCACCCACTTAAGCACATAAAGAGTAGTTTAGGGACAATTCATAGACCTATGCTCCGCACTTGTTGCATGGATCATATTAGTAGTGATGACTACGGAACACTCGAGTGACCTTATTGCTCAAGCACTGCCTATACTAACTTAGCATTTTTCTTATTCCTATTTTTACCTTCAGTTCGTATTCTAGGTAAAGTCATTTTTATTCCGTTTCTGCAGCGATTTATAGAATAGGCTATTTCCAAACTCCGGTTTGGGCGTGTACCGAATTTCATCGGTGAAAATGTAGTTACAGGGTTAGTAGTGCCTTACTTTTCTCTCCTTGCGGGTTCGACACTCTATTTGCCATGGAAATGCTACAACACCCTATGCTCTTGCTGTACATCAAGTCCTTTCGATACTGATGTATAACTTTTGATATTATAAACACCGGTTTCGAGGATCTTCGTGTCCCTTGTCAAAAAAAAATACATGGATTTGTACAAACTATAGAGTACGCATGTTGGCACGGCAAACTATTGTCCCACCTCACCAACCAAGAGCTAAGATGGAATTGGAGAACATACTTCTAGTTGATATCCATACAAGTGTAGAAGTATCTATTTGTTTAACAAGCAGGAGCTAGGATGGGATTGGTCTAGAAGTATCTATTTACAAATGGAGTACTCTCAAAACATTTGGACATTTATCTATAAGATGCATACATTACTCTTAAATACAGTAGCAAGTTTAATCGACATACGGTGACCTGCAAACAACATAATGTGGTAGTATTGGTTTATTGAGATTTACAAAGATATCCATTGTAACAACATAGTGGCTTAAACGTCCGAAACAAAATAAAAAACATTCTTTGTGACAAAATACAATTTATTTTTAGATCTGTAAAATATGTTTCTACTGATAAACATTAGAAGAGTATGAAAAATAAGTACCTAAGATGCAGGAGCTTGTGTAAATTGTTCATTTTTCTTATTATTTATACAATAATATTTAGGCTGGTATGTTGAATAAATAATATCAATAGAAACAATATATATCTTTACATCAAGATGAGTTATCACGAAGTAAAAAAGTTCAATATAAATAGGTGGTACTCCCTTTATTCTGGTAGATAAGCCCAGTTTTTACCGATGCATTAGCTGGAAATAATGACATCCGTTAAATCGGTTTTATTAATTGTGCTTATCTATTAATTTTCCTAAAATTAACTATTTTACATAAATATTGTGACCAACAAAACATTCAAGCGTCACATCTTGATTTGACATCTCCGGTATGTATATACTTTATCAAACAATTAGCATGTAAGTAAAAGGAAATAAAATTAAAAAATGTCATAAACACAAAATATTCTAAATATCTTTTCAAAATCCAAAATCATTTGAAATAAACATAAAATACTCTCAAAATAATATTAAAAAATATTCATAATTTATTTATGAGTTTTTACAAATTTCTCAATTTAATACTACTTCTAAATTTATTCACCTTTTTATATTTTCCTAGATTTTTTCATATTATGTTCTATTTTGTGCTTGTCTTCTTGTTTGCAAAACGAGTGTCGCGTCACTAGTCATGCCACTTTCACTATGTCGGTGGTCAAATCATCAATCTTTATATATATTCGAGTATATAATCACAAATAAACTCAACCAATAGTTACTTGATAAAAAAGAAGTTTAGTCCCAAATTTGTCGTGGTCTCTTTTTCCTTTAGGACATCCACATCTCAACATGAAAATTGTACTAAGGTCCAACATTATAGGGCCTCCATGGAACCGTGGGATTACAAATAACACAGGAATTTTGAGATTAAATGTTTGCTTCATTGGAAGAAGACACATGCATGGTGCATTCAAAATTGAAGTGAACTTCCCCATGAGATGTTGGTTTCCTCCAAACTACTAGGAAAAATTTCAAAGCTGAGGTTTTATTTTCGTGTAATCCAAGTATCCAAAGTGTATTTTTTTATCTTTTGCTTAGGACTGCAATCAACCATTGTTCCCGCAAAAAACCTTATTGGTTTTTAAGGTTGGCTGAACTTGGAGTGTCAAAACAAAGTTAGTGAAACAAAATTACTAAAACACTAGTAGTTTATATCATTTAACTCTATGGGCTGGTATTGACTATTATGTGGCTGCAAATCAACTATAGCAAGCTAGCTAATCAGTACGACATTAATTAATTATTGCAACTGTAGCTAGCAACACTGCATGCATCAGCAAGGTAGCATGGGTAGCCGTCTCAGCCCCATCAGCACTCACCATGGAAAAAGCAAAAGTGTGTAAATAAAGGAAAACAACAACAAGATGATGTCCTCTCCCTCCTCCAAATCGCCACAAGCTAGAGAGAGAGAGAGAGAGAGAGAGAGAGAGAGAGAGAGCCTTCTCTCTCATCTCTTGGTTGTGCTGCTACCTGGCGCTCTCTCCTTTTTCTCTCTCCTCTTGGGTAGCTCTCGCTCCCTCTCAAAGCAGTCAAGAGCTAGACCCTCCTGTCTCCTCTAGCTTCCATTCCATTCCTTTCCTTGGTACTAGTACTCTGATTCCCTTTGATTTCCCCAGCTGCCGCAGCTGCCAAGTCTCTTCCTCCCACTATCTCTTCTCTCCAACCTCCAGCCCTGCCAGCCGCCCAAACACCTCTCTCCTCTCCCAACAACTCTCTCTGGAAGTCTAGATCGCCGGCCATGATCTTCCCTCCTGCCTTCCTCGACTCATCAAGCTGCTGGAACACCAACCACAACCAGCTTCAGGTATGCATCCTTGCGGTCAATTAATTCTTCTCGCAAGATTTTGTTCACGCAAGAAAAAGAGAGAGAGAGAGAGAGAGAGAATATGTTCTAGCTAAGCTAGGGTTTGCTGATGGCAGATATACATCCTCTGCTGATTGCTGCACTATGTATCTTGGAATATACTCCATATACACATCTTGGCTGACGCTTAATTCCTGACCACTTAATTTGCAGCTGCAGCAAATCGGCAGTAACACTCATATCACTACTACTCCTTCACCTGCTGGCCATGGTCCTGGAGACGGAGGAGGCGGAAACAACAACAATCATGGTCAGCAGGAAGGATTAATGGCCACGGCCGGGGCGGGAGGAGGTGGTGGTGATGGTGGTGGCGGCGGCGGTGGGGATGGTGACAGCGCCAGCGGCGGGAACAACAAGCCGATGTCGATGTCGGAGCGGGCGCGGCTGGCGCGGGTGCCACAGCCGGAGCCGGGGCTCAACTGCCCGCGCTGCGATTCCACCAACACCAAGTTCTGCTACTTCAACAACTACTCCCTCACCCAGCCCCGCCACTTCTGCCGGGCCTGCCGCCGCTACTGGACCCGCGGCGGCGCGCTCCGCAACGTCCCCGTCGGCGGAGGGTACCGTCGCCACGCCAAGCGCAGCACCAAGCCCAAGGCCGGGTCGGCTGGATCCGGAACTGCCGCGGCAGGGACGTCGTCTGCGACGTCGACGACGCCCAGCACCACTGCTTGCACCACCGGCACAGCTGCCACTGCGCCGCCCGCTCTGCAGTACTCCATGTTCGGCAGCGCGCCGCCGCACAGCAGCCGGTTCGCCGATAGCTTCGACCCCGCGAGCCTCGGCCTCAGCTTCCCCGCCAGGCTGCTCTTCCCCGACAATGGCGCCTACGCTGCCGACGGTGGCGCGCAGCAGCACCACCACCACCAGGGGAACGGGAACGGCATGGAGCAGTGGGCGGCTGCGCACATGCAGAGCTTCCCGTTCCTGCACGCCATGGACCACCAGATGTCCGGGAATCCTCAATCAGCTTCGGCAATGCCAACCACAATGGCGGCGATGCAGGGCATGTTCCACCTCGGGCTACAGAGCGGCGGCGGCGGCGGTAATGGCGACGATGGGGGAAACCACCAGTTCCACCACCAGCCGGCCAAGAGGGACTACAACCAGCAGCAGCAGCAGGATTACCCAAGCAGCAGGGGCATGTACGGGGACGTGGTCAATGGCAATGGCGGCGGCTTCAATTTCTATTCCAGCACTAGCAATGCAGCTGGTAATTAGCTAGCTAGATCTAGCTAGCTTTGTTCTTGCAAACCTAGGTTGATGCATGGTGAAATGGGGCCGGGGATGTATAAATTCTTCATCACTACAATATGTGTTACAAGAACTCGATCCATCTCGTCGATCGAGATCTCTAGAGGAGGGGGGATATCAATGCATACACAAGAATGTTAACCTTTTGTCTGTTGAGTGTTTGATCACTATGGCCTTAATTTGTAGTACTATTGTTTAATTGTTGTTTCATATACTTAGCAGCTAAGTGTAGTGTGTTGGTAGCCCCTCCACATGCATGCTCTCTGGGATCTTTCCAATTGAGTTTTATTACTTTTTTCATGCATTAATTTGTGGGTATGTACCAATGTCCCGCTTCTACACTGTGCTAGTTTCATCAAACTCTTGATGATTTATATATGTCAGGTTCTCTTTGTATCAAGGATCTACTTCGCGTGTTTGCGTGAAGGTTTTTCACTATGGTTTTATTGAGTCTCGGATGACTGGTCTTTTTCAATTAAGTCACGGTCGACTCCGAGAATTGTCAATATGACTATGAGATGCATGCAAAACTTAACATTTTTTTTGGATATGAGAATAAACAATGCATTTTTTTCCACTAGATGAACAAAAATCACAGCACAGTGACTATTTTCTTAAATTTGTATGAACAATTTTGTAAAACCGGTTGAGACTTAAGAATATCTCATTTGACTGTCGAACGTCGTCTTAGATGAGCCTTTTCATATTTAATTTTGTGAGCTAGTTTGTTGCAATCAGAAGTTGTACTCCGTTAAGTTGTGATTCATTGTTTTGTCTGAAAATGCACAAGGCGTACACATATACTCCCTCCATTCCAAAATAGATGACTCAACTTTTTACTAACTTTAGTATAAAGTTAGTACAAAGTTGAGTGATCTATTTTGGAACGGAGGGAGTACTTATGTATTCAGTAAGGCTAGTTACTCTGATGTGTCATGTTGTTGGATGTATCGTGTATATGCTAAGAGCATCTCTAAACAATCTCCTAAAATTTAAAGGAGGATACGGCCGAGTATCCTTTAGTGGACGAGATTGGCTCCTCTAAACAAGTGTCGTTTCTAACCTATCTACTAAATTTAACAGAGCAAAAAAATCAACACAAATTCCGTGCAAATTTAGACTAGATGCGAACTTGCTTATGTACAAAATTGATTCGAAATAGAAATACATAACAAATTTACACATACTAAATGATATCTAGAACATTATTGCAATAAAACTAATTAAGCTACTAACTATTAGTCTCATTCTCGATCCTCATGCACTCCAATTCGGTGAGCCTTCCGATGAGCAGCCAGGTCCCTCGTCGCTGTCGGAGTCGCTGTAGAGGTCGATGACCTCCGTCCTCCAGGCCCCGTACTGCTTGTACTGCCGGTGCTCTACCTTGATGCGCTCCAGGTAGTAGCAGCGAGCTCCACCATGTAGGCCTCGCCGGCTTGCTTTACCTCGAGCACTCGCGGGCCTAACAGTCTTCATGTGTATCTCGTCGGGTGGCCACCTAGGGGTTGACCGGCTCCAACCGCGGTAGACCGTGGGAGAAGTTGCGTCGGGCGACGGCACTGTGGAGATTGACTAACTTGATGTCATATGTGTGTACATGCACCGCGGAATGGAAGGAGCCAAGACAATGTCGCTTGCCGGTGTCATAGTTCTATATATCTGCGGCCCATGTCCCCCACTGGCGCTAACACACGCCGATCTAATTTGGTGGTTGCGGCGGTGGGGGCGGCAGGTTGGGGAGGCAGGATGCGCTGGCGTGGCGACACTCATGGCAACGTGGATCCGCGGGCCGAGCGCCGGGGATGTGCTATGGGGAGCGGTGGTGGATGGATCCGGCCATTTTCGGTGGTGGGTCGGCGGTGGAGTGGCTATGGGTGATGGTGGGACGCCCCGGCGTCGATTGGGCAAAGGGGCCGGTGGGAGGAGAACGAAGTGGGGTGGAGGATTTTACTCCACGGCTGCTCGGCCGAGTACATTTGTGGGGGGCTTGGAGTACAAAAAATCCTCCCCTAACAATTTTTGGGGTTTGGTTAGTTCCTACTTAGCGGAGGAAAACCAGATTTATCGTCCTCTAACCGGTTATTGGGGATCGGTTAGGGATGCTCTAAGTTCTTAGTTGTATTGTTCTCTTTCTAAAATAAAAGGTGTAGTTATTTTTCTCTGCAGTAATTAACGAGTGAAAAGGTGGTACTAGGAACAAAGAATTCGGGAAATAATAGCTTAATTTAATGTAATTACGCCCCTTTCTTTTGATATTTTACTGTTGCATGTAACGACAAGTATGTGTGTAGATACATTCAACTTAAAGCGAGAGAGAAAGATACTAGCTAGAGTGCATGTGTTGACCATGACATTATACATACAACTTTGTAGTCCTTTACACTTGCGTGGTCATGGCAATATTTATCTGTGCAAGGGATATGATAAATTAGTCAGCAATCCATGAAAAGATTGTGTAAATTAAATATCTCCTAAATAACCAATGGTATGTCGATAAGAGCTGGTTGTATACTACTAGTAGGGCCGGCATGTGATCGAATCCCCAAGCACATGAGACCATGATGCAAACTCAAAAAAGTGGCAAGAGCATATAAGCAGCGATGTATGTGAACATATGGACTTTGTTAGAGGCAAAAAGGAGTTCAAACGTTAGGTGTCTTTTTTCTGTTTTCGTGTTGGGTTTTCACTCGTTTTCCCCTAATTAAGCAGGGAATGTTAGGTTTTTTCGTTTGGTTTTCCTTATAAACTGGATCATCTCTATTTTTCTTAATAAAATGCAGTAATAACACCTGCCCCTGCATTGAGGTTCTTCTAGAAAAAAGTGAAGTTCAAATATATAGTTTTAGAAAAAAAATGCACAGATGTGATTTTTGAAATTACGACGCATGGCATGAGTGAACAAAAGTGATTTCTTGTTCTCATCTGATTATCAGCTGCTGGCAGGCGTTCAATGATTGGTGGCCTTATAGGTTGAATATATATCGTGCATGAAAATTGGTGGAGAAGTACACAGCCGGCCGGATCGATGGGACTAGCAGCAGCTAGCTAGCTCTCACAAAACCATATCATGTACGGATGTGGCGCATGATGAGCATATTATCCATGGCATATTGCATGGAAGTTCGCGTCAGATCGACCACATAAATTACAGCATCGATGCAAGCATATATGGACGACTAAAGAAGCTAGGCAAGGCAGAGGGGCATGCATGATGATGAGGAGGAATTATAGCGGGGTGTGCCCAAAAGCACAGTGGGGTGGGGTGGGGGGTGGGGAGGCAAAACTGTCACAAAAAGCGAAAGGGGGGCTGGCTAGCTAGGGTTTTGTTTGGAATGGTCCAAGATCCTGCCTGAATTCTTGGTACGCGCGAGCATGGCCATGGCCAGCATGTTCAGGGGAATCTGATTCTCCCCCTACCTTTGGCTCGCTTTCATGCCAGGCATTTCCTTTGCTTTGCTTTCCCCAATGCCTATGTGGCAGCAGGCCAGTCTCCTCCTCCCCCCTCTTGCTGTGCTTCTTCACACCCCCTCCCCTTTGAGGGATAAAGCTTGGTAGCACATGCACCATTATTGTTAGTTTTCTTCTCTCCAGATCTATGTGTGCTTCTCTCTCGCCTCTCTCTAGGTAGGGGGTCTTTCATTAGCTAGCCATTGGATGGGAGGGGTTGTTTATCTTGGTCCCTCCTCTTTTTTCTTCTGACAGAACATGATCTTTGCTTTTGCTCATGTTTTGATCTTGCCATGCATGGATGGATGCTTGTTTTACCCCAATCGATGTAATCAACGCAGGGGTTACTAGCTACCTAGCTTCACGTCTTTAACGAATCTCCTCGCCTAGAGATTGCTGGGTGGGTACGATTGTCGATATCACCGCCTTTCGACAGCATTGTCCCACCGATGTAACAATGCAATGTGTGTAAATCATTACCGGTATTTGCATCCTCTCGTGTAAATATTCACCTAGACTGCATTCGTTGTTCACTGAAACTGCTACAGTAAAAGAAGATGGCCTAGGAAAGGGAAGTATGTGCACACAACACATGTTTGTTACAATTTGTCTCAAACCTGCAAGGGACCGTGCAATAGACTATATATATGCAATTGCATTTTTTGGTCCCTCGACTTTTGTCAAAGTCTGAGTTTGGTCATTTAACTCTTGCATTGAAGTTCTTGGTCCCGCAAGTGTTATAAGAATGACAGGTTCAACTAGGCTCCACCTTAGAGAGGGTTTTGGTGACAACGTGCCAACAATTTCACACACATGCATGGCAACCCTATTATCATAAAAGCTACTCTCTCCATTCCTAAATATAACTCTTTTTAGTGATTCCACTACGAACTACATACGGATGTATATAGACATATTTTAGAGTGTAGATTCACTCATTTTGTTCCATATGTAGTACATAGTGGAATCTCTAAAAAGACTTATATTTATGAACGGATGAAGTAACAAAATCAAGAAATGATAAAAAGTACCGATAGGAAACTTTTATGTTGCATGTATTTGTAAAATAGATAAATTAAGTTTAAGAAAACTAGTGAAATAGTATTAAAAATCATAGAGTTTCAAAAATATGCATAAAGCAATATTTAGGTAACTAAAAATATTTAAATGAAATGAAAAATGAACTTAAAAGAAAAATCAAGAAATATAAAAAGGCAATGAAAAAGAAAACTTAGGAAAAAGGAGATTAATAAAAATGAAAATATCATAAAAAATTTAAAAAAATGAATTTGAACATTAAGAAAATAAAATAAAGAAAACCTTTAGTAACGACCAACTTTTTTCTCTGTGGGGAAAACTTCCAATCTATTCATCAAACATCATGGCAGTACAAAGAACACTAGAAATAAAAAATACATCCATGTCCGTAGACCACTCAGCAACAACTACAATTACTAGGGCGAGATGAAGGCACACCTCCATCATTGGCCCTTCCTCCCAGGAATTGGGCAAAGCTTATTGTAGTAGAAAGTCGGGAAGTCGCGTGCTAAGACCTCAAAGGACCAGCGCACCAAAACAGCATCGTCCGTTGCCAATGAAGAGAAGCATAGATCGGAAGGATCCAACCTGTATACGCATGAATGTAGATGAACGAAGATCAAATCGACACAGATCCACCAAAGACTAACACTGACCAAATCACGTGAGATTCGCCGGAGGCACACATCCACAGGCCCTCCAACAACGCGAGAAGCACCTCTGGGATGGGGCTTAGGCGGGGAGAACCTTATTCCATCAGGGAATCATCGTCGGCTGTCCTTCCTGAGGAAGAACACAAACTAACAGACTAAAAAACACCTAAAAATGAAGCATGAGTCCTCCCATCGGCAAGGTCTAGGATCCATCGTGCCTTCATGGCCCTAAGGCCACACGAGACGAGACAGATTGGTACATGTTTTTTCTATAATTACCAAACTCTAATGTTTTTCAGAATGATTCTAGTTGGTATGGATTTTTTTCTAAAAATTAAAATTATTTGTATTTTATTGACATATTTATGTATTCATGCGAAACATGGATCTTAGTTACAGTTGGGAGACAAAAACAACACTTTTATTAAGATTGCTTAATGAACGTCGTGTAGCCTACTGTGATGCCTCCACAGACCTATCTGTTCCATACAGCTTGTGGACCACTGTCGCAAAGCTATTTCTTCTTGTGGCCATTGCGACCACCAGTCATGCAAAATGGCATAATGTTGACAAACCCAATGACCAACTTAAGAGCCACAATCAAAACCAATATTTGAGGCCTTCATCCTTTCTTTAAAGCACGGAGCCCATTCTCAAAGATATAGTAGGAGTACTGCAAATGCTAGCAGGAGATGGTGCGCTTATTATTTTCATTGACAAAACAATATTACATGATACCTCTTGTTTTCTATTATAGAGATTATCTTTGGTTAAAATGAAACCCTTAAATGGGGATTTAATATAAAAAATTATGTCGACAACCCCACACATCACTAAGGCCAACTCCACCGCACGACCCCAAACGGACGTCCGGTTTGGCCGGATTTTGTCCCTTTGGGGCGCCGATGGGTTCGCCCGTGTCCGGCTTTGTCAGATGGGTCGTGCGTGCGCCCACCGCGCGACCGCACCCCAAATCGTGTCCGGGGTGGACGTGAATAAAAAAATATAAAAACTAGAATGAAATAACTAAAAAGGTAAATAAACGCATTTAAAAAAAACATAAAACATATATAGGGGTCGGCCACAAAATGGCCCAGTTTTCACGACCACTTAAAAGGCCCAGTTTCATAATTAACATATAAAAAAAACGCCTCCCGCGCGCTCCTGCCGCGCCTGTCGGTGCCGTGGCCGTCCCCGTCTTCACTGGCCGCCGGTGTCGTTATCGTCGCTGACGAGGTCGACGTTGGCCGGCGGCGTCCACAGGTGGGGCGGCAGTGTGTGGTAGACGGGGGCGGGATGGACGGCCAGAGGTGCCTGCACCACCTCCTCCCGCGGCGACGCCTCCCGCTCCGGTGACCGCGGCAGAGTGGGGCACCAGTTCACGCCCAGGCCGGCGGCCATCTCCGGCGCAGTGCAGGACCAGCCCCACCCCTGGCCCAGCAGCTTCTCGAACGCCGCGGGTCCTCCTCCATCGGCTCCTCCGTGACGGCCGCCGTGACAGCCGCCAGCTGCAGCTCCGGGAAGGCGACGTCCCCGGCGGCAGAGAGGGCCATGGCCTCCTCCAGGCCGTCCCACTGCCGCTCGTCGTGCGTGTTCATGGAGTCGTCCATGACACGCTGCAGGAGACGGGCCTCCTCCTCCGCTGTCATGCGAGGAGGGGGAGGGGGCGACGGAGACGGGGAAGGCGACGGCATGGGCATCAGGCCGCGCATCCGCGTACGCCCGCGCGGCTCTGCACGTGGCCTCCGCGGCCCCGACACGGTGCCGGTGAAGTAGGACACGCGGCGCGTGTCGTGCTCGTCTTGGAGCCATGTGTCCCAGAGCGGGGAGTCGGGGGCATACCTGTGGTCGTAGTACAGGTCGTCGGGGAGGAGGCGGCGGCGGCGGTTGATCTCATCGCGCCGTGCACGGCCGGTCGCCGGCACTGGCGGGATGGGGACCCGGTCCGCAGAGAGGTGCCACCCGTTGGGGAGGTTGCCGTCTCCCAGTACCTCCGGCACACGTCCGCACAGATGTACTGCTGGTCGCGCTCGCCGGATGGCCTAGGGGCAATGGTGAAGGGGGCCGACGCGGGGGCTCGACGGGAGGAGTGCGGCGGTGACGCGGGGGCTCGACGGGAGGAGCGCGGCGGTGACGCAGGCTCCTTCTTTTTCACGGATCCGCGGCGGCCGCCGGAGGAGGAGCCGGACTCCCGGTCGTGCTTCCCCTTGCGGTTCCACAGACTCATGGCTGCGGCCGGCTGGCGAGCTCGAGGGCGGGGAGTGGCTAGGGTTTGGGCGTGTCGACTTTCGAGGGGGCAGAGAGGGGCCGGCGTGGGGATGAGGACGACGACCGGTCCACGGGTCCCATTTAAGAAGGACGCCGACCCGTCGCTGTGCGGATGACAGGTGGGGCCTCCCGCCCGTGCGCATTTATGTTGGCGGGTGGGAGGTAGGTGGCCGCCTGCCACGCGGCCCCGACGCGGACGTTCGAAGCGTTCGTTCGCTGTCCGCCGCGACCCATAACCAGGCGCAAGTTTGCGCTCGAAATGGGTCGGCACGGACACAAAACGGACCAGATGGGTCCGGACCATCGCGCGCTGGACCGTCCCATTTGTCCCTTTTATCCCAAACGGACGGGGCCGGACAAGATAGGGTCGCGTGGTAGAGTTGGCCTAACAAGTATGCCCTCAAGCCATCCGTCAATAAAACATGACGAGACCTTTTATCAGGAGTGAAATGTTAATATTGTCCCAACATCGAAAGTAGGGGCTAGGTGAGGGAATTCCGTCTTTTGTCAAGGAGCAAAGACAAGACATTTTGTCAACATAACCCGGCCCCTGGATAGATAACTCAAGATATTTTTTTAAGATGTATATTTTAATTTTTCAAGTGCATACTGAATATGGGGATCCCTAGCTAGAGCTTCTATGACAATTGTTTCTTGAACAGTTTTTGTAGAAATCACATGAACCCCACCCTTGATCTCTTCCACGATGCTTGACATTTTTCGTCCGTCTCGTGGTGCGGTTTCTGATGACCATGTACTCTACTATGTACTCACTTTGGTTTGTCAGACAGCAATGGATTCCTGGGTCATCAATTTGGCCAACGAGACATAACTTCCACTCTGTTGGTGAAATTCATGATCTAGAAATATGTGTTTGCTTTATAAACTGAAAAGGAGGAGTACTCGATATTGTTCCTGTTCCGGCACTGCATTTTTTCAGTTCCCATGTGGTCAAACGACTGGCATCATGCATGATTTGTGTTCCCCTCTGCCCCTCCCAAAGATCACTCGGTCGGCGAGAAGGGTCGATGATATATGTAAAGCAAATGTACATTATTCCGCTTATTAAATTCTGCAGTGTGGCTGCGGAGATCACCTCATGTATGTTTACATACGCGCGGTCCTAACGTCATAATATACTACTCCCTCCGTCCGTATTTACATATCGTCTTTTATGTCGTCGTGATTTAACTTTCACCGATGATTTCATCAAGTAATACCTGAGCTGCGTGGCATAGATATCACAGCATTGGAAACTATTTTCCCATACAAATTCCAATCGTATAATTTAGGTGGCATATAACCCACGATTTTCGGTGAAAACGAAAGTCAAACTTAAACCTCGAAACGGGTTCCATGCGGACCAGGATGACGGAAGGAGCAGTACATTCCATTCCATGCTCAATGTAGCATCATGATATTCCAGTGCTGATGCCTAGCTAGCTGCGTCGATCGTAGTATATCCTGCAGTCGCAGTCGCAGATATAATCATCAGGTTCCTTCGTATATTCTACAGATCCTTCCTTTTTCTCTCTCTGTTTGTTTGTGCTGCGATAGCACAGATGGTACATGCAGGATTTTGTGCTGGCAAGAAACATTAGTGAGGTCACAAGTCACATCGAACCTTGTCAGTTCTGGCACTGCATCTGCATGCATGTTCAGGGTTCAGAAACCGATCTTTTCACATGTTGCAGACGGTTTCTGCATTCCCATCACAATCTAAGGGTTAAATTTGTCTAATTTCAAAATCTAAGAAGTGAAACTACAATCAATAGTTGAGCACTGAGGTTAAGTCCGTGCTATATATGGTGGTTACGGGCAATGTACGTAGCCGGTCATATACGTGCCTTGGCATGTGGGTGAAGTAAGTATGTACGGCAGTGGCTTCTTTTTATCGATAAAGGACGCTTTTACAAACACAAAATATAGCATCGAGTTGATACAATTTATGATGAACAACACCTGACCTCTGCATAGCTAAGATGCCCACAGCCAAAAACAAGCAGTCTGAAAACATAAAAATATAAAAAATGGACATATCGGCACCAATAGAGTCAAATAGGACCGTCACTGTGCCTATATCGAAAGAGGTGATGGACCAATACCGAGGTTATGATACAGCCCATGTTGAGAAAAAACTTCCATGGCTCCAATCGCGTACACACCGCCTTGAACGGCGGTTGATACTACGTATGGTGAAGCATAGACCACATGGCGAGTGCGTACAACAAAAAATAATCTGCAAAGGAGAAGACTTTTTGTCCTTAAAAACCAAATCAATTCTACATAAAACAAAAAGACGATAATAAGACATACGCTCCCAGCTTTAATACTAGCGTTTTGAACCTATAATAGAACGGCAGTGTCTTCTTGCCTTCTTCCCATCGATGATACGAAACAGTCACCATGCACGTACGAGTACGAGTACGAGACAGCAACCAAATGCAAGTTTCAGAGTCCAGTATGCTATATACGCACGTATTGCCTGAGTACACTAACAAAGCTACACAAATGTTTTTCTTTTTTGAAAAGAAGAAGAGCCCCCAAGGACCCAGACCACCATTTACGACACTTTCAATGCTGACCCGCAAACCGGACACCACATCCATCCACGGACCAACGGGACCAGTTCGCGGACATGGATGAGAGAGCCCGCCATTCAAAGCTAGCTGAATACTATCCGCCAATAAAGTAAATTTGAAATTCAATCTGTCTGAGGAAGCACGCACTGGATCTCACAGAAGCGGTGGACCACATCATTGCATGATCACAACAAAAAAGAAACAAGTATGCTTAGTTTAATCTGACACCGTGCAATCCGAGCTTCCATGTTGAATCTGCCGTCGCCACCATCTCGTGCCGTCGCCACCATCTCGGCTGACTCCACGCCGCCATCTCCAACTCATTGTCCTGCCACGGCAACATCTTAAAATGGTCGGCTTGCACAATAATCCCTGCGTCCTCATCCAAATCTTCCATCTTCAAGGTGACCATATACTCTAGCTTATTTTCATAACTCATTTAGTATTTTATGTACTCTAGCTTATTGCATAATGGTTGTTGGAAAATTAGTTGTTCATAAAGAGAACAATTGTTTTTCGTTGTTATGCATAGTGAAGGGCCATACTCTTCTTATGTAGTATGTATGTTATGAATATTGACAATAAAGTTAAACATCCATAACATTGGCGCTAAATATCATAGCACTAAATGAATATCACAAGTGTGTGTGGCACTGCATTTAGTCACATTGTAGAAATACACATGGAGAAATTCTATAGTTATGAATTTTAGAGTCATCTGATTACGTATCATCTGACACTTGCAACTTTCACCCAAATAGTGGAGTAACTAAAATATCGTTCACAGGCATTAAGAACAAGCAACAAAAATGATAGAAGCATACATGCTGATGTGTGTAATTCAGTAAGAGTTGTTGCAAGCGGTGGATTTATCTCTCTTCAAGAATGACTCAAGTAGATATATGGATATTTTCTTGTTGGGACATAAGTCTAGATCCTTTAAAATAGTTAAACAGATTTTCAGAATGAAGTAGAAATTATTATAACAAGATAAATTATGTTTTTGCAATTAGACTTAAAAGAGGAAATTTTGAGTTACAAGTTTAATGAATATATGATGATTTGTGAAAAGAGCTTCATAACTTGCACCTCCCGGAACAACACTAAGAGTGGAGTATCCAAGAAGATGTAATCAAATCATGTGTGACATGGTGAGATCAAAGACAACACAAATCAATTTTCCATTATACTTTTAAAATCTCATGCTTTAGAGACTGCGACATTTATAGAGTGCTATCAAATCTATTGAAAAATGACGCCATATAAGGTATGGTATGGTATGTCAAACAAATTTTGTCTTTTCTTAACATTTGGGAATATGAGACATATGTAAAAGTGTTTACAAGCTGATGAGCTCATATCCCAAGCCGGATTAGTGCTACTTTGTTAGTTATCCCAAAAGATCGTATTCCTCTATCACTACACCGAGGCAAAGTATTTTTCCATGGAGTGCGGTGAATTTAGAAGAAAACGTTTCTTGCAAAAGAATGAGTGGGAGAACAGTGCAACTCGACGAGATGACAGAATCTTCGTAACCAGGTCAAAGGGAAGAAGCGTTAGAAGTATTTCCAAAATTTCCTACTTTGACTGATACGCAAGCCTCTACATGAGACATAGACTTCGATCGAATTTGCAGCTAAGCCACATGGGTTAGGCAAAACTCATACAGGCCCGTGTGGTATGCAGACGACAAAAAATTAATCTATGTACACAAAGAAGCATTGATGAGACCTGACTCTGAATTGGTTATATGTCAAAGTAATCCAAGACATTATCCATACATGATTCCAGTTTAGAACTTGATGAACCCTCCAAAAGACTTAGAGTTTCACGTATAATAAAGGATATGTAAACCGACAAAGATAAAAATATTTTATAAAGTTGGACTTGTTTCGAATAGTATATGACAAGTTCACATACTTGACTACGAGAAGATTGTCTCATTGTATTGATGCTTAAAGTCACTTTCGGTTAAACTAGCAATTACTACATATTTGAATTATGAGATAAAAAATATGGACGCCAAAAGGATTTTCATGAGAAGGAAGTAGATCCAAGGAGATTTTGTCGATCAAGAGGATGCTAATAACTGTGCAAACTTCAAAGATCCAAGAGTGGACTGAAGCAATCATAATGGAGTTGGACTCTTCGTTCTGATGAATGCTCAAGGAGTTGGATTTCATTGGGGAAAGTGAAGATACTTGTGTTTACAAGAAATTAAGTGGGATCTTAATAATATTTTTCCCTTTTCGAGGAGCACATATGTGTGCCTCCACGAGATGCAAATACGCACTTCTCGTGGAAGCACAAAAATAAAATCTTGGAACAAATTCCCTTTCCGAAGAAGCACAATCGTGCTTCTCGTGGAAGCAAATATGTGCCTCCTTGGGAAGCACATATTTACAAAAAGAAAAGTAACTTCACTAAGAAAGATTGCTTCTCGCAGAAGAAAGTCTATGCCTCCACGAGAATCAAATATGTGTTTTGTGAAGAAGAAGAAAATTTGCGTAGAAAAATCATTTTCCCTTTCCGACAAGCACAAATTTCTGAGAAGCAAATATGTGCCTCCACGAAAAGGAAATCCGTGCTTCTTGTGAAAGCAAAAAAGAAAATCACAAGAAAAACATTTCATGATTTTTTTTCTCTCCAAAACATAGGGAAAAACCAGGCGCAAACTGAAAATCCAAAAAAGAAAAAAGAAAAATCGCATCAAAAATACAAAAACATGTATGGAAAAGTAAAAACCGGAAAATAGAGGGACCGCCTACCACGTGACATGTGAGAGCGGTTGGACATACCACTTGGTGCACTCTCAGCCCACAAAAGTGATGCTTGCGGGGCTCCCGCATGGGGTACCCCTTGACTACTTGCTCTCACCAAATAAGAGGACCTGCTCGGAACGGCGAACTGTCCGTAATTTGCTCAGGCTGGGATGCCAGCGGCAAAGGCATGGGCCAGCCCACTTGCAAAGCCATACGCAGTGCGGTCAACCTGTTAGACATTACTGGTCCCTTTTTTTTCTTAAATTTTGTTTTCATTTTTTCTTTGTCATTTTTTTCTTCTTGTTTCTTATTTCTTTCTTCATTTGCTTTAGTTTTTCTTTTTTTTTGCATAAAAAATTTCAAAAAATGTTCACAACTTACAAAACATGTTCATGTTCTTCATAATATGTTTGAGAGTTTCAAAAACGGTTCCCGTAAAAAAATTCTCAGCAGATTTAAAAAACGTGCATGTTTTAAAAATATTGTTCCAAACTTTATGAAAACTTTCCAGTTTATAAAAAATACTCAGGTTTAGTTTTCTTATTTTGTATTTAAAAATATGTGCATGTTTTGGAAAAATATTGGGAGTGTCAATAAATGTTCCTTAAATGTGCGTGTTGTCAATAAATGTTGCATTTGGAAAAAAATATTTGACTATTTTAAATATATTATCCGAAATTATGAATGTGCTTTCTTAAAAAAAATCTAGAGGGATCAAACCCAAGATTTTCCACAGCCAGTAGTTCAGAATTTGAAGAAATCTTTGGACCTGCACTTGAACTCGTTCTTAAAGGGTTTTCTGAACGCGATCACTTGTCAAATATCTTTGGATTTGATTCCTCCAAGAATTATTTTTGGGCATGGACAATTTTCAAATTTGTGAATACATTTTGAAAGATTTATAATATTTTCTATAAAATGTAAACCTTTTTAAAGATCATGATCTTTATAAGAGGAAACATTTATTGTAACTTCGGATTATTTTTGAAAATGTGAACATTTTTCAGAATCCGGATATTTCTTGAAATTCCAAAAAAAAATTGGAAACATAAATAATTTAATAATACATGAAAATATTGAAACTCCGGAGCACTATTTGGAAACCACAAACATTTTCTTAATTTTGGGAACAATTTTTAAAAGGGAACACTTTTTGATATCCCCAATCAAAATTTGCGGAACAGGAATATTATTTTGAAATATGCGACCTAATTTTGAAACTACTGGCCAAATTTTGAAAACAAGTGTGTTTTATGAAATTTTGAACATTTTAACACGAATGTTTTGCAATTGCTCATTTGCAAAAATGCAATCATTTCTTGAAAAAGAAAAACCAACTTGAAGAAAAGGAAAACAAAAAAGAAACAAAAAATGAAAATGAAACAAAAACAAAAACGAAAAATAAGAAAAAAAACCAATAAACAAAAAAAGTAAAAAAAGAGAAAAAACAAACAAAAGAGACCGTAAAACAAACAAAAAAAGGGTTCGGGGAAGAAGTTTTTTAGGGTTGTTTGCGGGGCCTTACTGTGTGTATACCGTTCAATCCGCCGGGACTTGTCTCGCTTGCATCGCTATCTTTGCGAGTTACCGATAGTTCTCCGCAGCGAGCGGCAAGGCAAATGTCGTGAATGCTATATATCGTGTCAATCGAGTTGCAAGGTTCTCTCGCTTAAGCATCTACAATGATGGGCCGGCCCATTAGCTTATATACATAAAAGAGAAAATGAGCAGGGAAAACACAAGTACCGGGATTTGATCATGGGACCTCCATGATAACAGACAGCGCATATAAATTAGGTTAGCTATCATTTCCTGTCAAACAGTAGGGGCCCGACATATGAGAGTAAATAACATCCGGTTTTTCAATGGTTTTCCTGGTTTTTTTCAACAGGTTTTTTCTATCTTTCCTTTCTCCATTTTCTTCTGTTTTATTCAGTTTTCTTCAGGTGAATTTTATTTTGTCGTTTCTTTTCTTTTATCTTGTCTGTTTTTATCATTTTCTTCATTTGTTTCTTTGGTTTTATTTCCATTTTCTTTGTTCTTTTGGTTTATTTTGTTTCTTTTACGGTTTCAATTTTTTGTTTTTCGGTGTTTCTTTTGATTTTCATTCTACATTTTTTTATATGTGAACAACATTTTTTCTAATACAAGTTTACCATTTTTCCAATACAAATTTAACATTTTTCAATACATAGTCTACATCTTTTGTATACACATCTTAACATTTTTCAATGCTTGATTAACATTTTTTAAATACAACATTATTTTTTTCCAATGCACAAAAAAAATCAAATGATTGACTAACATTTTCTAATTCCTGGTCAACATTTTTTTATGCACATTTGAACATTTGTCAGATACTTGATTAACATTTTTTAAAATGCAATAGTAACATATTTTTAAAACATGGTGAATAGTTTCTCTAAAGGCATTGTACCTTTTTCAAATACTTGTTTCAAAAATTTCAAATTCTTGATTAACACAAACTCACACACACACACACACACACACACACACACACACACACACACACACACACACACACACACACAGAGACACAGACACACACACACACACATGAGAAAATTTGTTTCATTGTTTTTATAATATGTGGTCAATGTTCTATCTATACACATTTATCCTTTTCCAAGTGCTTGCTTAACATTTTTCAAACACTTGTCAACATTTTTTCGGATACTTGATATTTTTTAAATAGATATAAAAAAGTATTTTGTGCACAGTTTATTTTTATATACATTTTTTGGATATGTGATAAATATTTTCTCCATACATATTTAACATTTTTfvATAATGCTTGGTCAACATTTTCAAATGTTTTAATGTAGAGTGGTTTTGGTAATATACTTATTTTTAATATTTGAAAGTATAAAAAAGCAAAAGAAGAAAGCAAAAACTAAAAACGTGAAAAAAAGAGAAACAGATGACGTGGCTGTTGGTTCCCTCGCGCCAGGGCGGCCCTACTGGTGGCTACCTTCAGCGAGACATAGGGGCGCCCGTTTACGATGGTGGCATGCTCGCCTACTTATTACACCCTTCATTGCCGGCCCACTACAGTCTGTAGGTTTCTCTATAGTCTTCACGTAATTTCATAATAATATTGATATGTTAAAAACATTCATGCCATAAAAATTGTTCAGATGTGAAAAACAATTTCATGACTTTAATCAATATTAGTATCATTCAAAGATGCTTATGACATTTTAAACTGATTTTATCATGTATATTAAGAAATGATGATCATGCATTAAAACAAATTCATATTGTATTTTAAAATTACTTTGCTCTCTATATGAATATACAATGTTTTTTTTGAAAATTATTCTTAATATATTTAAAAAATGGACGCTGTGTTTTAAAAATTATTCTTCATGTATATAGAAAGTGTTCATAACACATAAAGATGCTACCCGTTTAAAGATATTTCATGAAATAATTTAGAAATATTCAATTTGTGTGCATTTATATTTTTATCTTTTTGAAAAAAAACTTATGTGCATTTAACATTTGTTCATGCATTCTATTGTTCATTATTTTTTGATATAGCTCACCATTCATTCCAAATGGTAAATGTTCATGTTTTCAGATAAATGTTAATACTGTATTTGATAATGTTTAGCATGTATCTTCAAAATGTTCAACATATATACGATTTTTGTTTCAACGTTTATTTAAAAGGTATGATGTATTTGAAAACCAAAAACACAAAACTGGTAAAAACCAATAAATGAACGTTTAAGAAATGAAAGAACTGATTCTGACTTTCCCAAAACCAATAAGAACTGTACTTAGAAGCTTGCAAAATTAATTCTATCTGGTTAACACATCAAACGAATTATTGGAGCGATGCGATACTAAGCGAAATATAGTACTGCCACCTTTTTTTTCTGAGAAACTGTACTGCCGCCCATTTCCAACATCACAGTGATCCGTAATGGGCCATGCCATGTACCAGGTTTTGTAAGCATACACCAGTTTCGGGATGCTTCCAGGTGGCTTTTTCTATGTCAGTTTCATGCTTTTTTGGTCAATTTTTTATTTTTACTATTTTTTGTTTGCCCATCCTCTTTCTTTATTACTTTGATTTTAAATCATTTTTTAATAATTTCGTGAACCATGTTGAAATTCATGAACATTTCTTTGAAAGCAGTAATAATTTTTGAATTCATAAATACCCCCTCACTTTCTTTTTACTCTGCTTACAAAATTTGTTTGAAATCAAACTACGTAAAGTTTAAACAAATTTATCCTAAAAAATATCAACTTTTACAATGATGCATCTAATGATATTGATCAATATGAAAATTCTTTTCATGATGCATCTAATGATATTGATTTCATATTGTGAATGTTGATTTTTTTCCTTATATAGTTGGTCAAACTTTTAAAATTTGACTTTAGGAAATTTTTGAATGCATACTAAAAGAAACGGAGGGAGTACTTTCTTGAATTTGTTACTATTTTTAAATTAGCAAACATTCTTTTGAATTCACCGACATCCTTCTCTATTAGTGAATATGTTTAACTTTTTTTAATATTTTCAAAATAAGTGACCACTTTTATATCCGTGATATTTTGAAAATCTATGAAGTTTTAGAAATTTCTTAACAAGTTCTAAAATAGATGAATATTTATTATATTCATATAATAATTAAATTTATAAAAATCAAATCATTTGACATATTTTATAGTATTGAACATTTTGTAATAATAGTGAAGACACAGAGCTATTTGTTCCATGCGGTGAACTGGTTTCCTAGTTTTTTACGGGTTGAACCAGTTCATCCATTAATATGGAAATGTACACCTACGGAAGTAAAGCGAACACTATGTCTCCAAATGGGCCGGTGTGAGTCCTCTCACGCTCCTATACGGAGAATTCCCCACTTAAAGCAGGCAATAGGACCGACGCCTCCTATTTCAAGCGGTCTCCGTCTATTTGTCGTCGAATCTCACATAGGAATGGGATATTGTCCAGCCCATGAGGGCCTTCGTGGAGTGCTCAACTGAAGCGATTTTTTACCTTTGGTTTGCCTATGAAAAAAGTGACTGCTTCTTCAGATTTGCGCTCGTCGGAGAACTCAATTGCTTTCAATTGCGTCCTTTGGGGTCCTGTTTGCAAATGACAAGGATTTGCTTAAAATTTCAATTTACTTTAGAGTCCTGATGGCTCTATTTTGTATGCATTTTTACAGCTCATTTGTTCCATATTATCTCCATATATTCCTACTAAACCAAGTACATGTCCATTATGCATGGTTTTCGGTTTTCACTCTTTCCAGTATCACTATTGCATAGTTTGTGTTTTATTTACTTTTATTAGTTTCCTTAGTTTCGTTATGTCTATAGGTGTTTTATATCAACCATGGACCAAGCATGATGAAACAGCAAAGGTTGGGTTCAGTACAGAGGACTTCCAGGCACCAGACTTAGGTACTTCAGAGTGTAAAAATGACATTTTTTCAAAGTGCTCAAAATCAAGATGTAATACCACAGGTGCATCGACTTCGTTCATACTCCCTCCGTCCGAAAAAGATTGTCCCTCAAATGGATGTATCTAGAACTAACTTGGTGCTAGATACATCCATTTGATGGACAAGCTTGGGACAAGCTTTTTTGGATGGAGGAAGTACGATTCTAATGAGCCCAAGAACATCAAAATCCGAGTTCGGAATGACAATCATTCTCCGAAGAAGACCAGAACGTTCGATGAGGCACGGTACAACTATTCCATGCCCAGAAAAGCCCAAAATGGTTCAGACCAGATGGGATTCTTGGTGGATTTCATTCCTATTTTTTTCCTTCGACATCTTTGGCCATCAAAGGAAGTTTTGGCAGAGGATTTGGCTCATTTAGAGCCCTTGAACCAAAGGAGAAAAGATACATCTACGGATGAGGCTTTCATAGAGCCCTTATATACACATCTTCAAGCCTAGTCGCCTCCATCATCCATCATACATCCAAGATCAACCTCCATCACGTCTATTGCTACTCCAACACCACCTCGATAGAAGAAGGAACGAGATCAAGAGAATGAAAAGAAGGCTCTAGCACACACCGCATCGGCCTTCGGGGCGGTGCTTTCTCCACCATGCCACCGCTGACATCACTATCTCTACCACGCCATCACCATTGGTGCCGCCTCCACCAACGCCACCATGTGTTCTCCATTCCCCTACTCGATGCCGACTTGTAACTTGACTCTCTCCTCTATGTTACCCGACTTTATGTTTGAGCAGTTGTACTTGTTCTTGGGTATATGGATGAACTCTTTATGATTAGAATTAAATTCAATTAGGCGAGCCAATATCTTTTGTTTATGTTGGAGTAGCTTTCATGTGTGTTGTGAGGAGTACGCACACAACATTGTCGCCTTGATTGGCTGTCAACCATATTACTGCTGTTGTTCTGGGTTAGGATGTTGAAGTAATTTGAGGTGCCGGTAAAAAGCCTCTACCTTCACCTTGTCCAATCCGTAGGGGAATAATAGAGAACCCTTGGTGAGGCCGCGTCTGTATGCGAAACCACCCACTTAAGCACATAAAGAGTAGTTTAGGGACAATTCATAGACCTATGCTCCGCACTTGTTGCATGGATCATATTAGTAGTGATGACTACGGAACACTCGAGTGACCTTATTGCTCAAGCACTGCCTATACTAACTTAGCATTTTTCTTATTCCTATTTTTACCTTCAGTTCGTATTCTAGGTAAAGTCATTTTTATTCCGTTTCTGCAGCGATTTATAGAATAGGCTATTTCCAAACTCCGGTTTGGGCGTGTACCGAATTTCATCGGTGAAAATGTAGTTACAGGGTTAGTAGTGCCTTACTTTTCTCTCCTTGCGGGTTCGACACTCTATTTGCCATGGAAATGCTACAACACCCTATGCTCTTGCTGTACATCAAGTCCTTTCGATACTGATGTATAACTTTTGATATTATAAACACCGGTTTCGAGGATCTTCGTGTCCCTTGTCAAAAAAAAATACATGGATTTGTACAAACTATAGAGTACGCATGTTGGCACGGCAAACTATTGTCCCACCTCACCAACCAAGAGCTAAGATGGAATTGGAGAACATACTTCTAGTTGATATCCATACAAGTGTAGAAGTATCTATTTGTTTAACAAGCAGGAGCTAGGATGGGATTGGTCTAGAAGTATCTATTTACAAATGGAGTACTCTCAAAACATTTGGACATTTATCTATAAGATGCATACATTACTCTTAAATACAGTAGCAAGTTTAATCGACATACGGTGACCTGCAAACAACATAATGTGGTAGTATTGGTTTATTGAGATTTACAAAGATATCCATTGTAACAACATAGTGGCTTAAACGTCCGAAACAAAATAAAAAACATTCTTTGTGACAAAATACAATTTATTTTTAGATCTGTAAAATATGTTTCTACTGATAAACATTAGAAGAGTATGAAAAATAAGTACCTAAGATGCAGGAGCTTGTGTAAATTGTTCATTTTTCTTATTATTTATACAATAATATTTAGGCTGGTATGTTGAATAAATAATATCAATAGAAACAATATATATCTTTACATCAAGATGAGTTATCACGAAGTAAAAAAGTTCAATATAAATAGGTGGTACTCCCTTTATTCTGGTAGATAAGCCCAGTTTTTACCGATGCATTAGCTGGAAATAATGACATCCGTTAAATCGGTTTTATTAATTGTGCTTATCTATTAATTTTCCTAAAATTAACTATTTTACATAAATATTGTGACCAACAAAACATTCAAGCGTCACATCTTGATTTGACATCTCCGGTATGTATATACTTTATCAAACAATTAGCATGTAAGTAAAAGGAAATAAAATTAAAAAATGTCATAAACACAAAATATTCTAAATATCTTTTCAAAATCCAAAATCATTTGAAATAAACATAAAATACTCTCAAAATAATATTAAAAAATATTCATAATTTATTTATGAGTTTTTACAAATTTCTCAATTTAATACTACTTCTAAATTTATTCACCTTTTTATATTTTCCTAGATTTTTTCATATTATGTTCTATTTTGTGCTTGTCTTCTTGTTTGCAAAACGAGTGTCGCGTCACTAGTCATGCCACTTTCACTATGTCGGTGGTCAAATCATCAATCTTTATATATATTCGAGTATATAATCACAAATAAACTCAACCAATAGTTACTTGATAAAAAAGAAGTTTAGTCCCAAATTTGTCGTGGTCTCTTTTTCCTTTAGGACATCCACATCTCAACATGAAAATTGTACTAAGGTCCAACATTATAGGGCCTCCATGGAACCGTGGGATTACAAATAACACAGGAATTTTGAGATTAAATGTTTGCTTCATTGGAAGAAGACACATGCATGGTGCATTCAAAATTGAAGTGAACTTCCCCATGAGATGTTGGTTTCCTCCAAACTACTAGGAAAAATTTCAAAGCTGAGGTTTTATTTTCGTGTAATCCAAGTATCCAAAGTGTATTTTTTTTATCTTTTGCTTAGGACTGCAATCAACCATTGTTCCCGCAAAAAACCTTATTGGTTTTTAAGGTTGGCTGAACTTGGAGTGTCAAAACAAAGTTAGTGAAACAAAATTACTAAAACACTAGTAGTTTATATCATTTAACTCTATGGGCTGGTATTGACTATTATGTGGCTGCAAATCAACTATAGCAAGCTAGCTAATCAGTACGACATTAATTAATTATTGCAACTGTAGCTAGCAACACTGCATGCATCAGCAAGGTAGCATGGGTAGCCGTCTCAGCCCCATCAGCACTCACCATGGAAAAAGCAAAAGTGTGTAAATAAAGGAAAACAACAACAAGATGATGTCCTCTCCCTCCTCCAAATCGCCACAAGCTAGAGAGAGAGAGAGAGAGAGAGAGAGAGAGAGAGAGAGCCTTCTCTCTCATCTCTTGGTTGTGCTGCTACCTGGCGCTCTCTCCTTTTTCTCTCTCCTCTTGGGTAGCTCTCGCTCCCTCTCAAAGCAGTCAAGAGCTAGACCCTCCTGTCTCCTCTAGCTTCCATTCCATTCCTTTCCTTGGTACTAGTACTCTGATTCCCTTTGATTTCCCCAGCTGCCGCAGCTGCCAAGTCTCTTCCTCCCACTATCTCTTCTCTCCAACCTCCAGCCCTGCCAGCCGCCCAAACACCTCTCTCCTCTCCCAACAACTCTCTCTGGAAGTCTAGATCGCCGGCCATGATCTTCCCTCCTGCCTTCCTCGACTCATCAAGCTGCTGGAACACCAACCACAACCAGCTTCAGGTATGCATCCTTGCGGTCAATTAATTCTTCTCGCAAGATTTTGTTCACGCAAGAAAAAGAGAGAGAGAGAGAGAGAGAGAATATGTTCTAGCTAAGCTAGGGTTTGCTGATGGCAGATATACATCCTCTGCTGATTGCTGCACTATGTATCTTGGAATATACTCCATATACACATCTTGGCTGACGCTTAATTCCTGACCACTTAATTTGCAGCTGCAGCAAATCGGCAGTAACACTCATATCACTACTACTCCTTCACCTGCTGGCCATGGTCCTGGAGACGGAGGAGGCGGAAACAACAACAATCATGGTCAGCAGGAAGGATTAATGGCCACGGCCGGGGCGGGAGGAGGTGGTGGTGATGGTGGTGGCGGCGGCGGTGGGGATGGTGACAGCGCCAGCGGCGGGAACAACAAGCCGATGTCGATGTCGGAGCGGGCGCGGCTGGCGCGGGTGCCACAGCCGGAGCCGGGGCTCAACTGCCCGCGCTGCGATTCCACCAACACCAAGTTCTGCTACTTCAACAACTACTCCCTCACCCAGCCCCGCCACTTCTGCCGGGCCTGCCGCCGCTACTGGACCCGCGGCGGCGCGCTCCGCAACGTCCCCGTCGGCGGAGGGTACCGTCGCCACGCCAAGCGCAGCACCAAGCCCAAGGCCGGGTCGGCTGGATCCGGAACTGCCGCGGCAGGGACGTCGTCTGCGACGTCGACGACGCCCAGCACCACTGCTTGCACCACCGGCACAGCTGCCACTGCGCCGCCCGCTCTGCAGTACTCCATGTTCGGCAGCGCGCCGCCGCACAGCAGCCGGTTCGCCGATAGCTTCGACCCCGCGAGCCTCGGCCTCAGCTTCCCCGCCAGGCTGCTCTTCCCCGACAATGGCGCCTACGCTGCCGACGGTGGCGCGCAGCAGCACCACCACCACCAGGGGAACGGGAACGGCATGGAGCAGTGGGCGGCTGCGCACATGCAGAGCTTCCCGTTCCTGCACGCCATGGACCACCAGATGTCCGGGAATCCTCAATCAGCTTCGGCAATGCCAACCACAATGGCGGCGATGCAGGGCATGTTCCACCTCGGGCTACAGAGCGGCGGCGGCGGCGGTAATGGCGACGATGGGGGAAACCACCAGTTCCACCACCAGCCGGCCAAGAGGGACTACAACCAGCAGCAGCAGCAGGATTACCCAAGCAGCAGGGGCATGTACGGGGACGTGGTCAATGGCAATGGCGGCGGCTTCAATTTCTATTCCAGCACTAGCAATGCAGCTGGTAATTAGCTAGCTAGATCTAGCTAGCTTTGTTCTTGCAAACCTAGGTTGATGCATGGTGAAATGGGGCCGGGGATGTATAAATTCTTCATCACTACAATATGTGTTACAAGAACTCGATCCATCTCGTCGATCGAGATCTCTAGAGGAGGGGGGATATCAATGCATACACAAGAATGTTAACCTTTTGTCTGTTGAGTGTTTGATCACTATGGCCTTAATTTGTAGTACTATTGTTTAATTGTTGTTTCATATACTTAGCAGCTAAGTGTAGTGTGTTGGTAGCCCCTCCACATGCATGCTCTCTGGGATCTTTCCAATTGAGTTTTATTACTTTTTTCATGCATTAATTTGTGGGTATGTACCAATGTCCCGCTTCTACACTGTGCTAGTTTCATCAAACTCTTGATGATTTATATATGTCAGGTTCTCTTTGTATCAAGGATCTACTTCGCGTGTTTGCGTGAAGGTTTTTCACTATGGTTTTATTGAGTCTCGGATGACTGGTCTTTTTCAATTAAGTCACGGTCGACTCCGAGAATTGTCAATATGACTATGAGATGCATGCAAAACTTAACATTTTTTTTGGATATGAGAATAAACAATGCATTTTTTTCCACTAGATGAACAAAAATCACAGCACAGTGACTATTTTCTTAAATTTGTATGAACAATTTTGTAAAACCGGTTGAGACTTAAGAATATCTCATTTGACTGTCGAACGTCGTCTTAGATGAGCCTTTTCATATTTAATTTTGTGAGCTAGTTTGTTGCAATCAGAAGTTGTACTCCGTTAAGTTGTGATTCATTGTTTTGTCTGAAAATGCACAAGGCGTACACATATACTCCCTCCATTCCAAAATAGATGACTCAACTTTTTACTAACTTTAGTATAAAGTTAGTACAAAGTTGAGTGATCTATTTTGGAACGGAGGGAGTACTTATGTATTCAGTAAGGCTAGTTACTCTGATGTGTCATGTTGTTGGATGTATCGTGTATATGCTAAGAGCATCTCTAAACAATCTCCTAAAATTTAAAGGAGGATACGGCCGAGTATCCTTTAGTGGACGAGATTGGCTCCTCTAAACAAGTGTCGTTTCTAACCTATCTACTAAATTTAACAGAGCAAAAAAATCAACACAAATTCCGTGCAAATTTAGACTAGATGCGAACTTGCTTATGTACAAAATTGATTCGAAATAGAAATACATAACAAATTTACACATACTAAATGATATCTAGAACATTATTGCAATAAAACTAATTAAGCTACTAACTATTAGTCTCATTCTCGATCCTCATGCACTCCAATTCGGTGAGCCTTCCGATGAGCAGCCAGGTCCCTCGTCGCTGTCGGAGTCGCTGTAGAGGTCGATGACCTCCGTCCTCCAGGCCCCGTACTGCTTGTACTGCCGGTGCTCTACCTTGATGCGCTCCAGGTAGTAGCAGCGAGCTCCACCATGTAGGCCTCGCCGGCTTGCTTTACCTCGAGCACTCGCGGGCCTAACAGTCTTCATGTGTATCTCGTCGGGTGGCCACCTAGGGGTTGACCGGCTCCAACCGCGGTAGACCGTGGGAGAAGTTGCGTCGGGCGACGGCACTGTGGAGATTGACTAACTTGATGTCATATGTGTGTACATGCACCGCGGAATGGAAGGAGCCAAGACAATGTCGCTTGCCGGTGTCATAGTTCTATATATCTGCGGCCCATGTCCCCCACTGGCGCTAACACACGCCGATCTAATTTGGTGGTTGCGGCGGTGGGGGCGGCAGGTTGGGGAGGCAGGATGCGCTGGCGTGGCGACACTCATGGCAACGTGGATCCGCGGGCCGAGCGCCGGGGATGTGCTATGGGGAGCGGTGGTGGATGGATCCGGCCATTTTCGGTGGTGGGTCGGCGGTGGAGTGGCTATGGGTGATGGTGGGACGCCCCGGCGTCGATTGGGCAAAGGGGCCGGTGGGAGGAGAACGAAGTGGGGTGGAGGATTTTACTCCACGGCTGCTCGGCCGAGTACATTTGTGGGGGGCTTGGAGTACAAAAAATCCTCCCCTAACAATTTTTGGGGTTTGGTTAGTTCCTACTTAGCGGAGGAAAACCAGATTTATCGTCCTCTAACCGGTTATTGGGGATCGGTTAGGGATGCTCTAAGTTCTTAGTTGTATTGTTCTCTTTCTAAAATAAAAGGTGTAGTTATTTTTCTCTGCAGTAATTAACGAGTGAAAAGGTGGTACTAGGAACAAAGAATTCGGGAAATAATAGCTTAATTTAATGTAATTACGCCCCTTTCTTTTGATATTTTACTGTTGCATGTAACGACAAGTATGTGTGTAGATACATTCAACTTAAAGCGAGAGAGAAAGATACTAGCTAGAGTGCATGTGTTGACCATGACATTATACATACAACTTTGTAGTCCTTTACACTTGCGTGGTCATGGCAATATTTATCTGTGCAAGGGATATGATAAATTAGTCAGCAATCCATGAAAAGATTGTGTAAATTAAATATCTCCTAAATAACCAATGGTATGTCGATAAGAGCTGGTTGTATACTACTAGTAGGGCCGGCATGTGATCGAATCCCCAAGCACATGAGACCATGATGCAAACTCAAAAAAGTGGCAAGAGCATATAAGCAGCGATGTATGTGAACATATGGACTTTGTTAGAGGCAAAAAGGAGTTCAAACGTTAGGTGTCTTTTTTCTGTTTTCGTGTTGGGTTTTCACTCGTTTTCCCCTAATTAAGCAGGGAATGTTAGGTTTTTTCGTTTGGTTTTCCTTATAAACTGGATCATCTCTATTTTTCTTAATAAAATGCAGTAATAACACCTGCCCCTGCATTGAGGTTCTTCTAGAAAAAAGTGAAGTTCAAATATATAGTTTTAGAAAAAAAATGCACAGATGTGATTTTTGAAATTACGACGCATGGCATGAGTGAACAAAAGTGATTTCTTGTTCTCATCTGATTATCAGCTGCTGGCAGGCGTTCAATGATTGGTGGCCTTATAGGTTGAATATATATCGTGCATGAAAATTGGTGGAGAAGTACACAGCCGGCCGGATCGATGGGACTAGCAGCAGCTAGCTAGCTCTCACAAAACCATATCATGTACGGATGTGGCGCATGATGAGCATATTATCCATGGCATATTGCATGGAAGTTCGCGTCAGATCGACCACATAAATTACAGCATCGATGCAAGCATATATGGACGACTAAAGAAGCTAGGCAAGGCAGAGGGGCATGCATGATGATGAGGAGGAATTATAGCGGGGTGTGCCCAAAAGCACAGTGGGGTGGGGTGGGGGGTGGGGAGGCAAAACTGTCACAAAAAGCGAAAGGGGGGCTGGCTAGCTAGGGTTTTGTTTGGAATGGTCCAAGATCCTGCCTGAATTCTTGGTACGCGCGAGCATGGCCATGGCCAGCATGTTCAGGGGAATCTGATTCTCCCCCTACCTTTGGCTCGCTTTCATGCCAGGCATTTCCTTTGCTTTGCTTTCCCCAATGCCTATGTGGCAGCAGGCCAGTCTCCTCCTCCCCCCTCTTGCTGTGCTTCTTCACACCCCCTCCCCTTTGAGGGATAAAGCTTGGTAGCACATGCACCATTATTGTTAGTTTTCTTCTCTCCAGATCTATGTGTGCTTCTCTCTCGCCTCTCTCTAGGTAGGGGGTCTTTCATTAGCTAGCCATTGGATGGGAGGGGTTGTTTATCTTGGTCCCTCCTCTTTTTTCTTCTGACAGAACATGATCTTTGCTTTTGCTCATGTTTTGATCTTGCCATGCATGGATGGATGCTTGTTTTACCCCAATCGATGTAATCAACGCAGGGGTTACTAGCTACCTAGCTTCACGTCTTTAACGAATCTCCTCGCCTAGAGATTGCTGGGTGGGTACGATTGTCGATATCACCGCCTTTCGACAGCATTGTCCCACCGATGTAACAATGCAATGTGTGTAAATCATTACCGGTATTTGCATCCTCTCGTGTAAATATTCACCTAGACTGCATTCGTTGTTCACTGAAACTGCTACAGTAAAAGAAGATGGCCTAGGAAAGGGAAGTATGTGCACACAACACATGTTTGTTACAATTTGTCTCAAACCTGCAAGGGACCGTGCAATAGACTATATATATGCAATTGCATTTTTTGGTCCCTCGACTTTTGTCAAAGTCTGAGTTTGGTCATTTAACTCTTGCATTGAAGTTCTTGGTCCCGCAAGTGTTATAAGAATGACAGGTTCAACTAGGCTCCACCTTAGAGAGGGTTTTGGTGACAACGTGCCAACAATTTCACACACATGCATGGCAACCCTATTATCATAAAAGCTACTCTCTCCATTCCTAAATATAACTCTTTTTAGTGATTCCACTACGAACTACATACGGATGTATATAGACATATTTTAGAGTGTAGATTCACTCATTTTGTTCCATATGTAGTACATAGTGGAATCTCTAAAAAGACTTATATTTATGAACGGATGAAGTAACAAAATCAAGAAATGATAAAAAGTACCGATAGGAAACTTTTATGTTGCATGTATTTGTAAAATAGATAAATTAAGTTTAAGAAAACTAGTGAAATAGTATTAAAAATCATAGAGTTTCAAAAATATGCATAAAGCAATATTTAGGTAACTAAAAATATTTAAATGAAATGAAAAATGAACTTAAAAGAAAAATCAAGAAATATAAAAAGGCAATGAAAAAGAAAACTTAGGAAAAAGGAGATTAATAAAAATGAAAATATCATAAAAAATTTAAAAAAATGAATTTGAACATTAAGAAAATAAAATAAAGAAAACCTTTAGTAACGACCAACTTTTTTCTCTGTGGGGAAAACTTCCAATCTATTCATCAAACATCATGGCAGTACAAAGAACACTAGAAATAAAAAATACATCCATGTCCGTAGACCACTCAGCAACAACTACAATTACTAGGGCGAGATGAAGGCACACCTCCATCATTGGCCCTTCCTCCCAGGAATTGGGCAAAGCTTATTGTAGTAGAAAGTCGGGAAGTCGCGTGCTAAGACCTCAAAGGACCAGCGCACCAAAACAGCATCGTCCGTTGCCAATGAAGAGAAGCATAGATCGGAAGGATCCAACCTGTATACGCATGAATGTAGATGAACGAAGATCAAATCGACACAGATCCACCAAAGACTAACACTGACCAAATCACGTGAGATTCGCCGGAGGCACACATCCACAGGCCCTCCAACAACGCGAGAAGCACCTCTGGGATGGGGCTTAGGCGGGGAGAACCTTATTCCATCAGGGAATCATCGTCGGCTGTCCTTCCTGAGGAAGAACACAAACTAACAGACTAAAAAACACCTAAAAATGAAGCATGAGTCCTCCCATCGGCAAGGTCTAGGATCCATCGTGCCTTCATGGCCCTAAGGCCACACGAGACGAGACAGATTGGTACATGTTTTTTCTATAATTACCAAACTCTAATGTTTTTCAGAATGATTCTAGTTGGTATGGATTTTTTTCTAAAAATTAAAATTATTTGTATTTTATTGACATATTTATGTATTCATGCGAAACATGGATCTTAGTTACAGTTGGGAGACAAAAACAACACTTTTATTAAGATTGCTTAATGAACGTCGTGTAGCCTACTGTGATGCCTCCACAGACCTATCTGTTCCATACAGCTTGTGGACCACTGTCGCAAAGCTATTTCTTCTTGTGGCCATTGCGACCACCAGTCATGCAAAATGGCATAATGTTGACAAACCCAATGACCAACTTAAGAGCCACAATCAAAACCAATATTTGAGGCCTTCATCCTTTCTTTAAAGCACGGAGCCCATTCTCAAAGATATAGTAGGAGTACTGCAAATGCTAGCAGGAGATGGTGCGCTTATTATTTTCATTGACAAAACAATATTACATGATACCTCTTGTTTTCTATTATAGAGATTATCTTTGGTTAAAATGAAACCCTTAAATGGGGATTTAATATAAAAAATTATGTCGACAACCCCACACATCACTAAGGCCAACTCCACCGCACGACCCCAAACGGACGTCCGGTTTGGCCGGATTTTGTCCCTTTGGGGCGCCGATGGGTTCGCCCGTGTCCGGCTTTGTCAGATGGGTCGTGCGTGCGCCCACCGCGCGACCGCACCCCAAATCGTGTCCGGGGTGGACGTGAATAAAAAAATATAAAAACTAGAATGAAATAACTAAAAAGGTAAATAAACGCATTTAAAAAAAACATAAAACATATATAGGGGTCGGCCACAAAATGGCCCAGTTTTCACGACCACTTAAAAGGCCCAGTTTCATAATTAACATATAAAAAAAACGCCTCCCGCGCGCTCCTGCCGCGCCTGTCGGTGCCGTGGCCGTCCCCGTCTTCACTGGCCGCCGGTGTCGTTATCGTCGCTGACGAGGTCGACGTTGGCCGGCGGCGTCCACAGGTGGGGCGGCAGTGTGTGGTAGACGGGGGCGGGATGGACGGCCAGAGGTGCCTGCACCACCTCCTCCCGCGGCGACGCCTCCCGCTCCGGTGACCGCGGCAGAGTGGGGCACCAGTTCACGCCCAGGCCGGCGGCCATCTCCGGCGCAGTGCAGGACCAGCCCCACCCCTGGCCCAGCAGCTTCTCGAACGCCGCGGGTCCTCCTCCATCGGCTCCTCCGTGACGGCCGCCGTGACAGCCGCCAGCTGCAGCTCCGGGAAGGCGACGTCCCCGGCGGCAGAGAGGGCCATGGCCTCCTCCAGGCCGTCCCACTGCCGCTCGTCGTGCGTGTTCATGGAGTCGTCCATGACACGCTGCAGGAGACGGGCCTCCTCCTCCGCTGTCATGCGAGGAGGGGGAGGGGGCGACGGAGACGGGGAAGGCGACGGCATGGGCATCAGGCCGCGCATCCGCGTACGCCCGCGCGGCTCTGCACGTGGCCTCCGCGGCCCCGACACGGTGCCGGTGAAGTAGGACACGCGGCGCGTGTCGTGCTCGTCTTGGAGCCATGTGTCCCAGAGCGGGGAGTCGGGGGCATACCTGTGGTCGTAGTACAGGTCGTCGGGGAGGAGGCGGCGGCGGCGGTTGATCTCATCGCGCCGTGCACGGCCGGTCGCCGGCACTGGCGGGATGGGGACCCGGTCCGCAGAGAGGTGCCACCCGTTGGGGAGGTTGCCGTCTCCCAGTACCTCCGGCACACGTCCGCACAGATGTACTGCTGGTCGCGCTCGCCGGAGGGCCTAGGGGCAATGGTGAAGGGGGCCGACGCGGGGGCTCGACGGGAGGAGTGCGGCGGTGACGCGGGGGCTCGACGGGAGGAGCGCGGCGGTGACGCAGGCTCCTTCTTTTTCACGGATCCGCGGCGGCCGCCGGAGGAGGAGCCGGACTCCCGGTCGTGCTTCCCCTTGCGGTTCCACAGACTCATGGCTGCGGCCGGCTGGCGAGCTCGAGGGCGGGGAGTGGCTAGGGTTTGGGCGTGTCGACTTTCGAGGGGGCAGAGAGGGGCCGGCGTGGGGATGAGGACGACGACCGGTCCACGGGTCCCATTTAAGAAGGACGCCGACCCGTCGCTGTGCGGATGACAGGTGGGGCCTCCCGCCCGTGCGCATTTATGTTGGCGGGTGGGAGGTAGGTGGCCGCCTGCCACGCGGCCCCGACGCGGACGTTCGAAGCGTTCGTTCGCTGTCCGCCGCGACCCATAACCAGGCGCAAGTTTGCGCTCGAAATGGGTCGGCACGGACACAAAACGGACCAGATGGGTCCGGACCATCGCGCGCTGGACCGTCCCATTTGTCCCTTTTATCCCAAACGGACGGGGCCGGACAAGATAGGGTCGCGTGGTAGAGTTGGCCTAACAAGTATGCCCTCAAGCCATCCGTCAATAAAACATGACGAGACCTTTTATCAGGAGTGAAATGTTAATATTGTCCCAACATCGAAAGTAGGGGCTAGGTGAGGGAATTCCGTCTTTTGTCAAGGAGCAAAGACAAGACATTTTGTCAACATAACCCGGCCCCTGGATAGATAACTCAAGATATTTTTTTAAGATGTATATTTTAATTTTTCAAGTGCATACTGAATATGGGGATCCCTAGCTAGAGCTTCTATGACAATTGTTTCTTGAACAGTTTTTGTAGAAATCACATGAACCCCACCCTTGATCTCTTCCACGATGCTTGACATTTTTCGTCCGTCTCGTGGTGCGGTTTCTGATGACCATGTACTCTACTATGTACTCACTTTGGTTTGTCAGACAGCAATGGATTCCTGGGTCATCAATTTGGCCAACGAGACATAACTTCCACTCTGTTGGTGAAATTCATGATCTAGAAATATGTGTTTGCTTTATAAACTGAAAAGGAGGAGTACTCGATATTGTTCCTGTTCCGGCACTGCATTTTTTCAGTTCCCATGTGGTCAAACGACTGGCATCATGCATGATTTGTGTTCCCCTCTGCCCCTCCCAAAGATCACTCGGTCGGCGAGAAGGGTCGATGATATATGTAAAGCAAATGTACATTATTCCGCTTATTAAATTCTGCAGTGTGGCTGCGGAGATCACCTCATGTATGTTTACATACGCGCGGTCCTAACGTCATAATATACTACTCCCTCCGTCCGTATTTACATATCGTCTTTTATGTCGTCGTGATTTAACTTTCACCGATGATTTCATCAAGTAATACCTGAGCTGCGTGGCATAGATATCACAGCATTGGAAACTATTTTCCCATACAAATTCCAATCGTATAATTTAGGTGGCATATAACCCACGATTTTCGGTGAAAACGAAAGTCAAACTTAAACCTCGAAACGGGTTCCATGCGGACCAGGATGACGGAAGGAGCAGTACATTCCATTCCATGCTCAATGTAGCATCATGATATTCCAGTGCTGATGCCTAGCTAGCTGCGTCGATCGTAGTATATCCTGCAGTCGCAGTCGCAGATATAATCATCAGGTTCCTTCGTATATTCTACAGATCCTTCCTTTTTCTCTCTCTGTTTGTTTGTGCTGCGATAGCACAGATGGTACATGCAGGATTTTGTGCTGGCAAGAAACATTAGTGAGGTCACAAGTCACATCGAACCTTGTCAGTTCTGGCACTGCATCTGCATGCATGTTCAGGGTTCAGAAACCGATCTTTTCACATGTTGCAGACGGTTTCTGCATTCCCATCACAATCTAAGGGTTAAATTTGTCTAATTTCAAAATCTAAGAAGTGAAACTACAATCAATAGTTGAGCACTGAGGTTAAGTCCGTGCTATATATGGTGGTTACGGGCAATGTACGTAGCCGGTCATATACGTGCCTTGGCATGTGGGTGAAGTAAGTATGTACGGCAGTGGCTTCTTTTTATCGATAAAGGACGCTTTTACAAACACAAAATATAGCATCGAGTTGATACAATTTATGATGAACAACACCTGACCTCTGCATAGCTAAGATGCCCACAGCCAAAAACAAGCAGTCTGAAAACATAAAAATATAAAAAATGGACATATCGGCACCAATAGAGTCAAATAGGACCGTCACTGTGCCTATATCGAAAGAGGTGATGGACCAATACCGAGGTTATGATACAGCCCATGTTGAGAAAAAACTTCCATGGCTCCAATCGCGTACACACCGCCTTGAACGGCGGTTGATACTACGTATGGTGAAGCATAGACCACATGGCGAGTGCGTACAACAAAAAATAATCTGCAAAGGAGAAGACTTTTTGTCCTTAAAAACCAAATCAATTCTACATAAAACAAAAAGACGATAATAAGACATACGCTCCCAGCTTTAATACTAGCGTTTTGAACCTATAATAGAACGGCAGTGTCTTCTTGCCTTCTTCCCATCGATGATACGAAACAGTCACCATGCACGTACGAGTACGAGTACGAGACAGCAACCAAATGCAAGTTTCAGAGTCCAGTATGCTATATACGCACGTATTGCCTGAGTACACTAACAAAGCTACACAAATGTTTTTCTTTTTTGAAAAGAAGAAGAGCCCCCAAGGACCCAGACCACCATTTACGACACTTTCAATGCTGACCCGCAAACCGGACACCACATCCATCCACGGACCAACGGGACCAGTTCGCGGACATGGATGAGAGAGCCCGCCATTCAAAGCTAGCTGAATACTATCCGCCAATAAAGTAAATTTGAAATTCAATCTGTCTGAGGAAGCACGCACTGGATCTCACAGAAGCGGTGGACCACATCATTGCATGATCACAACAAAAAAGAAACAAGTATGCTTAGTTTAATCTGACACCGTGCAATCCGAGCTTCCATGTTGAATCTGCCGTCGCCACCATCTCGTGCCGTCGCCACCATCTCGGCTGACTCCACGCCGCCATCTCCAACTCATTGTCCTGCCACGGCAACATCTTAAAATGGTCGGCTTGCACAATAATCCCTGCGTCCTCATCCAAATCTTCCATCTTCAAGGTGACCATATACTCTAGCTTATTTTCATAACTCATTTAGTATTTTATGTACTCTAGCTTATTGCATAATGGTTGTTGGAAAATTAGTTGTTCATAAAGAGAACAATTGTTTTTCGTTGTTATGCATAGTGAAGGGCCATACTCTTCTTATGTAGTATGTATGTTATGAATATTGACAATAAAGTTAAACATCCATAACATTGGCGCTAAATATCATAGCACTAAATGAATATCACAAGTGTGTGTGGCACTGCATTTAGTCACATTGTAGAAATACACATGGAGAAATTCTATAGTTATGAATTTTAGAGTCATCTGATTACGTATCATCTGACACTTGCAACTTTCACCCAAATAGTGGAGTAACTAAAATATCGTTCACAGGCATTAAGAACAAGCAACAAAAATGATAGAAGCATACATGCTGATGTGTGTAATTCAGTAAGAGTTGTTGCAAGCGGTGGATTTATCTCTCTTCAAGAATGACTCAAGTAGATATATGGATATTTTCTTGTTGGGACATAAGTCTAGATCCTTTAAAATAGTTAAACAGATTTTCAGAATGAAGTAGAAATTATTATAACAAGATAAATTATGTTTTTGCAATTAGACTTAAAAGAGGAAATTTTGAGTTACAAGTTTAATGAATATATGATGATTTGTGAAAAGAGCTTCATAACTTGCACCTCCCGGAACAACACTAAGAGTGGAGTATCCAAGAAGATGTAATCAAATCATGTGTGACATGGTGAGATCAAAGACAACACAAATCAATTTTCCATTATACTTTTAAAATCTCATGCTTTAGAGACTGCGACATTTATAGAGTGCTATCAAATCTATTGAAAAATGACGCCATATAAGGTATGGTATGGTATGTCAAACAAATTTTGTCTTTTCTTAACATTTGGGAATATGAGACATATGTAAAAGTGTTTACAAGCTGATGAGCTCATATCCCAAGCCGGATTAGTGCTACTTTGTTAGTTATCCCAAAAGATCGTATTCCTCTATCACTACACCGAGGCAAAGTATTTTTCCATGGAGTGCGGTGAATTTAGAAGAAAACGTTTCTTGCAAAAGAATGAGTGGGAGAACAGTGCAACTCGACGAGATGACAGAATCTTCGTAACCAGGTCAAAGGGAAGAAGCGTTAGAAGTATTTCCAAAATTTCCTACTTTGACTGATACGCAAGCCTCTACATGAGACATAGACTTCGATCGAATTTGCAGCTAAGCCACATGGGTTAGGCAAAACTCATACAGGCCCGTGTGGTATGCAGACGACAAAAAATTAATCTATGTACACAAAGAAGCATTGATGAGACCTGACTCTGAATTGGTTATATGTCAAAGTAATCCAAGACATTATCCATACATGATTCCAGTTTAGAACTTGATGAACCCTCCAAAAGACTTAGAGTTTCACGTATAATAAAGGATATGTAAACCGACAAAGATAAAAATATTTTATAAAGTTGGACTTGTTTCGAATAGTATATGACAAGTTCACATACTTGACTACGAGAAGATTGTCTCATTGTATTGATGCTTAAAGTCACTTTCGGTTAAACTAGCAATTACTACATATTTGAATTATGAGATAAAAAATATGGACGCCAAAAGGATTTTCATGAGAAGGAAGTAGATCCAAGGAGATTTTGTCGATCAAGAGGATGCTAATAACTGTGCAAACTTCAAAGATCCAAGAGTGGACTGAAGCAATCATAATGGAGTTGGACTCTTCGTTCTGATGAATGCTCAAGGAGTTGGATTTCATTGGGGAAAGTGAAGATACTTGTGTTTACAAGAAATTAAGTGGGATCTTAATAATATTTTTCCCTTTTCGAGGAGCACATATGTGTGCCTCCACGAGATGCAAATACGCACTTCTCGTGGAAGCACAAAAATAAAATCTTGGAACAAATTCCCTTTCCGAAGAAGCACAATCGTGCTTCTCGTGGAAGCAAATATGTGCCTCCTTGGGAAGCACATATTTACAAAAAGAAAAGTAACTTCACTAAGAAAGATTGCTTCTCGCAGAAGAAAGTCTATGCCTCCACGAGAATCAAATATGTGTTTTGTGAAGAAGAAGAAAATTTGCGTAGAAAAATCATTTTCCCTTTCCGACAAGCACAAATTTCTGAGAAGCAAATATGTGCCTCCACGAAAAGGAAATCCGTGCTTCTTGTGAAAGCAAAAAAGAAAATCACAAGAAAAACATTTCATGATTTTTTTTCTCTCCAAAACATAGGGAAAAACCAGGCGCAAACTGAAAATCCAAAAAAGAAAAAAGAAAAATCGCATCAAAAATACAAAAACATGTATGGAAAAGTAAAAACCGGAAAATAGAGGGACCGCCTACCACGTGACATGTGAGAGCGGTTGGACATACCACTTGGTGCACTCTCAGCCCACAAAAGTGATGCTTGCGGGGCTCCCGCATGGGGTACCCCTTGACTACTTGCTCTCACCAAATAAGAGGACCTGCTCGGAACGGCGAACTGTCCGTAATTTGCTCAGGCTGGGATGCCAGCGGCAAAGGCATGGGCCAGCCCACTTGCAAAGCCATACGCAGTGCGGTCAACCTGTTAGACATTACTGGTCCCTTTTTTTTCTTAAATTTTGTTTTCATTTTTTCTTTGTCATTTTTTTCTTCTTGTTTCTTATTTCTTTCTTCATTTGCTTTAGTTTTTCTTTTTTTTTGCATAAAAAATTTCAAAAAATGTTCACAACTTACAAAACATGTTCATGTTCTTCATAATATGTTTGAGAGTTTCAAAAACGGTTCCCGTAAAAAAATTCTCAGCAGATTTAAAAAACGTGCATGTTTTAAAAATATTGTTCCAAACTTTATGAAAACTTTCCAGTTTATAAAAAATACTCAGGTTTAGTTTTCTTATTTTGTATTTAAAAATATGTGCATGTTTTGGAAAAATATTGGGAGTGTCAATAAATGTTCCTTAAATGTGCGTGTTGTCAATAAATGTTGCATTTGGAAAAAAATATTTGACTATTTTAAATATATTATCCGAAATTATGAATGTGCTTTCTTAAAAAAAATCTAGAGGGATCAAACCCAAGATTTTCCACAGCCAGTAGTTCAGAATTTGAAGAAATCTTTGGACCTGCACTTGAACTCGTTCTTAAAGGGTTTTCTGAACGCGATCACTTGTCAAATATCTTTGGATTTGATTCCTCCAAGAATTATTTTTGGGCATGGACAATTTTCAAATTTGTGAATACATTTTGAAAGATTTATAATATTTTCTATAAAATGTAAACCTTTTTAAAGATCATGATCTTTATAAGAGGAAACATTTATTGTAACTTCGGATTATTTTTGAAAATGTGAACATTTTTCAGAATCCGGATATTTCTTGAAATTCCAAAAAAAAATTGGAAACATAAATAATTTAATAATACATGAAAATATTGAAACTCCGGAGCACTATTTGGAAACCACAAACATTTTCTTAATTTTGGGAACAATTTTTAAAAGGGAACACTTTTTGATATCCCCAATCAAAATTTGCGGAACAGGAATATTATTTTGAAATATGCGACCTAATTTTGAAACTACTGGCCAAATTTTGAAAACAAGTGTGTTTTATGAAATTTTGAACATTTTAACACGAATGTTTTGCAATTGCTCATTTGCAAAAATGCAATCATTTCTTGAAAAAGAAAAACCAACTTGAAGAAAAGGAAAACAAAAAAGAAACAAAAAATGAAAATGAAACAAAAACAAAAACGAAAAATAAGAAAAAAAACCAATAAACAAAAAAAGTAAAAAAAGAGAAAAAACAAACAAAAGAGACCGTAAAACAAACAAAAAAAGGGTTCGGGGAAGAAGTTTTTTAGGGTTGTTTGCGGGGCCTTACTGTGTGTATACCGTTCAATCCGCCGGGACTTGTCTCGCTTGCATCGCTATCTTTGCGAGTTACCGATAGTTCTCCGCAGCGAGCGGCAAGGCAAATGTCGTGAATGCTATATATCGTGTCAATCGAGTTGCAAGGTTCTCTCGCTTAAGCATCTACAATGATGGGCCGGCCCATTAGCTTATATACATAAAAGAGAAAATGAGCAGGGAAAACACAAGTACCGGGATTTGATCATGGGACCTCCATGATAACAGACAGCGCATATAAATTAGGTTAGCTATCATTTCCTGTCAAACAGTAGGGGCCCGACATATGAGAGTAAATAACATCCGGTTTTTCAATGGTTTTCCTGGTTTTTTTCAACAGGTTTTTTCTATCTTTCCTTTCTCCATTTTCTTCTGTTTTATTCAGTTTTCTTCAGGTGAATTTTATTTTGTCGTTTCTTTTCTTTTATCTTGTCTGTTTTTATCATTTTCTTCATTTGTTTCTTTGGTTTTATTTCCATTTTCTTTGTTCTTTTGGTTTATTTTGTTTCTTTTACGGTTTCAATTTTTTGTTTTTCGGTGTTTCTTTTGATTTTCATTCTACATTTTTTTATATGTCAACAACATTTTTTCTAATACAAGTTTACCATTTTTCCAATACAAATTTAACATTTTTCAATACATAGTCTACATCTTTTGTATACACATCTTAACATTTTTCAATGCTTGATTAACATTTTTTAAATACAACATTATTTTTTTCCAATGCACAAAAAAAATCAAATGATTGACTAACATTTTCTAATTCCTGGTCAACATTTTTTTATGCACATTTGAACATTTGTCAGATACTTGATTAACATTTTTTAAAATGCAATAGTAACATATTTTTAAAACATGGTGAATAGTTTCTCTAAAGGCATTGTACCTTTTTCAAATACTTGTTTCAAAAATTTCAAATTCTTGATTAACACAAACTCACACACACACACACACACACACACACACACACACACACACACACACACACACACACACACAGAGACACAGACACACACACACACACATGAGAAAATTTGTTTCATTGTTTTTATAATATGTGGTCAATGTTCTATCTATACACATTTATCCTTTTCCAAGTGCTTGCTTAACATTTTTCAAACACTTGTCAACATTTTTTCGGATACTTGATATTTTTTAAATAGATATAAAAAAGTATTTTGTGCACAGTTTATTTTTATATACATTTTTTGGATATGTGATAAATATTTTCTCCATACATATTTAACATTTTTATAATGCTTGGTCAACATTTTCAAATGTTTTAATGTAGAGTGGTTTTGGTAATATACTTATTTTTAATATTTGAAAGTATAAAAAAGCAAAAGAAGAAAGCAAAAACTAAAAACGTGAAAAAAAGAGAAACAGATGACGTGGCTGTTGGTTCCCTCGCGCCAGGGCGGCCCTACTGGTGGCTACCTTCAGCGAGACATAGGGGCGCCCGTTTACGATGGTGGCATGCTCGCCTACTTATTACACCCTTCATTGCCGGCCCACTACAGTCTGTAGGTTTCTCTATAGTCTTCACGTAATTTCATAATAATATTGATATGTTAAAAACATTCATGCCATAAAAATTGTTCAGATGTGAAAAACAATTTCATGACTTTAATCAATATTAGTATCATTCAAAGATGCTTATGACATTTTAAACTGATTTTATCATGTATATTAAGAAATGATGATCATGCATTAAAACAAATTCATATTGTATTTTAAAATTACTTTGCTCTCTATATGAATATACAATGTTTTTTTTGAAAATTATTCTTAATATATTTAAAAAATGGACGCTGTGTTTTAAAAATTATTCTTCATGTATATAGAAAGTGTTCATAACACATAAAGATGCTACCCGTTTAAAGATATTTCATGAAATAATTTAGAAATATTCAATTTGTGTGCATTTATATTTTTATCTTTTTGAAAAAAAACTTATGTGCATTTAACATTTGTTCATGCATTCTATTGTTCATTATTTTTTGATATAGCTCACCATTCATTCCAAATGGTAAATGTTCATGTTTTCAGATAAATGTTAATACTGTATTTGATAATGTTTAGCATGTATCTTCAAAATGTTCAACATATATACGATTTTTGTTTCAACGTTTATTTAAAAGGTATGATGTATTTGAAAACCAAAAACACAAAACTGGTAAAAACCAATAAATGAACGTTTAAGAAATGAAAGAACTGATTCTGACTTTCCCAAAACCAATAAGAACTGTACTTAGAAGCTTGCAAAATTAATTCTATCTGGTTAACACATCAAACGAATTATTGGAGCGATGCGATACTAAGCGAAATATAGTACTGCCACCTTTTTTTTCTGAGAAACTGTACTGCCGCCCATTTCCAACATCACAGTGATCCGTAATGGGCCATGCCATGTACCAGGTTTTGTAAGCATACACCAGTTTCGGGATGCTTCCAGGTGGCTTTTTCTATGTCAGTTTCATGCTTTTTTGGTCAATTTTTTATTTTTACTATTTTTTGTTTGCCCATCCTCTTTCTTTATTACTTTGATTTTAAATCATTTTTTAATAATTTCGTGAACCATGTTGAAATTCATGAACATTTCTTTGAAAGCAGTAATAATTTTTGAATTCATAAATACCCCCTCACTTTCTTTTTACTCTGCTTACAAAATTTGTTTGAAATCAAACTACGTAAAGTTTAAACAAATTTATCCTAAAAAATATCAACTTTTACAATGATGCATCTAATGATATTGATCAATATGAAAATTCTTTTCATGATGCATCTAATGATATTGATTTCATATTGTGAATGTTGATTTTTTTCCTTATATAGTTGGTCAAACTTTTAAAATTTGACTTTAGGAAATTTTTGAATGCATACTAAAAGAAACGGAGGGAGTACTTTCTTGAATTTGTTACTATTTTTAAATTAGCAAACATTCTTTTGAATTCACCGACATCCTTCTCTATTAGTGAATATGTTTAACTTTTTTTAATATTTTCAAAATAAGTGACCACTTTTATATCCGTGATATTTTGAAAATCTATGAAGTTTTAGAAATTTCTTAACAAGTTCTAAAATAGATGAATATTTATTATATTCATATAATAATTAAATTTATAAAAATCAAATCATTTGACATATTTTATAGTATTGAACATTTTGTAATAATAGTGAAGACACAGAGCTATTTGTTCCATGCGGTGAACTGGTTTCCTAGTTTTTTACGGGTTGAACCAGTTCATCCATTAATATGGAAATGTACACCTACGGAAGTAAAGCGAACACTATGTCTCCAAATGGGCCGGTGTGAGTCCTCTCACGCTCCTATACGGAGAATTCCCCACTTAAAGCAGGCAATAGGACCGACGCCTCCTATTTCAAGCGGTCTCCGTCTATTTGTCGTCGAATCTCACATAGGAATGGGATATTGTCCAGCCCATGAGGGCCTTCGTGGAGTGCTCAACTGAAGCGATTTTTTACCTTTGGTTTGCCTATGAAAAAAGTGACTGCTTCTTCAGATTTGAAAATTTATTGCATCATATAAAAATGATTTATTCTAGATAGTGAATTTTTACAAGGTGAACACTTATAAATATTTGAGAACAATAGTTGAAAAACGTTCACATTTTCAAAATATACTGAATAAGTTTTTGAAAAGACATGCAGATTTTTTGTAACTTGTGTTTGCAGACCACTAAAAATACAAACATATTTGAATAAATGACAATTGAAACATTGAACATTTTAAAAAAATTATATGCTTTTTTAAAGAGGAAATTTTCAAAAAAGTGTGAACATTTGGTGAAAACAAAAAAAAAACCTAAAAAAGAAAAAGGGGGGGGGGTACTAAAACCAGTAAAAGCAATAAAAGAGAGAAAATACAACCCTTGAGTGTTGCTCAATTTCTTTGCTGCGCTGCTCATGCGCTGGGATGGCTAATAGTCACCTAAGTCTACCCTAGATTGGTGGCAGACCATGCGTCCCTAGCCCTAGTAGTAGCCGGCAAAGCTTGCCCCCATGCTGCTGCATTGGTGAAAGGATCGAGATGGACCTAGAAAGGGGGGGGGGTGAATAGGTACAATTACAAATTTTAATTATTACTTAGCAATTTTAGGCAATAATGCGGAATATGAAGGTGAGCCTAACAATTGCAAGTGTAGTACTAAGTGCTAAGCAAGATAAACAAGTGGCACAAATATATGAGAAGCAAGCACAATATGATAAATGTAAGTGCAAGAGACAAGTAACCACAAGTAGGGAGTTAGGGTTAGGAATAACCGCAACTCCGAGAGACGAGGATGTATGCCGATGTTCACTTCCTTGGAGGAAAGCTACGTCACCGTTTAGAGAGGTGGATGTTACCACGAAGGTACACCAACGCCATGAAGGCTCACCCTATTCTCTCTTTGAGACAACACCACGAAGACGTTTCTCAACCACTAGTGGTAGACCTTTGGGTGGTCTCCAAACCCTCACAAACTTTCCGGGGGTAATCACAATGGTTGATTCCTCACCGAATGACTCCTACCGCCTAGGAGTCTCCAACCTCCAAGAGTAACAAGATCCAAGGGGAAAGACTCAAGACTTGCTCAAACCACGATTTGCTTTGGTCACAAGAAGGAGAGGGAGAAGATCTTTCTTTTGATTGGAACAACTCTTCGAAACTCTCAAGAATCAAGCCGGGATCTAGGATTTAGTATATGAGCAAGAGAGGGGGAGCACATGTGTTCTTGAGGCGTGTCTGTGTGAAGTGTTCCACCTTATCTCGAAAAGGTAAGGGACTATTTATACCAGGTCCAGAATTGAGTCGTTTGGATAGAAGTTGACCAGCCCGGATGATCTGGGACACCACCCGGATGATCTGGACATTGTCCGGATTAATCTGGCTGAAGGACCGAATGCAGATGGTAGCGGCAGAGCTGCGAGTAGCCCGGACAGACCGGATGTCACCCCGGACGATCCGGACATATCCCGGATGATCCGGATGCCTAGCCGGACGATCCGGCTACAACTATCTGGGTTCTGTGAAAAACAGGGGCAAGATTTCTCGGATGATCCGGAACAGGATTCGGACAATCCGGGGCACAGCCCGGATGATACGGAAGAACGGCTGGACGATCCGGCTTCATCCTGCTTCAGCCACACCGTTCTATGATGAAAACCACATATTATCCGGATGATCCAGAGCAATGTCCGGCTGATCCGGGTTTAGCGCGGATGATATGGGGAGCTGTCCGAATGATCCGGCTAGGAGAAGTGGTAGGTTTTGGCTAAGGGGTTTTACTAGGGATTTTGGTCTTTTGTGAAAAATTGTTTGTGAGAGAAAATGTGAGCAAGTCTTGAACCTACACCTCAGATCCCCTCTTAATAGTGCGGGATTCCTATTACTCAAGAAATATAAAAGAGCACAATGCTTCATCTTTGTTTGCTCCTTTCCTCATTGATAATTTGCTTGAGTAAAATCTCAGATGATGTGATCATGATGCTCATTAGAAAAAAACCAGAGGACTGACGGCCTGTGTATATACTTAGAAAACATGGTTAGTCCCCTATATGTAGATTTGTCATCAATATAAAAAATATTCTTTAAGAGCAAGATTGCACTTTCAATCTCCCCCTTTTTTGGTAGTTGATGACAAACTACATATAGCACTCAACAAGTGAAATGTGACGCGGATAATTCTAGGAGAATAGTCTGGTATGTGTTTTAGGAAGAGCTCCCCCTTAAGCATACTAGACACACACTCATACTCCTCCTAAGAATTACATCTCCCCCTTGACAACAAGTACCGAAAAAGAGAAAGACGACCGAGAAAGTAGGAGGATCACTCAGCATCCTCCTCCTCATCATCATCACCATCACTCAGATCAGCAGGTGCAAGGAAGAACTCGGGAAAATCAGTGACAGAAGGAAAGTCAACCTCCGGAAGAGGAAGCCGATCAGGAATAGGCTCATCAGGCTCGACAGGACTGCCAGGACGCGCACGGAGGCGAGCCCTAAGAGCATTTGCCTCAGAGATGCGGCGAACCTCCTCAGTGGCAATGTGAGTAGCGGTGTAGCGGCAGGTGT

**Supplementary Figure 5.** DNA sequence similarity between *TaDof-B1* in ‘Chinese Spring’ and *TaDof-B1* and *TaDof-B2* in ‘Paragon’. (**a**) Diagramatic representation. Interconnecting ribbons indicate regions of sequence homology. (**b**) The ~73 kb genomic sequence on chromosome 3B (3B: 9079519...9152689) encompassing *TaDof-B1* and *TaDof-B2* region in cv. ‘Paragon’ (Ensembl assembly GCA949126075v1). *TaDof-B1* and *TaDof-B2* share 100% genomic sequence similarity in Paragon. Sequence similarity of >99.99% extends to encompass the regions 4,138 bp upstream and 17,865 bp downstream of *TaDof-B2* with the equivalent region spanning *TaDof-B1*.The reverse compliment strand is shown. The upstream and downstream regions showing >99.99% sequence conservation between *TaDof-B1* and *TaDof-B2* are indicated in purple and grey, respectively, and includes two 1-2 bp insertion/deletions (InDels) in regions of low complexity (highlighted in brown) and a single nucleotide polymorphism (highlighted in blue). For each gene, exon-1 and exon-2 are highlighted in yellow and red, respectively. The canonical intron splice donor (GT) and acceptor (AG) nucleotides are highlighted in red. Note: the duplicated 25 bp sequence insertion (agatgtcttagaacgtcgtcttaga) identified downstream of two of the three duplicated *TdDof* genes in durum wheat by Nilsen et al. (2022) was not present in the corresponding regions downstream of *TaDof-B1* and *TaDof-B2* in bread wheat cv. ‘Paragon’.
