# Supplementary material for: Association mapping of wheat distinctness, uniformity, and stability traits identifies evidence of TaDof-B copy number variation associated with stem pith thickness
Source: Front Plant Sci. 2026 Mar 31;17:1739489. doi: 10.3389/fpls.2026.1739489 (PMC13077854; doi:10.3389/fpls.2026.1739489)
Supplement: Supplementary file 2 [file DataSheet2.docx]

**Supplementary Text**

*Heritability*

The model details are similar to a previously used model for estimating genomic heritabilities in barley DUS traits (Yang et al. 2021). Briefly, we fitted a fixed intercept and a random additive genetic effect term under the assumption of a normal distribution with mean 0 and variance proportional to the additive genetic relationship matrix derived from the wheat lines used in this study. Model fitting was undertaken using the *mmer* function in the package “sommer” (Covarrubias-Pazaran, 2016) in RStudio (RStudio Team, 2020). The *vpredict* function in the same package was used to obtain the estimates and standard errors for genomic heritabilities, which were calculated as a ratio of additive genetic variance over phenotypic variance (sum of additive genetic and residual variances).

*Regional annotation*

To further annotate the 828000748-828207481 region of chromosome 3B, the *TaDoF-B* gene, *TraesCS3B02G608800* (RefSeq v1.1, Alaux et al. 2018) was used as a query for BLASTn searches against 12 chromosome-level wheat genome assemblies available from EBI: ‘Arina*Lr*For’, ‘Jagger’, ‘Julius’, ‘LongReach Lancer’, ‘CDC Landmark’, ‘Mace’, ‘SY Mattis’, ‘Norin 61’, ‘CDC Stanley’, ‘Chinese Spring’ RefSeq v2.1, ‘Kariega’, and ‘Renan’ (Walkowiak et al. 2020; Zhu et al. 2021; Athiyannan et al. 2022). For those genomes where *TraesCS3B02G608800* was present on chromosome 3B (all except Julius, for which *TraesJULUn03G04572240* was not scaffolded within a chromosome), distances of 160.866 Kb upstream and 145.866 Kb downstream from the start and end of the genes were used to extract feature positions from each assembly genome feature file. Liftoff (Shumate & Salzberg, 2020) was then used to annotate CS v1.0 chromosome 3B with genes present in the 11 genomes containing *TraesCS3B02G608800* on chromosome 3B. Further manual investigation and curation was undertaken using the tools available at Ensembl Plants.

*KASP marker development*

Kompetitive Allele-Specific PCR (KASP) molecular marker primer design was undertaken manually based on genomic DNA alignments of the available flanking regions of each chromosome 3B target SNP (Wang et al. 2014a), and those of the homoeologous regions on the A and D wheat sub-genomes identified via BLASTn searches of the wheat reference genome (RefSeq v1.0) using Ensembl Plants. KASP primers (Supplementary Table 3) were ordered from Merck (Merck Life Science UK Limited, Gillingham, UK) and used for genotyping a subset of cultivars from the ‘WAGTAIL’ association mapping panel following the methods described by the manufacturer (https://3crbio.com/product/pace/). At least three KASP genotyping technical replicates were undertaken per cultivar.

*CNV assay primers, reaction volumes and thermal cycling conditions*

The primers TaDof-B-F1 (5’-CCTCCTGCCTTCCTCGAC-3’) and TaDof-B-R1 (5’- TGAACAAAATCTTGCGAGAAG-3’) were paired with the amplicon-specific probe TaDof-B-P1 (5’-CCAGCTTCAGGTATGCATCCTTGC-3’) tagged at the 5’ end with the FAM fluorescent dye (Sigma Aldrich). The primers for *GAMYB* (5’-GATCCGAATAGCTGGCTCAAGTAT-3’ and 5’-GGAGACTGCAGGTAGGGATCAAC-3’) were paired with a probe (5’-CGTGGCTCCTGCGATGCAGC-3’) tagged with the VIC fluorescent dye. TAMRA was used as quenchers for both probes. *CNV* assays were undertaken in a total volume of 10 μl consisting of 5-10 ng of genomic DNA, 100 nM *TraesCS3B02G608800* primers and probe, and 100 nM *GAMYB* primers and probe. The real-time PCR conditions were 95 °C for 15 minutes, 40 cycles of 95 °C for 15 seconds and 62 °C for 60 seconds.

**References**

Covarrubias-Pazaran, G. (2016). Genome-assisted prediction of quantitative traits using the R package *sommer*. *PLoS One*, 11, e0156744. doi: 10.1371/journal.pone.0156744

RStudio Team (2020). RStudio: Integrated Development for R. RStudio, PBC, Boston, MA URL http://www.rstudio.com/.
